# Development of a DNA barcode library of plants in the Thai Herbal Pharmacopoeia and Monographs for authentication of herbal products

**DOI:** 10.1038/s41598-022-13287-x

**Published:** 2022-06-10

**Authors:** Santhosh Kumar J. Urumarudappa, Chayapol Tungphatthong, Jirayut Jaipaew, Natapol Pornputtapong, Duangkamol Pakdeesattayapong, Sornkanok Vimolmangkang, Suchada Sukrong

**Affiliations:** 1 Center of Excellence in DNA Barcoding of Thai Medicinal Plants, Chulalongkorn University, Bangkok, 10330 Thailand; 2 Department of Pharmacognosy and Pharmaceutical Botany, Faculty of Pharmaceutical Sciences, Chulalongkorn University, Bangkok, 10330 Thailand; 3 Department of Biochemistry and Microbiology, Faculty of Pharmaceutical Sciences, Chulalongkorn University, Bangkok, 10330 Thailand; 4 Herbal Products Division, Food and Drug Administration, Ministry of Public Health, Nonthaburi, 11000 Thailand

**Keywords:** Plant biotechnology, Sequencing, Plant molecular biology

## Abstract

Traditional herbal medicine has long been practiced as a method of health care in many countries worldwide. The usage of herbal products has been increasing and is expected to continue to do so in the future. However, admixture and adulteration are concerns regarding the quality of herbal medicine, including its safety and efficacy. We aimed to develop a reference DNA barcode library of plants listed in the Thai Herbal Pharmacopoeia (THP) and Monographs of Selected Thai Materia Medica (TMM) (n = 101 plant species) using four core barcode regions, namely, the ITS2, *mat*K, *rbc*L and *trn*H-*psb*A intergenic spacer regions, for authentication of the plant origin of raw materials and herbal products. Checking sequences from samples obtained from local markets and the Thai Food and Drug Administration (Thai FDA) against our digital reference DNA barcode system revealed the authenticity of eighteen out of twenty tested samples as claimed on their labels. Two samples, no. 3 and 13, were not *Cyanthillium cinereum* (L.) H.Rob. and *Pueraria candollei* Wall. ex Benth. as claimed, respectively. They were recognized as *Emilia sonchifolia* (L.) DC. and *Butea superba* (Roxb.), respectively. Hence, it is important for the Thai FDA or regulatory agencies to immediately initiate strict enforcement for the development of pharmacopoeial standards as well as revisions or modifications of available regulatory guidelines and to implement close monitoring for the quality control of herbal products in terms of authentication before they enter the herbal market. The centralized digital reference DNA barcode database developed here could play a very important role in monitoring or checking the authenticity of medicinal plants.

## Introduction

Traditional herbal medicine has long been practiced in health care systems in many countries worldwide. The global trade of herbal remedies and supplements is estimated to increase every year and is expected to reach approximately USD$ 117.02 billion by 2024^1^. The usage of herbal products has gained significant momentum in the recent past and is expected to continue to increase in the near future. In Thailand, traditional Thai medicine (TTM) was the most conventional healthcare system until the establishment of modern health care^2,3^. Consequently, as a result of many social and economic status changes, the use of TTM became limited to indigenous Thai people. However, the government has been trying to rejuvenate TTM to benefit the Thai medical system, especially in rural areas^4,5^. The quality parameters of herbal products are generally documented in the Thai Herbal Pharmacopoeia (THP) and Monographs of Selected Thai Materia Medica (TMM), the two reference textbooks endorsed by the Thai government.

The THP, currently in its 2021 edition^6^, was first established in 1989 by the Bureau of Drug and Narcotics, Department of Medical Sciences, Ministry of Public Health, to set forth quality standards for plants or herb-based drugs and herbal product preparations marketed in Thailand to ensure their identity, quality, safety, and efficacy. Intentional or unintentional adulteration of herbs leads to lower efficacy and affects herbal trade^7–10^. The common traditional authentication process of herbal products includes methods of botanical identification such as plant taxonomy, microscopic and macroscopic examination, and advanced chemical methods^11^. However, each method has advantages and limitations. The most frequent approaches are macroscopic and microscopic identification, which are fast and cost‐effective qualitative techniques. However, macroscopic analysis requires the whole plant, and it is difficult to apply to forms where plant morphology cannot be determined, for instance, mixtures of multiple herbs or extracted samples^12^. Phytochemical approaches or metabolomics profiling has been used for the identification of botanical drugs, dietary or food supplements and plant extracts^13^. Generally, phytochemical authentication depends on the selection of chemical markers that are unique to the selected plant species and is not always successful due to variation in geographical location and environmental conditions, including soil type, plant age, plant part, processing, storage conditions and other factors^8^. In addition, phytochemical analysis requires more reference samples from multiple populations to account for natural variability^14^. Among all recently developed methods, DNA-based methods are well-established for identifying plants in mixtures of herbal medicine products^15,16^.

A precise assessment is of foremost significance for purchasers, customers, patients and researchers along herbal product value chains^17^ including collectors, processors, harvesters, producers, regulators, traders, distributors, retailers, and traditional and medical practitioners^18^. Today, the armamentarium of prescription treatment is communicated through ‘pharmacopoeias’, which are standard collections of information on the quality of pharmaceutical drugs, excipients and flavoring correctives. The pharmacopoeia includes information on testing methodologies, purity, storage guidelines, composition and concentration for drugs. Pharmacopoeias ensure the consistency of cures endorsed by delegates of a particular unit and outline required quality principles. However, the regulatory affairs or policies for natural herbal products differ among nations. In some countries, for example, Canada, the United States and countries in the European Union (EU), governing regulatory agencies assess the quality and safety of herbal drugs/medicines before they enter the herbal market, but in practice, activities to control the authenticity and quality of herbal products in the herbal market appear to be limited^19^. The European Medicines Agency (EMA) updates the European Pharmacopoeias, including the monographs and testing methods in their database^20^, and the databases provide the most recent monographs and suitable methods for quality estimations of particular herbal drug products^21,22^. With accurate and rapid DNA-based techniques, DNA barcoding is now officially recognized as a method for identifying herbal drugs method^23^. DNA barcoding for quality control of herbal drugs is included in the British Pharmacopoeia (BP)^22,23^, Pharmacopoeia of the People’s Republic of China^24^ and Korean Pharmacopeia^25^, which includes plant sampling, DNA isolation, PCR amplification and development of standard reference sequence databases^8^.

Herein, we aimed to develop a digital reference DNA barcode library of plants listed in the THP and TMM using the nuclear and chloroplast DNA regions and to test for species adulteration in selected herbal products obtained from local markets and the Thai FDA. The centralized digital DNA barcode database developed here will also aid in the identification of any botanicals or herbal products in registration or regulatory processes.

## Results

### DNA barcoding of selected plants in the THP and TMM

Genomic DNA was successfully extracted from all 101 plant species belonging to 89 genera and 51 families (Table S1). The core DNA barcode regions, namely, the ITS2, *mat*K, *rbc*L and *trn*H*-psb*A intergenic spacer regions, were amplified. In the PCRs, positive and negative control amplifications gave accurate results. All PCR amplicons were clearly segregated and visible as single bands of the expected size. The partial sequence lengths ranged between 228 and 278 bp (average 258) for ITS2, 424 and 478 bp (average 450) for *mat*K, 540 and 580 bp (average 550) for *rbc*L and 420 and 458 bp (average 428) for the *trn*H*-psb*A intergenic spacer. All nucleotide sequences were submitted to NCBI GenBank, and their accession numbers are listed in Table 1.

**Table 1:** List of medicinal plants used in this study and their detailed information.

No	Botanical name	Family name	Monograph		Voucher number/ID	Collection location	GenBank accession numbers			
			TMM (volume)	THP (year)			ITS2	*mat*K	*rbc*L	*trn*H-*psb*A
1	*Syzygium aromaticum* (L.) Merr. & L.M. Perry	Myrtaceae	I	–	FPSCU SS-043	FPSCU	LC435390	LC435391	LC435392	LC435393
2	*Cinnamomum camphora* (L.) J.Presl	Lauraceae	I	–	FPSCU SS-044	FPSCU	LC435394	LC435395	LC435396	LC435397
3	*Strychnos nux-vomica* L	Strychnaceae	I	–	FPSCU SS-109	FPSCU	LC461741	LC461742	LC461743	LC461744
4	*Terminalia chebula* Retz	Combretaceae	I	2021	FPSCU SS-017	FPSCU	LC435434	LC435435	LC435436	LC435437
5	*Curcuma longa* L	Zingiberaceae	I	2021	FPSCU SS-002	FPSCU	LC461717	LC461718	LC461719	LC461720
6	*Zingiber officinale* Rosc	Zingiberaceae	I	2021	FPSCU SS-033	FPSCU	LC461745	LC461746	LC461747	LC461748
7	*Cassia fistula* L	Fabaceae	I	2021	FPSCU SS-051	FPSCU	LC435398	LC435399	LC435400	LC435401
8	*Santalum album* L	Santalaceae	I	2021	FPSCU SS-025	QSBG	LC435402	LC435403	LC435404	LC435405
9	*Pterocarpus santalinus* L.f	Fabaceae	I	2021	FPSCU SS-026	Bangkok	LC461725	LC461726	LC461727	LC461728
10	*Plumbago zeylanica* L	Plumbaginaceae	I	–	FPSCU SS-054	Bangkok	LC435406	LC435407	LC435408	LC435409
11	*Plumbago indica* L	Plumbaginaceae	I	–	FPSCU SS-055	FPSCU	LC435410	LC435411	LC435412	LC435413
12	*Tinospora baenzingeri* Forman	Menispermaceae	I	–	FPSCU SS-059	HPMSHG	LC435414	LC435415	LC435416	LC435417
13	*Senna alata* (L.) Roxb	Fabaceae	I	–	FPSCU SS-003	FPSCU	LC435422	LC435423	LC435424	LC435425
14	*Cymbopogon citratus* (DC.) Stapf	Poaceae	I	–	FPSCU SS-063	HPMSHG	LC461749	LC461750	LC461751	LC461752
15	*Solori scandens* (Roxb.) Benth	Fabaceae	I	2021	FPSCU SS-034	FPSCU	LC435418	LC435419	LC435420	LC435421
16	*Tinospora crispa* (L.) Miers ex Hook.f. & Thomson	Menispermaceae	I	2021	FPSCU SS-004	FPSCU	LC435426	LC435427	LC435428	LC435429
17	*Centella asiatica* (L.) Urb	Apiaceae	I	2021	FPSCU SS-012	FPSCU	LC461753	LC461754	LC461755	LC461756
18	*Vetiveria zizanioides* (L.) Nash ex Small	Poaceae	I	–	FPSCU SS-110	FPSCU	LC461921	LC461922	LC461923	LC461924
19	*Piper nigrum* L	Piperaceae	I	2021	FPSCU SS-005	FPSCU	LC461757	LC461758	LC461759	LC461760
20	*Andrographis paniculata* (Burm. f.) Wall. ex Nees	Acanthaceae	I	2021	FPSCU SS-007	FPSCU	LC461761	LC461762	LC461763	LC461764
21	*Phyllanthus emblica* L	Euphorbiaceae	I	2021	FPSCU SS-015	FPSCU	LC435430	LC435431	LC435432	LC435433
22	*Dracaena cochinchinensis* (Lour.) S.C.Chen	Dracaenaceae	I	2021	FPSCU SS-032	FPSCU	LC461765	LC461766	LC461767	LC461768
23	*Terminalia bellirica* (Gaertn.) Roxb	Combretaceae	I	2021	FPSCU SS-018	FPSCU	LC438866	LC438867	LC438868	LC438869
24	*Azadirachta indica* A.Juss	Meliaceae	I	–	FPSCU SS-093	HPMSHG	LC461769	LC461770	LC461771	LC461772
25	*Cyperus rotundus* L	Cyperaceae	I	–	FPSCU SS-149	Bangkok	LC461773	LC461774	LC461775	LC461776
26	*Boesenbergia rotunda* (L.) Mansf	Zingiberaceae	II	–	FPSCU SS-037	FPSCU	LC461777	LC461778	LC461779	LC461780
27	*Ocimum tenuiflorum* L	Lamiaceae	II	2021	FPSCU SS-001	HPMSHG	LC461781	LC461782	LC461783	LC461784
28	*Pluchea indica* (L.) Less	Asteraceae	II	–	FPSCU SS-047	FPSCU	LC438882	LC438883	LC438884	LC438885
29	*Alpinia galanga* (L.) Willd	Zingiberaceae	II	–	FPSCU SS-112	FPSCU	LC461785	LC461786	LC461787	LC461788
30	*Senna siamea* (Lam.) H.S.Irwin & Barneby	Fabaceae	II	–	FPSCU SS-010	FPSCU	LC438886	LC438887	LC438888	LC438889
31	*Aristolochia pierrei* Lec	Aristolochiaceae	II	2021	MUS-5409	Sakon Nakhon	KP998796	KP998782	KP998768	KP998810
32	*Capparis micracantha* DC	Capparaceae	II	–	FPSCU SS-060	HPMSHG	LC438890	LC438891	LC438892	LC438893
33	*Myristica fragrans* Houtt	Myristicaceae	II	2021	FPSCU SS-052	HPMSHG	LC461925	LC461926	LC461927	LC461928
34	*Piper retrofractum* Vahl	Piperaceae	II	2021	FPSCU SS-011	FPSCU	LC461929	LC461930	LC461931	LC461932
35	*Nelumbo nucifera* Gaertn	Nelumbonaceae	II	2021	FPSCU SS-027	Bangkok	LC438878	LC438879	LC438880	LC438881
36	*Mesua ferrea* L	Calophyllaceae	II	2021	FPSCU SS-028	FPSCU	LC461789	LC461790	LC461791	LC461792
37	*Kaempferia galanga* L	Zingiberaceae	II	–	FPSCU SS-068	HPMSHG	LC461793	LC461794	LC461795	LC461796
38	*Mimusops elengi* L	Sapotaceae	II	2021	FPSCU SS-029	FPSCU	LC438870	LC438871	LC438872	LC438873
39	*Citrus hystrix* DC	Rutaceae	II	2021	FPSCU SS-014	FPSCU	LC438898	LC438899	LC438900	LC438901
40	*Tamarindus indica* L	Fabaceae	II	–	FPSCU SS-076	HPMSHG	LC461733	LC461734	LC461735	LC461736
41	*Ficus racemosa* L	Moraceae	II	2021	FPSCU SS-077	HPMSHG	LC461797	LC461798	LC461799	LC461800
42	*Aegle marmelos* (L.) Correa	Rutaceae	II	2021	FPSCU SS-078	HPMSHG	LC461801	LC461802	LC461803	LC461804
43	*Jasminum sambac* (L.) Sol	Oleaceae	II	–	FPSCU SS-114	FPSCU	LC461805	LC461806	LC461807	LC461808
44	*Clerodendrum indicum* (L.) Kuntze	Lamiaceae	II	2021	FPSCU SS-082	HPMSHG	LC461737	LC461738	LC461739	LC461740
45	*Tiliacora triandra* Diels	Menispermaceae	II	2021	FPSCU SS-083	HPMSHG	LC438894	LC438895	LC438896	LC438897
46	*Brucea javanica* (L.) Merr	Simaroubaceae	II	–	FPSCU SS-086	HPMSHG	LC438902	LC438903	LC438904	LC438905
47	*Acacia concinna* (Willd.) DC	Fabaceae	II	–	FPSCU SS-115	FPSCU	LC461933	LC461934	LC461935	LC461936
48	*Mammea siamensis* (T. Anderson) Kosterm	Calophyllaceae	II	–	FPSCU SS-095	HPMSHG	LC438874	LC438875	LC438876	LC438877
49	*Hibiscus sabdariffa* L	Malvaceae	III	2021	FPSCU SS-036	FPSCU	LC461809	LC461810	LC461811	LC461812
50	*Cananga odorata* (Lam.) Hook. f. et Thomson var. Odorata	Annonaceae	III	–	FPSCU SS-038	FPSCU	LC438906	LC438907	LC438908	LC438909
51	*Alocasia macrorrhizos* (L.) G. Don	Araceae	III	–	FPSCU SS-116	FPSCU	LC461937	LC461938	LC461939	LC461940
52	*Euphorbia antiquorum* L	Euphorbiaceae	III	–	FPSCU SS-042	FPSCU	LC438910	LC438911	LC438912	LC438913
53	*Artocarpus heterophyllus* Lam	Moraceae	III	–	FPSCU SS-120	FPSCU	LC461813	LC461814	LC461815	LC461816
54	*Aquilaria crassna* Pierre ex Lecomte	Thymelaeaceae	III	–	FPSCU SS-040	FPSCU	LC461817	LC461818	LC461819	LC461820
55	*Pueraria candollei* Wall. ex Benth. var. mirifica (Airy Shaw et Suvat.) Niyomdham	Fabaceae	III	–	FPSCU SS-041	FPSCU	LC456342	LC456343	LC456344	LC456345
56	*Eclipta prostrata* (L.) L	Asteraceae	III	–	FPSCU SS-117	FPSCU	LC461821	LC461822	LC461823	LC461824
57	*Streblus asper* Lour	Moraceae	III	–	FPSCU SS-048	FPSCU	LC456346	LC456347	LC456348	LC456349
58	*Bixa orellana* L	Bixaceae	III	–	FPSCU SS-121	FPSCU	LC461941	LC461942	LC461943	LC461944
59	*Magnolia champaca* (L.) Baillon ex Pierre var. champaca	Magnoliaceae	III	–	FPSCU SS-053	Bangkok	LC461825	LC461826	LC461827	LC461828
60	*Elephantopus scaber* L	Asteraceae	III	–	FPSCU SS-062	HPMSHG	LC456350	LC456351	LC456352	LC456353
61	*Rhinacanthus nasutus* (L.) Kurz	Acanthaceae	III	–	FPSCU SS-065	HPMSHG	LC461829	LC461830	LC461831	LC461832
62	*Jatropha multifida* L	Euphorbiaceae	III	–	FPSCU SS-071	HPMSHG	LC461833	LC461834	LC461835	LC461836
63	*Clinacanthus nutans* (Burm.f.) Lindau	Acanthaceae	III	2021	FPSCU SS-023	QSBG	LC456354	LC456355	LC456356	LC456357
64	*Piper betle* L	Piperaceae	III	2021	FPSCU SS-013	FPSCU	LC461837	LC461838	LC461839	LC461840
65	*Houttuynia cordata* Thunb	Saururaceae	III	–	FPSCU SS-072	HPMSHG	LC456358	LC456359	LC456360	LC456361
66	*Oroxylum indicum* (L.) Benth. ex Kurz	Bignoniaceae	III	–	FPSCU SS-074	HPMSHG	LC456362	LC456363	LC456364	LC456365
67	*Cissus quadrangularis* L	Vitaceae	III	2021	FPSCU SS-030	FPSCU	LC456366	LC456367	LC456368	LC456369
68	*Moringa oleifera* Lam	Moringaceae	III	2021	FPSCU SS-125	FPSCU	LC461949	LC461950	LC461951	LC461952
69	*Solanum trilobatum* L	Solanaceae	III	2021	FPSCU SS-008	FPSCU	LC461841	LC461842	LC461843	LC461844
70	*Garcinia mangostana* L	Clusiaceae	III	–	FPSCU SS-080	HPMSHG	LC461845	LC461846	LC461847	LC461848
71	*Thunbergia laurifolia* Lindl	Acanthaceae	III	2021	FPSCU SS-031	FPSCU	LC456370	LC456371	LC456372	LC456373
72	*Acorus calamus* L	Acoraceae	III	2021	FPSCU SS-016	FPSCU	LC461849	LC461850	LC461851	LC461852
73	*Lagerstroemia speciosa* (L.) Pers	Lythraceae	III	–	FPSCU SS-107	Bangkok	LC461853	LC461854	LC461855	LC461856
74	*Salacia chinensis* L	Celastraceae	IV	–	FPSCU SS-127	FPSCU	LC461857	LC461858	LC461859	LC461860
75	*Arcangelisia flava* (L.) Merr	Menispermaceae	IV	2021	FPSCU SS-020	FPSCU	LC461721	LC461722	LC461723	LC461724
76	*Alyxia reinwardtii* Blume	Apocynaceae	IV	–	FPSCU SS-056	HPMSHG	LC461729	LC461730	LC461731	LC461732
77	*Piper sarmentosum* Roxb	Piperaceae	IV	2021	FPSCU SS-021	FPSCU	LC461861	LC461862	LC461863	LC461864
78	*Cryptolepis dubia* (Burm.f.) M.R.Almeida	Apocynaceae	IV	–	FPSCU SS-064	HPMSHG	LC456374	LC456375	LC456376	LC456377
79	*Annona squamosa* L	Annonaceae	IV	–	FPSCU SS-066	HPMSHG	LC461865	LC461866	LC461867	LC461868
80	*Caesalpinia sappan* L	Fabaceae	IV	–	FPSCU SS-070	HPMSHG	LC456378	LC456379	LC456380	LC456381
81	*Bridelia ovata* Decne	Phyllanthaceae	IV	–	FPSCU SS-075	HPMSHG	LC456382	LC456383	LC456384	LC456385
82	*Citrus aurantifolia* (Christm.) Swingle	Rutaceae	IV	–	FPSCU SS-079	HPMSHG	LC456386	LC456387	LC456388	LC456389
83	*Garcinia hanburyi* Hook. f	Clusiaceae	IV	–	FPSCU SS-084	HPMSHG	LC456406	LC456407	LC456408	LC456409
84	*Aloe vera* (L.) Burm.f	Asphodelaceae	IV	–	FPSCU SS-128	FPSCU	LC461869	LC461870	LC461871	LC461872
85	*Terminalia citrina* (Gaertn.) Roxb. ex Fleming	Combretaceae	IV	–	FPSCU SS-091	HPMSHG	LC461873	LC461874	LC461875	LC461876
86	*Citrus maxima* (Burm.) Merr	Rutaceae	IV	–	FPSCU SS-129	FPSCU	LC461877	LC461878	LC461879	LC461880
87	*Caesalpinia bonduc* (L.) Roxb	Fabaceae	IV	2021	FPSCU SS-009	FPSCU	LC461881	LC461882	LC461883	LC461884
88	*Tectona grandis* L.f	Lamiaceae	IV	–	FPSCU SS-094	HPMSHG	LC461885	LC461886	LC461887	LC461888
89	*Cyanthillium cinereum* (L.) H.Rob	Asteraceae	IV	2021	SS-645	FPSCU	LC503563	LC503564	LC503565	LC503566
90	*Orthosiphon aristatus* (Blume) Miq	Lamiaceae	IV	2021	FPSCU SS-024	QSBG	LC456390	LC456391	LC456392	LC456393
91	*Syzygium cumini* (L.) Skeels	Myrtaceae	IV	–	FPSCU SS-131	FPSCU	LC461889	LC461890	LC461891	LC461892
92	*Kaempferia parviflora* Wall. ex Baker	Zingiberaceae	V	2021	FPSCU SS-019	FPSCU	LC461893	LC461894	LC461895	LC461896
93	*Ziziphus attopensis* Pierre	Rhamnaceae	V	–	FPSCU SS-045	FPSCU	LC461897	LC461898	LC461899	LC461900
94	*Bacopa monnieri* (L.) Wettst	Plantaginaceae	V	–	CU-MN 20170126	FPSCU	LC214982	LC214984	LC214987	LC214981
95	*Morus alba* L	Moraceae	V	2021	FPSCU SS-101	HPMSHG	LC461901	LC461902	LC461903	LC461904
96	*Sapindus rarak* DC	Sapindaceae	V	–	FPSCU SS-142	FPSCU	LC461905	LC461906	LC461907	LC461908
97	*Wrightia arborea* (Dennst.) Mabb	Apocynaceae	V	–	FPSCU SS-081	HPMSHG	LC456394	LC456395	LC456396	LC456397
98	*Blumea balsamifera* (L.) DC	Asteraceae	V	–	FPSCU SS-139	FPSCU	LC461909	LC461910	LC461911	LC461912
99	*Imperata cylindrica* (L.) Raeusch	Poaceae	V	–	FPSCU SS-096	HPMSHG	LC461913	LC461914	LC461915	LC461916
100	*Ventilago denticulata* Willd	Rhamnaceae	V	–	FPSCU SS-085	HPMSHG	LC461917	LC461918	LC461919	LC461920
101	*Momordica charantia* L	Cucurbitaceae	-	2021	FPSCU SS-124	FPSCU	LC461945	LC461946	LC461947	LC461948

*THP* Thai Herbal Pharmacopoeia, *TMM* Thai Materia Medica, *FPSCU* Faculty of Pharmaceutical Sciences, Chulalongkorn University, Bangkok, *HPMSHG* HRH Princess Mahachakri Sirindhorn Herbal Garden, Rayong, *QSBG* Queen Sirikit Botanical Garden, Chiang Mai.

### Authentication of herbal products

**Figure 1:**
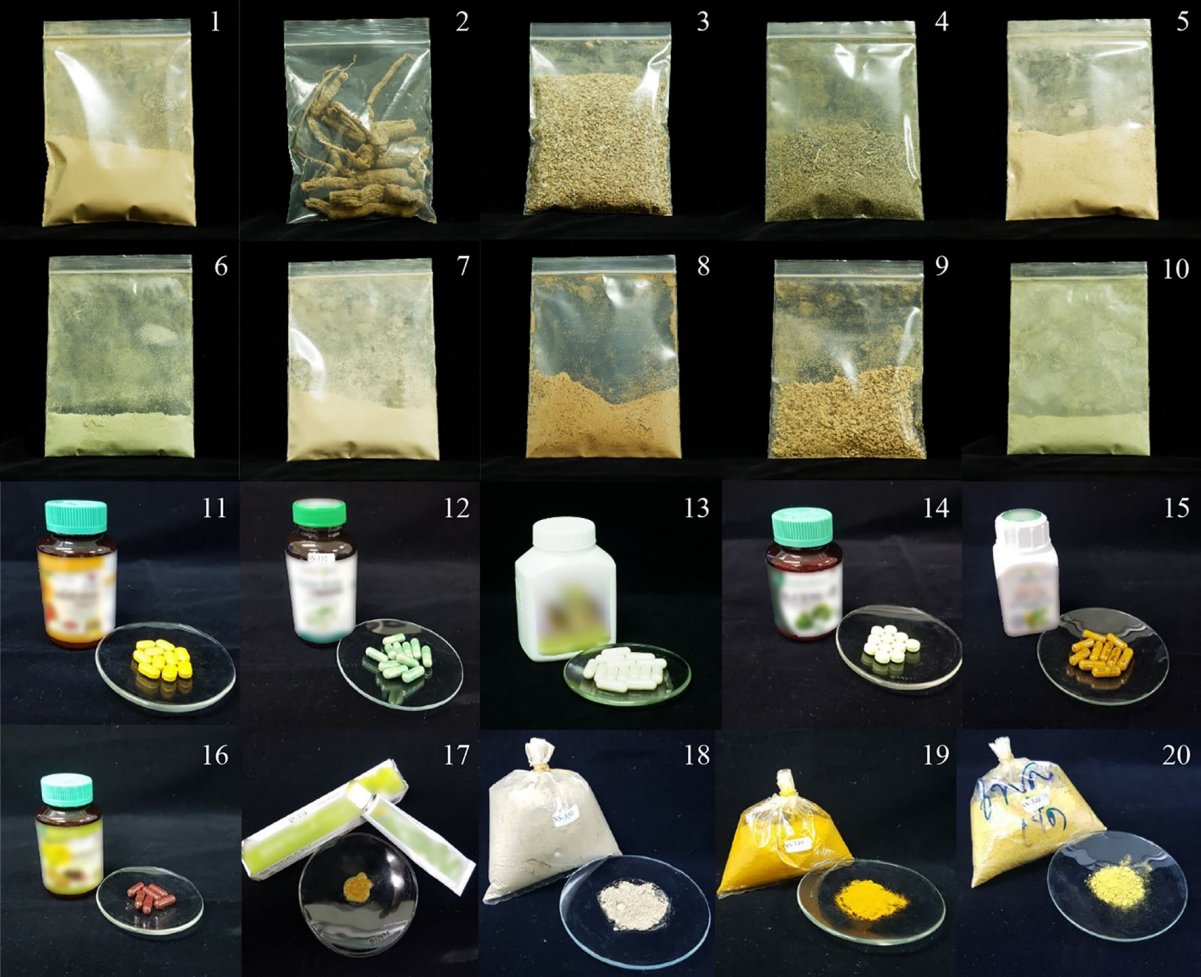
Different dosage forms of herbal products analyzed in this study.

Genomic DNA was successfully isolated from all twenty different dosage forms of herbal products (Fig. 1; Table S2) and amplified for four barcode regions, namely, the ITS2, *mat*K, *rbc*L and *trn*H*-psb*A intergenic spacer regions. Furthermore, the authenticity of all twenty samples of single-herb formulation products was tested using our reference DNA barcode database and nucleotide Basic Local Alignment Search Tool (BLAST) analysis of available NCBI GenBank sequences (Table S3). The results confirmed the authenticity of eighteen out of the twenty samples tested. The sequences obtained from the other two samples, no. 3 and 13, which were purchased from local markets, did not match the name on their labels (Table 2). Sample no. 3 was labeled as *Cyanthillium cinereum* and sample no. 13 was labeled as *Pueraria candollei*. However, our nucleotide BLAST results showed that sample no. 3 and 13 were *Emilia sonchifolia* and *Butea superba*, respectively. All samples provided by the Thai FDA were correct according to their claims. The NCBI GenBank nucleotide blast results of these samples are provided in Table 2.

**Table 2:** Nucleotide sequence BLAST results of herbal products.

Sample code	Corresponding scientific names as per their label claim	Dosage form	NCBI BLAST result				Species identified using our reference DNA barcode library
			ITS2	*mat*K	*rbc*L	*trn*H-*psb*A	
1	*Bacopa monnieri*	Powder	*Bacopa monnieri*	*Bacopa monnieri*	*Bacopa monnieri*	*Bacopa monnieri*	*Bacopa monnieri*
2	*Aristolochia pierrei*	Crude drug	*Aristolochia pierrei*	*Aristolochia pierrei*	*Aristolochia pierrei*	*Aristolochia pierrei*	*Aristolochia pierrei*
3	*Cyanthillium cinereum*	Powder	*Emilia sonchifolia*	*Emilia sonchifolia*	*Emilia sonchifolia*	*Emilia sonchifolia*	*Emilia sonchifolia*
4	*Thunbergia laurifolia*	Powder	*Thunbergia laurifolia*	*Thunbergia laurifolia*	*Thunbergia laurifolia*	*Thunbergia laurifolia*	*Thunbergia laurifolia*
5	*Phyllanthus emblica*	Powder	*Phyllanthus emblica*	*Phyllanthus emblica*	*Phyllanthus emblica*	*Phyllanthus emblica*	*Phyllanthus emblica*
6	*Andrographis paniculata*	Powder	*Andrographis paniculata*	*Andrographis paniculata*	*Andrographis paniculata*	*Andrographis paniculata*	*Andrographis paniculata*
7	*Pueraria candollei* var. *mirifica*	Powder	*Pueraria candollei*	*Pueraria candollei*	*Pueraria candollei*	*Pueraria candollei*	*Pueraria candollei*
8	*Senna alata*	Powder	*Senna alata*	*Senna alata*	*Senna alata*	*Senna alata*	*Senna alata*
9	*Boesenbergia rotunda*	Powder	*Boesenbergia rotunda*	*Boesenbergia rotunda*	*Boesenbergia rotunda*	*Boesenbergia rotunda*	*Boesenbergia rotunda*
10	*Clinacanthus nutans*	Powder	*Clinacanthus nutans*	*Clinacanthus nutans*	*Clinacanthus nutans*	*Clinacanthus nutans*	*Clinacanthus nutans*
11	*Curcuma longa*	Tablet	*Curcuma longa*	*Curcuma sp.*	*Curcuma sp.*	*Curcuma longa*	*Curcuma longa*
12	*Centella asiatica*	Capsule	*Centella asiatica*	*Centella asiatica*	*Centella asiatica*	*Centella asiatica*	*Centella asiatica*
13	*Pueraria candollei*	Capsule	*Butea superba*	*Butea superba*	Butea superba*	*Butea superba*	*Butea superba*
14	*Centella asiatica*	Tablet	*Centella asiatica*	*Centella asiatica*	*Centella asiatica*	*Centella asiatica*	*Centella asiatica*
15	*Curcuma longa*	Capsule	*Curcuma longa*	*Curcuma sp.*	*Curcuma sp.*	*Curcuma longa*	*Curcuma longa*
16	*Kaempferia parviflora*	Capsule	*Kaempferia parviflora*	*Kaempferia sp.*	*Kaempferia sp.*	*Kaempferia parviflora*	*Kaempferia parviflora*
17	*Centella asiatica*	Cream	*Centella asiatica*	*Centella asiatica*	*Centella asiatica*	*Centella asiatica*	*Centella asiatica*
18	*Centella asiatica*	Powder	*Centella asiatica*	*Centella asiatica*	*Centella asiatica*	*Centella asiatica*	*Centella asiatica*
19	*Curcuma longa*	Powder	*Curcuma longa*	*Curcuma longa*	*Curcuma sp.*	*Curcuma longa*	*Curcuma longa*
20	*Zingiber montanum*	Powder	*Zingiber montanum*	*Zingiber montanum*	*Zingiber montanum*	*Zingiber montanum*	*Zingiber montanum*

*Indicates that the Barcode of Life Data System (BOLD) database was used for sample analysis.

### Maximum likelihood phylogenetic analysis

**Figure 2:**
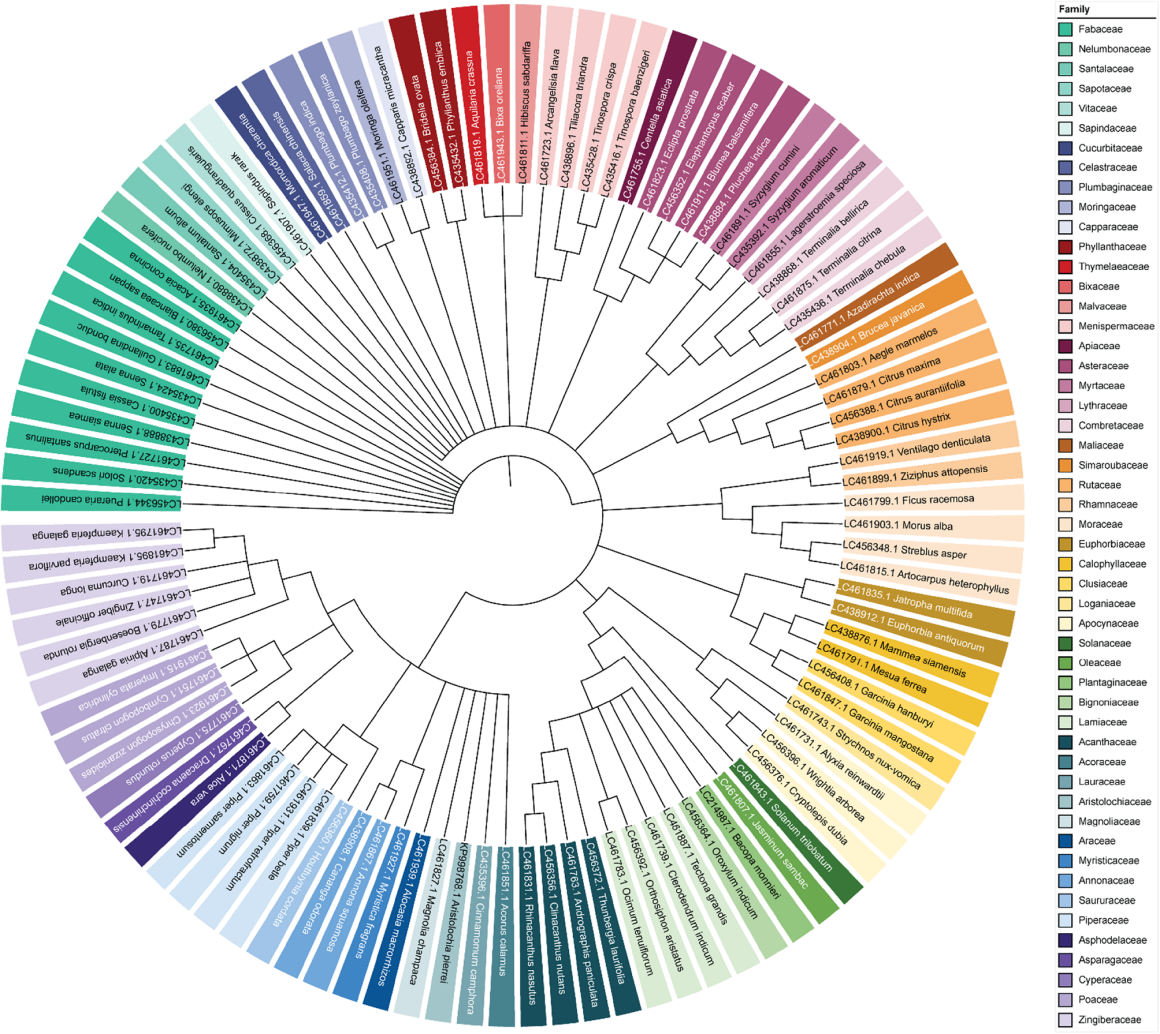
Maximum likelihood tree showing the phylogenetic relationships of reference Thai medicinal plants based on the Kimura-2-parameter (K2P) model using the *rbc*L region. The bootstrap support values were estimated with 1000 replicates. The respective family names are shown to the right.

Maximum likelihood (ML) phylogenetic analysis of all reference plant species was performed using the ITS2, *mat*K, *rbc*L, and *psb*A-*trn*H regions. The unrooted phylogenetic tree of the *rbc*L region showed clear clades, and each cluster represented a specific group of plant species (Fig. 2). Each color represents a monophyletic clade based on plant genera and families, indicating their close phylogenetic relationships. A large number of plant species clusters belonged to the Asteraceae, Fabaceae, Lamiaceae, Rutaceae, and Zingiberaceae families. The bootstrap values were estimated with 1000 replicates with support values. These findings showed that the *rbc*L region-based phylogenetic tree can be used as an efficient resource for species authentication of Thai medicinal plants. Our unrooted ML phylogenetic tree of reference species mirrored the taxonomic classification of Thai plants listed in the THP and TMM (Fig. 2).

### Development of a centralized reference DNA barcode database

**Figure 3:**
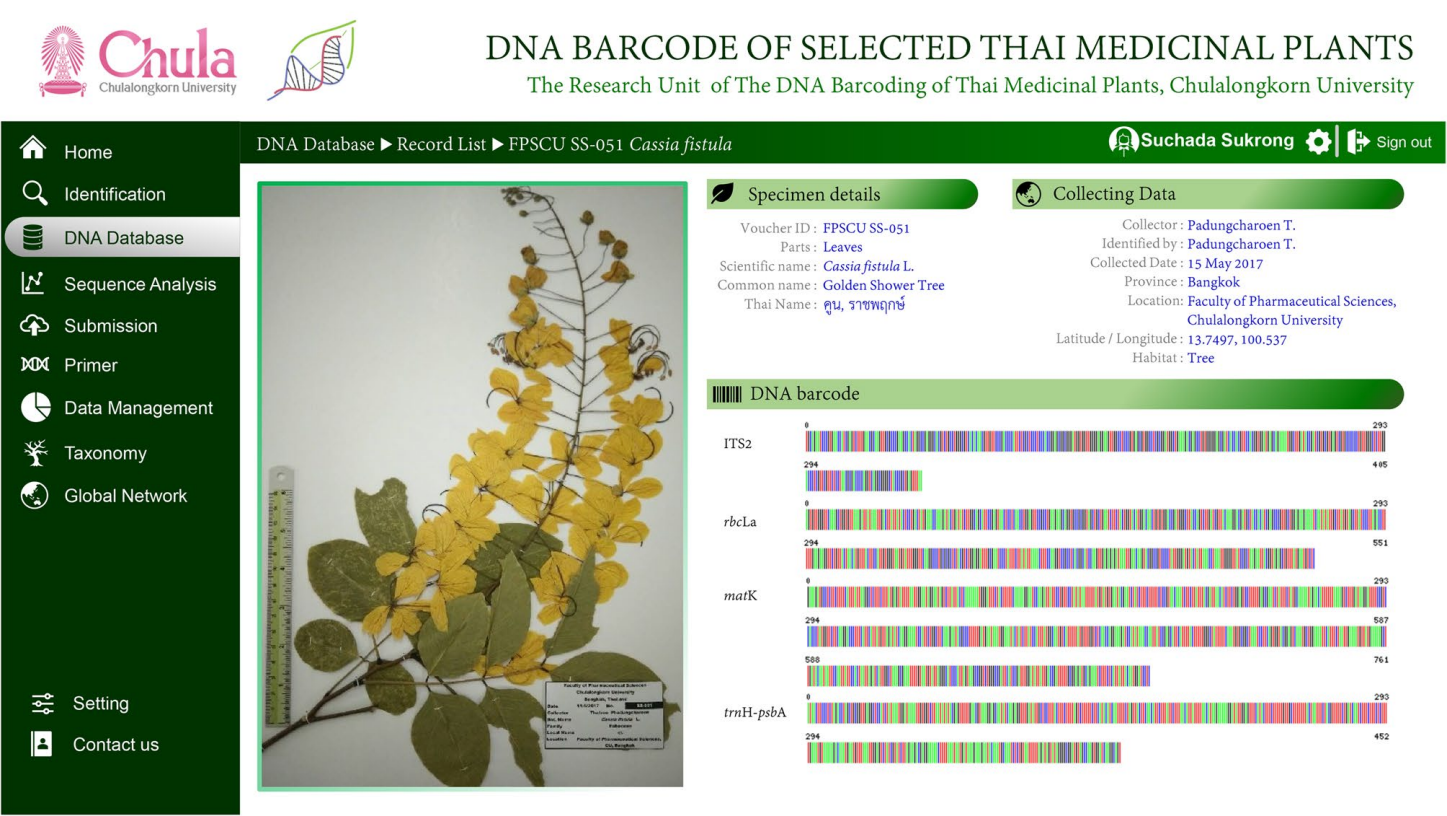
Overview of the proposed digital reference DNA barcode database.

In this study, a centralized digital reference DNA barcode system for regulating herbal products was developed. The reference DNA barcode database incorporates voucher numbers, scientific names, common names, Thai names, plant habitats, collection forms, plant photographs, herbarium images and other information, such as collection dates, collection locations, collectors, and taxonomists, along with geocoordinates (Fig. 3). All DNA barcode marker information, including genes, gene sequences, and GenBank accession numbers, will be included in the database. Using the scientific name or Thai name in the search option, the end user can obtain all the information for a particular plant. An attempt to establish a digital database system is made, and the database is found to be an efficient tool with which to systematically assess traditional medicine and its herbal products and connect it with both national and international herbal trade regulators. This database system is a novel concept in Thai herbal development, and its availability to the industry as well as consumers and researchers will bring a noticeable change in the regulation of herbal trade.

## Discussion

The global markets of herbal drugs are large and increasing every year. However, increasing demand leads to adulteration or substitution in the raw materials^10,26,27^. Many reports of adverse reactions may often be due to the consumption of unintended herbs, which has directly affected the marketing or campaign of herbal products^9,10,12,16,27^. Various identification methods, including taxonomic, genomic, and phytochemistry methods, have been used to authenticate herbal products^28^. However, each method has advantages and limitations. Recently, DNA-based methods have been widely established for the authentication of herbal products^12,26,27^.

In this study, DNA barcodes of 101 highly traded medicinal plants listed in the THP and TMM of Thailand were developed. The highly traded samples of single-herb formulations that are not restricted to closely related plant species obtained from a local market and the Thai FDA were tested for their authenticity. Irrespective of the herbal samples, DNA analysis has been done using our own reference database along with available NCBI nucleotide blast analysis. Due to the inherent limitations of single-locus of DNA barcoding, an emerging DNA-based, or phylogenetic method is needed for the identification of closely related plant species. The utilization of DNA as a source of information for identifying inaccurate plant ingredients on herbal product labels is starting to be explored^9,10,16^. Four core DNA barcode regions, namely, the ITS2, *mat*K, *rbc*L, and *trn*H-*psb*A intergenic spacer regions, were used to develop a reference DNA barcode library for testing the authenticity of twenty single formulation herbal products. Our analysis indicated that all twenty samples tested for their authenticity were correct according to their labels, except samples no. 3 and no. 13, which were from powder and capsules labeled *Cyanthillium cinereum* and *Pueraria candollei*, respectively. Nucleotide BLAST results revealed that the *Cyanthillium cinereum* (sample no. 3) powder was replaced by *Emilia sonchifolia*, and the *Pueraria candollei* (sample no. 13) capsules contained instead *Butea superba.* Similar morphologies and confusion of vernacular names could explain this replacement. *Cyanthillium cinereum* has high antioxidant activity^29^ and is used in Thai medicine to reduce smoking withdrawal symptoms and treat skin ailments, as well as asthma, bronchitis, cough, cancer, malaria, gastrointestinal conditions, diuresis, pain, and diabetes^30,31^. *Emilia sonchifolia* is used for the treatment of anti-inflammatory stomach tumors, ophthalmia, diarrhea, wounds, intestinal worm infections and bleeding piles^32^. *Pueraria candollei* is used to relieve menopausal symptoms, including vasomotor symptoms, reproductive symptoms, depression, and musculoskeletal pain, in estrogen-deficient women^33^. *Butea superba* has been used for rejuvenation, for sexual arousal, and to prevent erectile dysfunction^34^. These results clearly indicate the extent of the problem that might occur due to the use of unauthentic raw drugs in Thai medicine. There were no *rbc*L reference sequences of sample no. 13 in NCBI GenBank; hence, the Barcode of Life Data System (BOLD) database was used to analyze this sample. Both of these samples were obtained from a local market. Herbal products purchased from local marketplaces could be more likely to obtain adulterations or admixtures, especially powder samples. It is very difficult to differentiate mixed powdered forms. Previously, many reports showed that the powdered form of samples had a greater chance of admixture than other forms, for example, the powdered form of ginger (*Zingiber officinale* Roscoe) admixed with chili powder (*Capsicum annuum* L.)^35^ and the powdered form of black pepper (*Piper nigrum* L.) admixed with chili powder (*Capsicum annuum* L.)^36^.

For the purpose of this study, an ML phylogenetic tree of our reference plant species was constructed using all four DNA barcode regions. Among the markers, *rbc*L is highly conserved, and its sequence query revealed the highest identity with plant species or closely related plant species. However, identification by this marker will not be reliable if the taxonomic identity of the nucleotide sequence in the GenBank database is incorrect. These issues can be resolved by using a phylogenetic tree wherein the incorrectly identified samples are highly likely to be located in unexpected clades^37^. Our *rbc*L region phylogenetic tree showed the arrangement of all the plant species in appropriate clades or plant groups, as would be expected based on phylogenetic relationships among the plant species (Fig. 2). Therefore, taxonomic identification using the *rbc*L region at the species level is more reliable than other regions tested in this study. These results were consistent with those of previous reports that the *rbc*L region is a suitable candidate region for plant species identification^38,39^. Previously, the utility of the *rbc*L region in discriminating land plants was successfully validated^40^. The use of *rbc*L has increased due to its high discrimination proportions at low taxonomic levels^39^. In this study, the *mat*K and *trn*H-*psb*A regions were unable to differentiate the plant species, and the ITS2 region showed similar results, with a few of the plants of the same genus clustered with different groups of plants (Fig. S1). Therefore, this study was restricted to the *rbc*L region-based ML phylogenetic tree; however, multilocus DNA barcode techniques could be used as advanced tools for the accurate identification of medicinal plants.

Numerous adulteration and substitution studies of herbal products have been reported worldwide, including in Thailand. In addition, the international herbal product supply chain repeatedly lacks botanical expertise to provide suitable documentation for the identification of raw herbal materials^37^. Unfortunately, in Thailand, there is no systematic regulatory mechanism for the quality control of herbal drugs before entering the market. It is very important to use appropriate analytical techniques for herbal products. Through this study, we propose a centralized digital DNA barcode database to aid in the regulatory step of identifying the plants used in herbal products. This reference database incorporates voucher numbers, scientific and common names, Thai names, plant habitats, collection forms, and plant photographs, including herbarium images, and other information such as collection dates, collection locations, and geocoordinates. By using scientific or common names, one can obtain all the information on a particular plant species or herbal product. This database could play a very important role in monitoring or checking medicinal plants or herbal trade and could ensure that all essential information is freely accessible to consumers and regulatory authorities in Thailand. Herbal testing centers and certification facilities will enhance the quality control of herbal products and help regulate the national and international herbal trade. Further, we are planning to extend the test to medicinal and non-medicinal plants available in Thailand. Future research will continue to validate and update the reference DNA barcode library and protocol or procedure for analyzing herbal samples. Furthermore, certification of the ingredients mentioned on herbal product labels using our reference DNA barcode database will continue.

## Conclusion

Admixture or adulteration in herbal products is one of the main problems in herbal trade because the identification of herbal ingredients is challenging. Hence, there is an important requirement to develop a reference DNA barcode library or centralized digital database system that could serve as a regulatory database for ensuring the safety and quality of traded herbs. It is very important that the Thai FDA immediately begin to strictly enforce the development of pharmacopeial standards as well as revisions or modifications of existing regulatory guidelines to check or monitor the authenticity of raw materials or herbal products before they enter the herbal market. For quality assessment of herbal products, we strongly recommend incorporating DNA-based methods into the THP and TMM to maintain the safety, quality and efficacy of herbal medicines prior to them entering the market.

## Materials and methods

### Plant materials and herbal products

Multiple accessions of plant species mentioned in the THP and TMM were collected from several locations in Thailand (Table 1). The procedures for plant collection and field studies were conducted by following standard guidelines of Chulalongkorn University, Thailand. Those collections including samples from Thai FDA are permitted and legal. A total of 101 plant species and their voucher numbers were prepared as herbarium specimens and deposited at the Museum of Natural Medicine, Chulalongkorn University, Bangkok, Thailand. All plant species were identified by an independent expert taxonomist, Associate Professor Thatree Phadungcharoen of the Faculty of Pharmaceutical Sciences, Chulalongkorn University. Details of the collection of plant species with their voucher numbers, respective Thai names and GenBank accession numbers are provided (Table 1). Their binomial names and author citations of the plant species were confirmed according to The Plant List (TPL)^41^. Seventeen single formulation herbal products from local herbal markets across Thailand and three herbal products from the Thai FDA were analyzed in this study. Herbal sample codes are listed in Table 2.

### DNA isolation and PCR amplification

Genomic DNA was isolated from leaves using a DNeasy Plant Mini Kit (Qiagen, Germany) according to the manufacturer’s protocol. Further PCR amplification was carried out in a 25 µL reaction volume that consisted of 1X PCR buffer, 1.5 mM MgCl_2,_ 0.2 mM dNTPs mix, 0.2 mM each forward and reverse primer, 0.5 U of Platinum *Taq* polymerase (Invitrogen, USA) and 30–40 ng of genomic DNA. Amplification was performed with an Eppendorf Master Cycler Gradient (Hamburg, Germany). PCR amplification with primers was carried out by using universal barcode regions^40^. the ITS2 nuclear region (ITS2F-ATTCCCGGACCACGCCTGGCTGA^42^; ITS4-TCCTCCGCTTATTGATATGC^43^) and three chloroplast regions: *mat*K (*mat*K_xF-TAATTTACGATCAATTCATTC^44^; *mat*K*-*MALPR1- ACAAGAAAGTCGAAGTAT^45^), the *trn*H*-psb*A intergenic spacer (*trn*Hf_05– CGCGCATGGTGGATTCACAATCC^46^; *psb*A3_f–GTTATGCATGAACGTAATGCTC^47^) and *rbc*L (*rbc*La-F-ATGTCACCACAAACAGAGACTAAAGC^48^; *rbc*La-R-GTAAAATCAAGTCCACCRCG^49^) were used. PCR amplification of the ITS and *psb*A-*trn*H intergenic spacer regions was performed at 95 °C for 4 min followed by 30 cycles of 94 °C for 45 s, 58 °C for 45 s, and 72 °C for 90 s, with a final extension at 72 °C for 7 min. The amplification profiles for *mat*K and *rbc*L consisted of 94 °C for 4 min followed by 30 cycles of initial denaturation at 94 °C for 60 s, 55 °C for 45 s, and 72 °C for 90 s, with a final extension step at 72 °C for 10 min. The obtained PCR amplicons were sequenced bidirectionally using their respective primers on an ABI3500 sequencer (Applied Biosystem, USA).

Genomic DNA of different dosage forms of the herbal product was extracted using a DNeasy Plant Mini Kit (Qiagen, Germany) and further purified using a GENECLEAN Kit (MP Biomedicals, France). The DNA isolation of herbal samples required multiple attempts to obtain good PCR amplification against the ITS2, *mat*K, *rbc*L and *psb*A-*trn*H intergenic spacer regions. Subsequently, all those PCR products were sequenced as described above.

### DNA sequencing and phylogenetic analysis

The sequences were edited using BioEdit software (version 5.0.6). BLAST analysis was conducted with the sequences as queries to determine the similarity of the nucleotide sequences in NCBI GenBank. The sequences with the maximum query coverage, highest homology, and maximum score were downloaded in FASTA format from the database and included in our analysis. The ML method was used to construct the relationships among plant samples with an appropriate model of nucleotide evolution. The final alignment file was imported into MEGA 7 to determine the character information prior to phylogenetic analysis using the Kimura 2-parameter molecular evolution model with 1,000 rapid bootstrapping replicates^50^.

## Supplementary Information

Supplementary Figure 1.

Supplementary Table 1.

Supplementary Table 2.

Supplementary Table 3.

## Data Availability

The datasets generated during and/or analyzed during the current study are available in the NCBI GenBank repository, accession no: LC214981, LC214982, LC214984, LC214987, LC435390-LC435437, LC438866-LC456409, LC461717-LC461952, LC503563-LC503566, KP998768, KP998782, KP998796, KP998810.
