# Supplementary figures and images for: Development of a DNA barcode library of plants in the Thai Herbal Pharmacopoeia and Monographs for authentication of herbal products
Source: Sci Rep. 2022 Jun 10;12:9624. doi: 10.1038/s41598-022-13287-x (PMC9187672; doi:10.1038/s41598-022-13287-x)

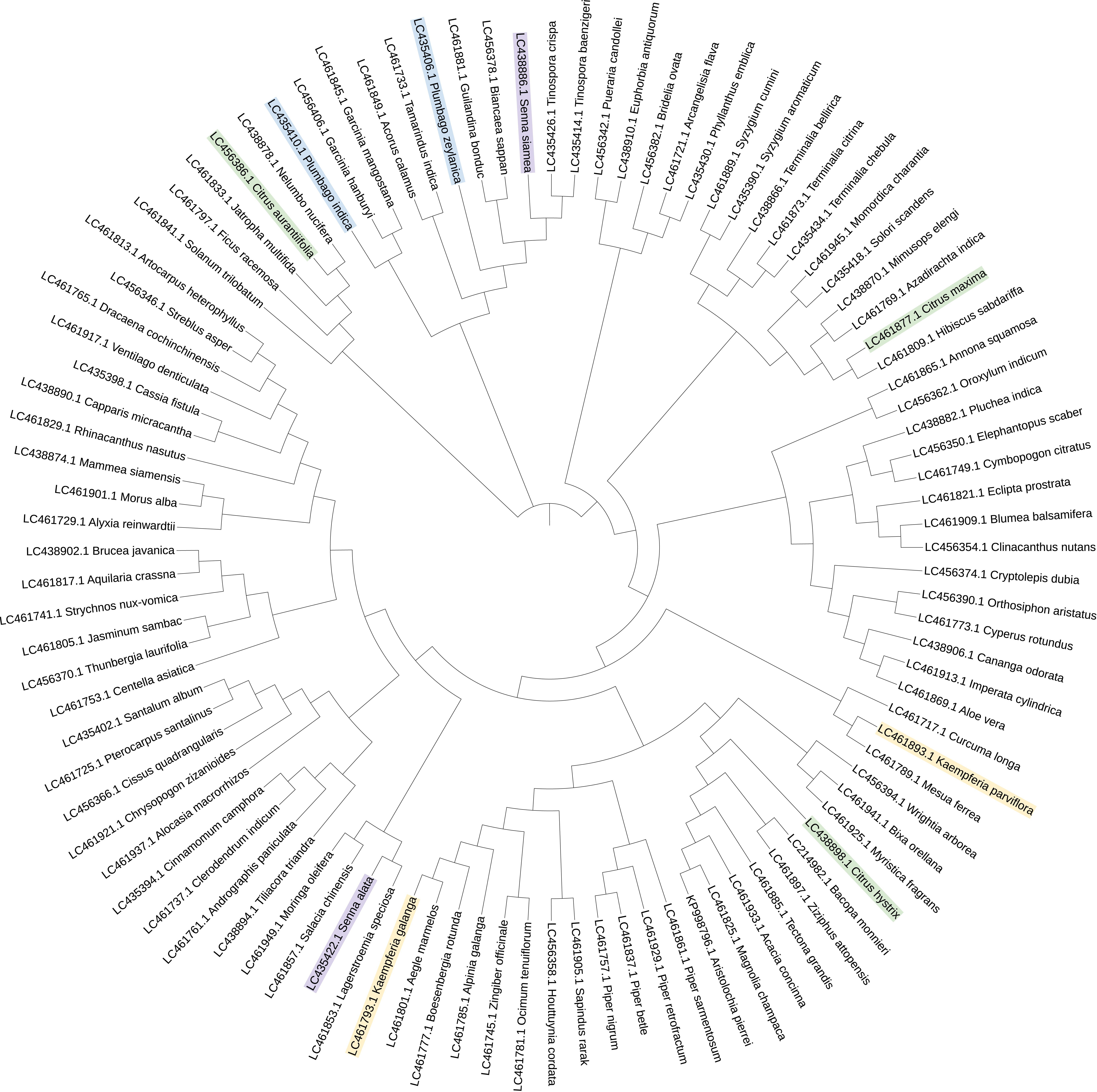

Supplement: Supplementary file 1 — Supplementary Figure 1. [file 41598_2022_13287_MOESM1_ESM.jpg]
