# Supplementary material for: Development of a DNA barcode library of plants in the Thai Herbal Pharmacopoeia and Monographs for authentication of herbal products
Source: Sci Rep. 2022 Jun 10;12:9624. doi: 10.1038/s41598-022-13287-x (PMC9187672; doi:10.1038/s41598-022-13287-x)

| Title | Development of a DNA barcode library of plants in the Thai Herbal Pharmacopoeia and Monographs for authentication of herbal products. |
| --- | --- |
| Authors | Santhosh Kumar J. Urumarudappa^1,2^ , Chayapol Tungphatthong^1,2^ , Jirayut Jaipaew^1,2^, Natapol Pornputtapong^1,3^, Duangkamol Pakdeesattayapong^4^, Sornkanok Vimolmangkang^2^ and Suchada Sukrong^1,2,*^ |
| Affiliation | ^1^ Center of Excellence in DNA Barcoding of Thai Medicinal Plants, Chulalongkorn University, Bangkok 10330, Thailand |
|  | ^2^ Department of Pharmacognosy and Pharmaceutical Botany, Faculty of Pharmaceutical Sciences, Chulalongkorn University, Bangkok 10330, Thailand |
|  | ^3^ Department of Biochemistry and Microbiology, Faculty of Pharmaceutical Sciences, Chulalongkorn University, Bangkok 10330, Thailand |
|  | ^4^ Herbal Products Division, Food and Drug Administration. Ministry of Public Health, Bangkok 11000, Thailand |
| *Corresponding author | Professor Suchada Sukrong, Ph.D. |
|  | Center of Excellence in DNA Barcoding of Thai Medicinal Plants, |
|  | Department of Pharmacognosy and Pharmaceutical Botany, |
|  | Faculty of Pharmaceutical Sciences, Chulalongkorn University, |
|  | Bangkok 10330, Thailand |
|  | Phone: +6681-819-6742, Fax: +6622-558-227 |
|  | Email: suchada.su@chula.ac.th |

**Table. S3:** Nucleotide BLAST results from the 20 herbal samples.

**1_*Bacopa monnieri***

ITS


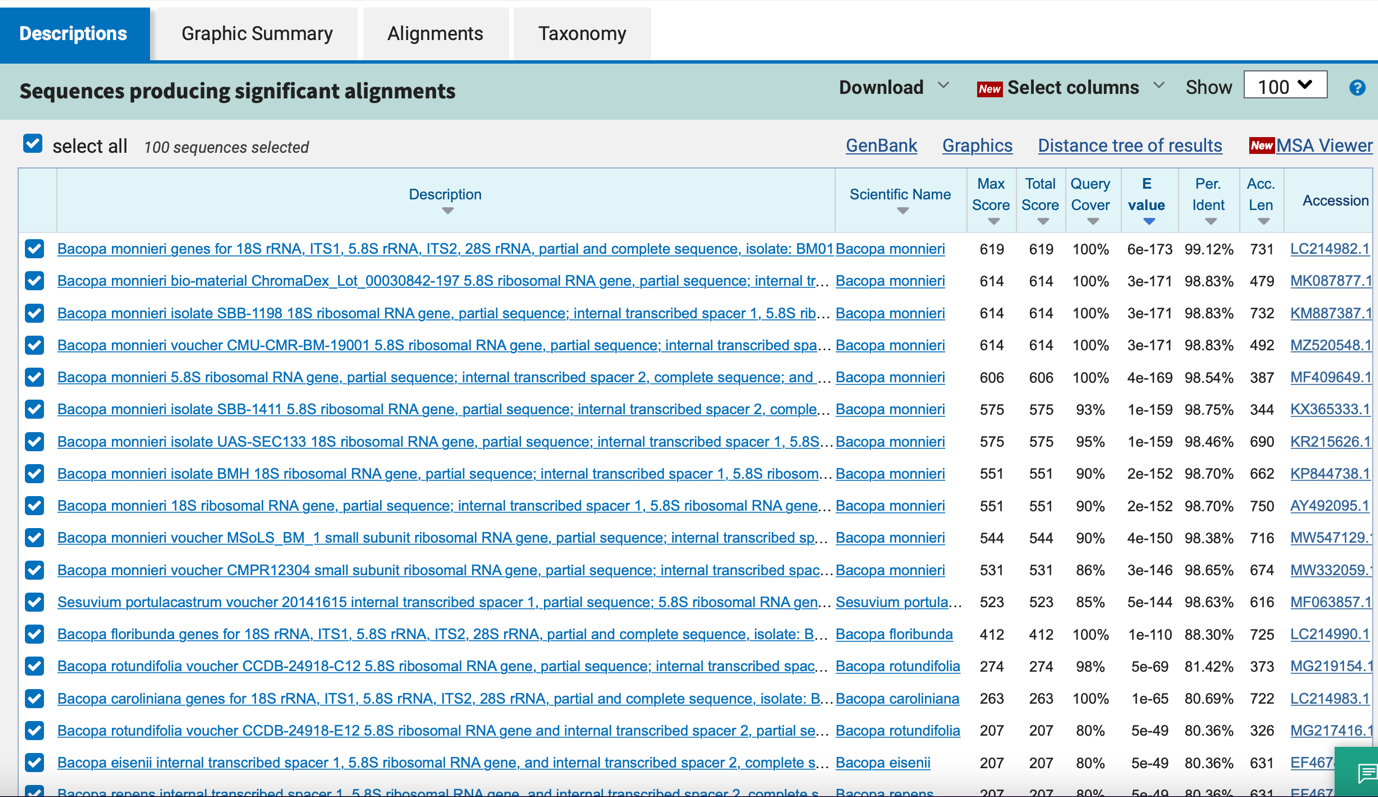


*mat*K


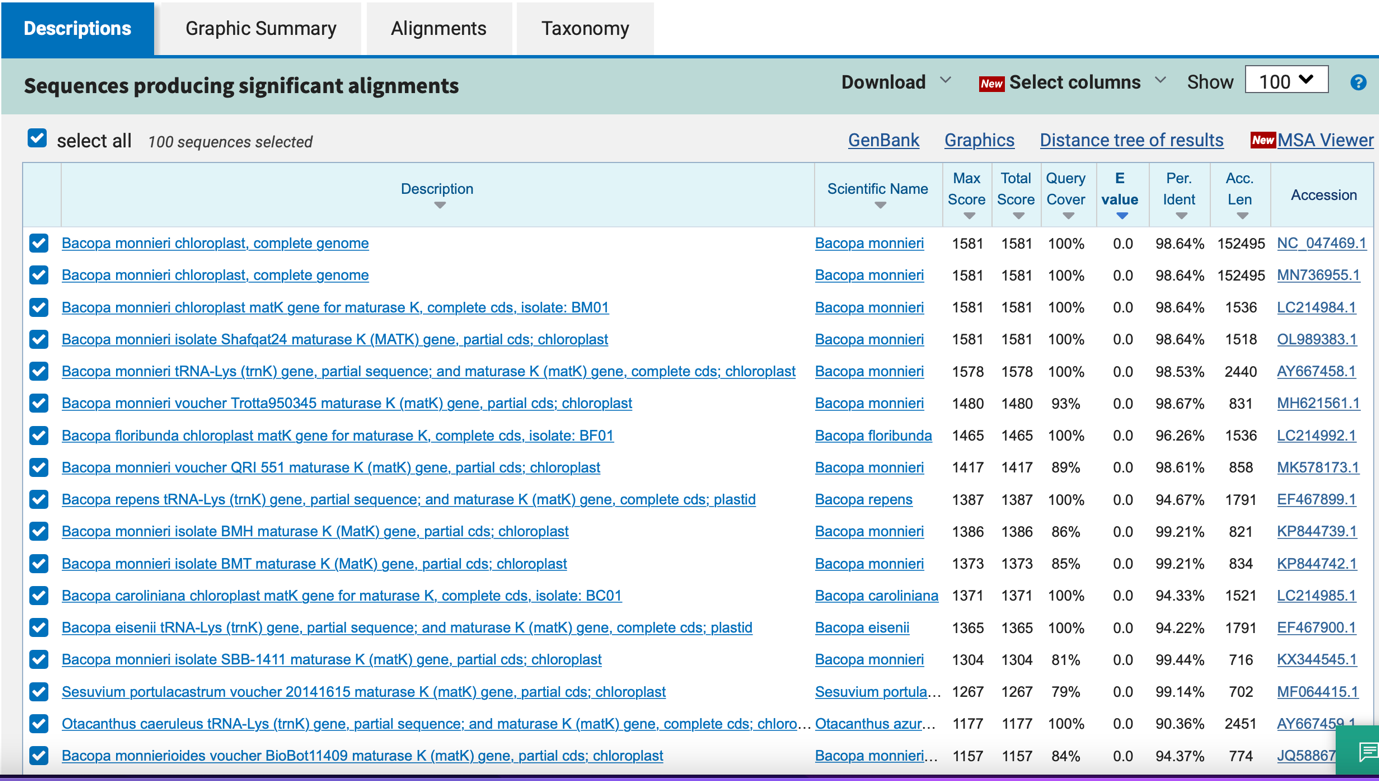


*psb*A_*trn*H

*
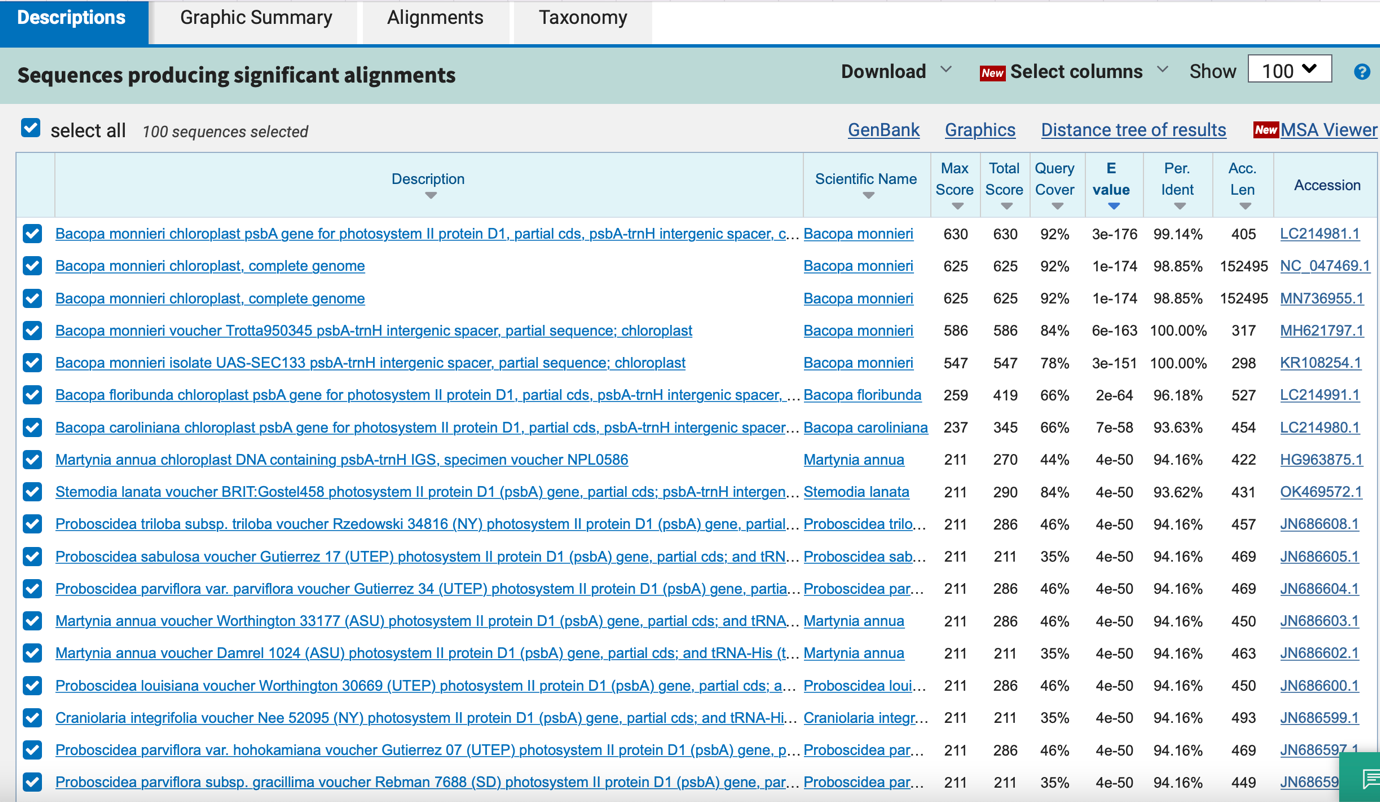
*

*rbc*L


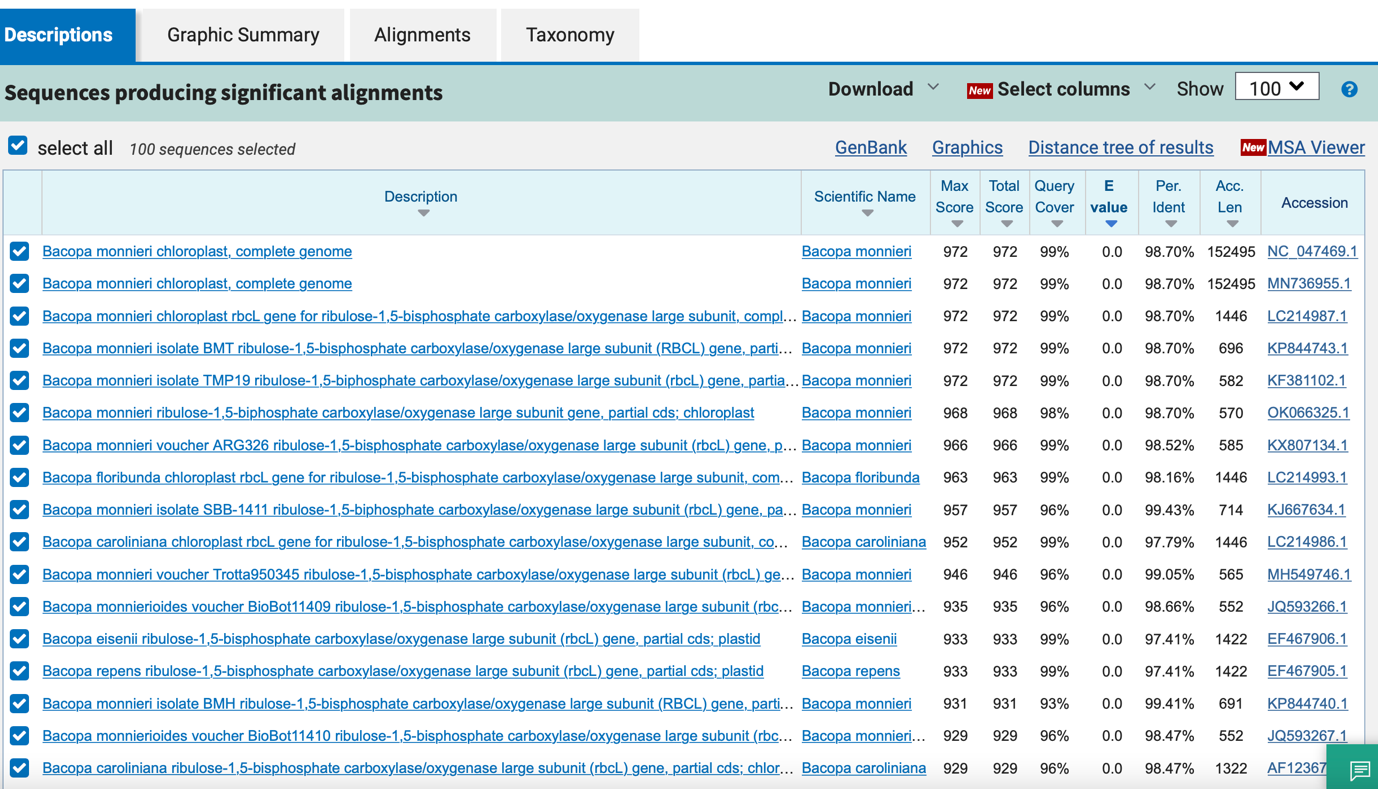


**2_ *Aristolochia pierrei***

ITS


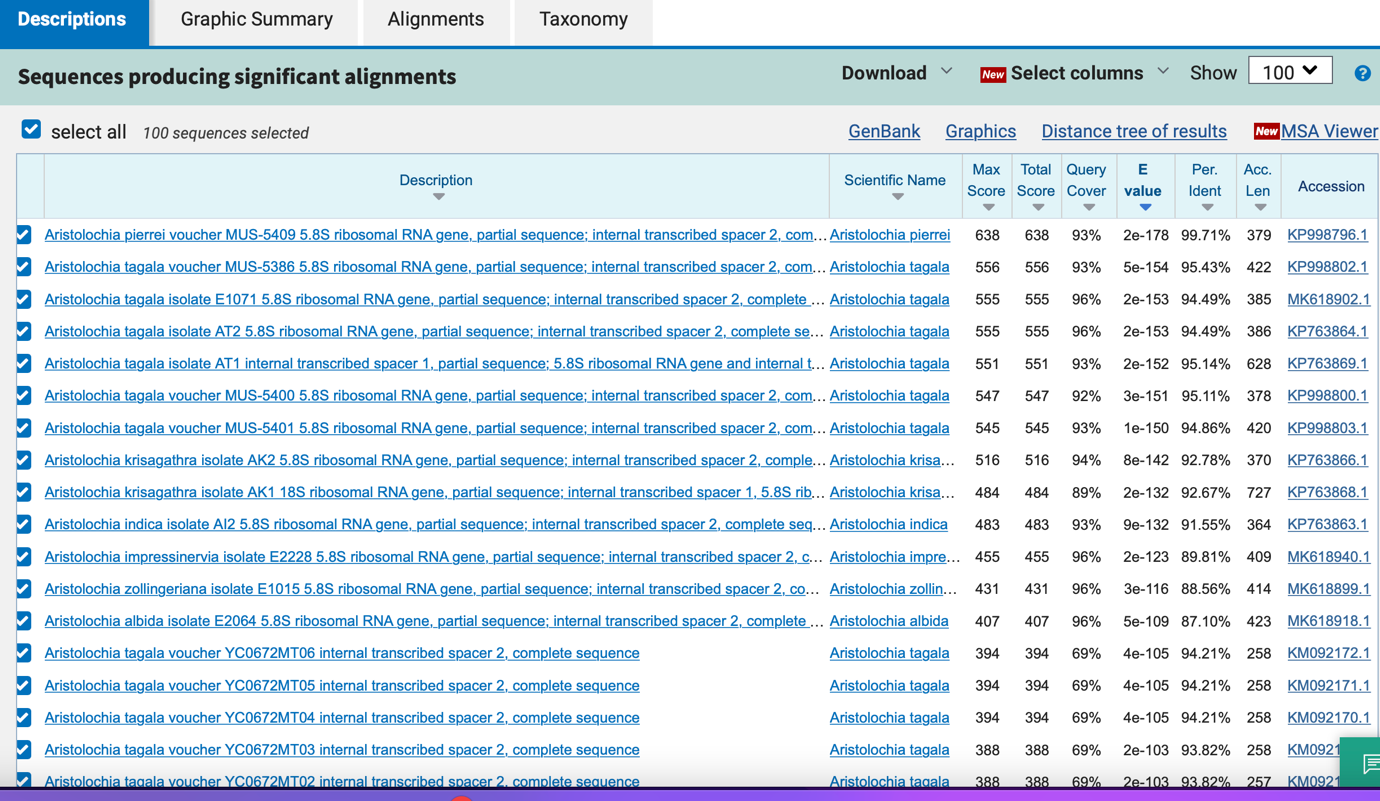


*mat*K


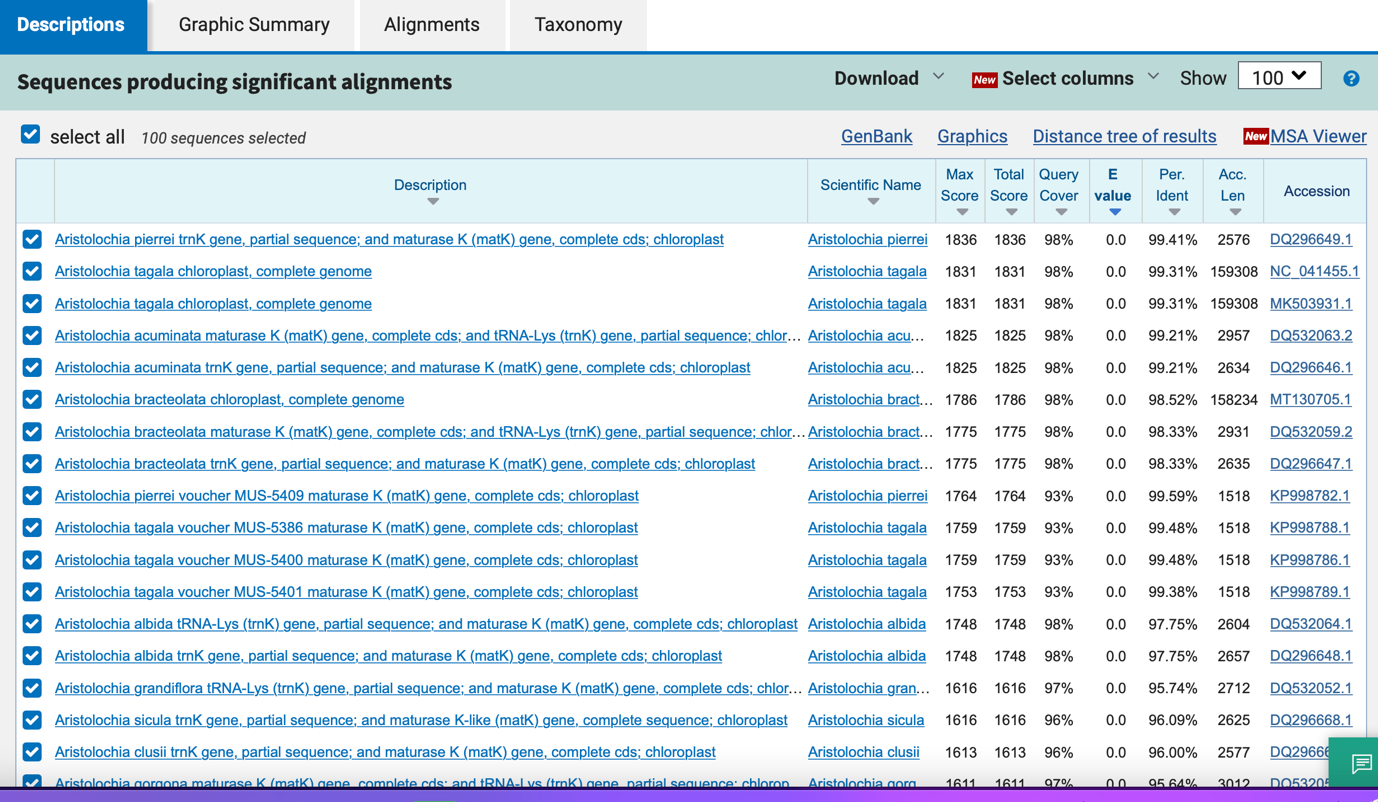


*psb*A_*trn*H


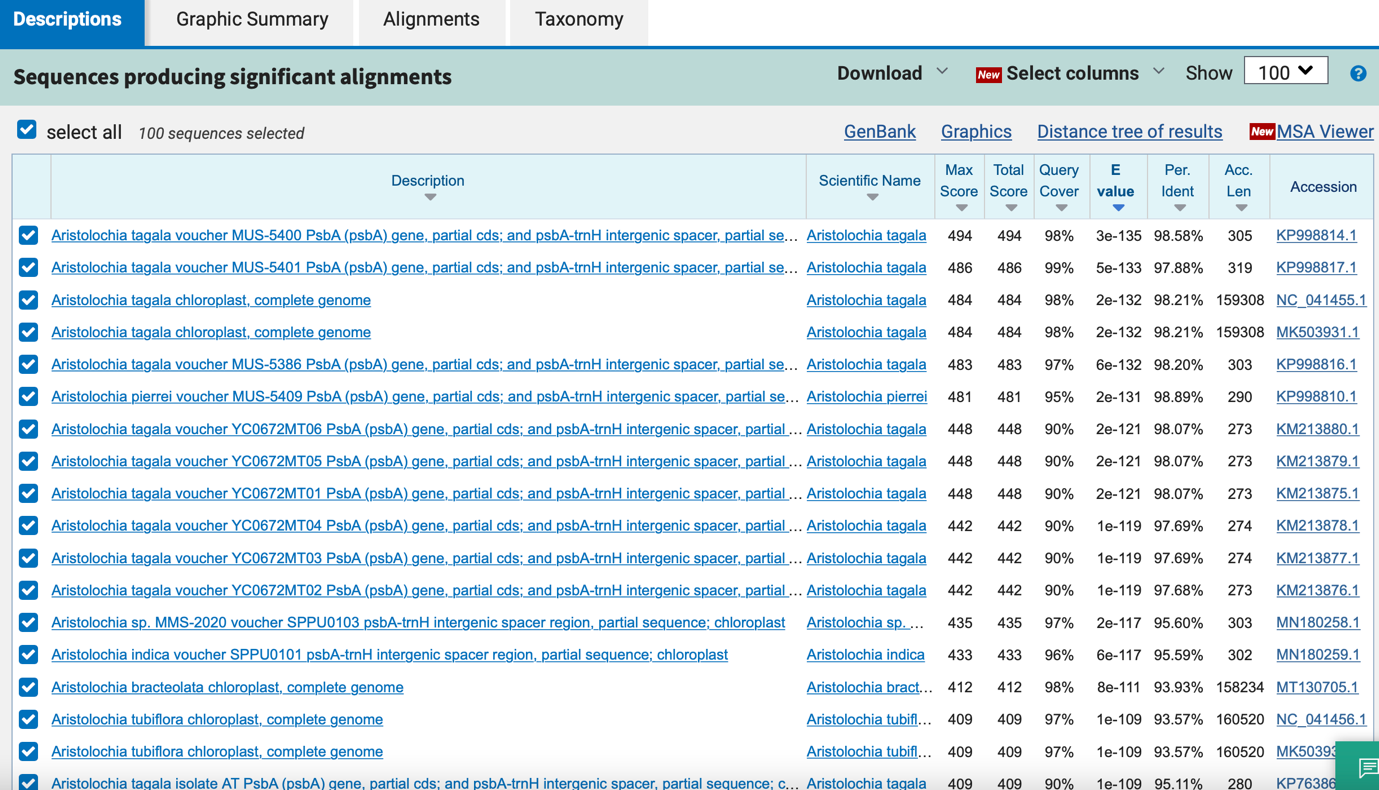


*rbc*L

***
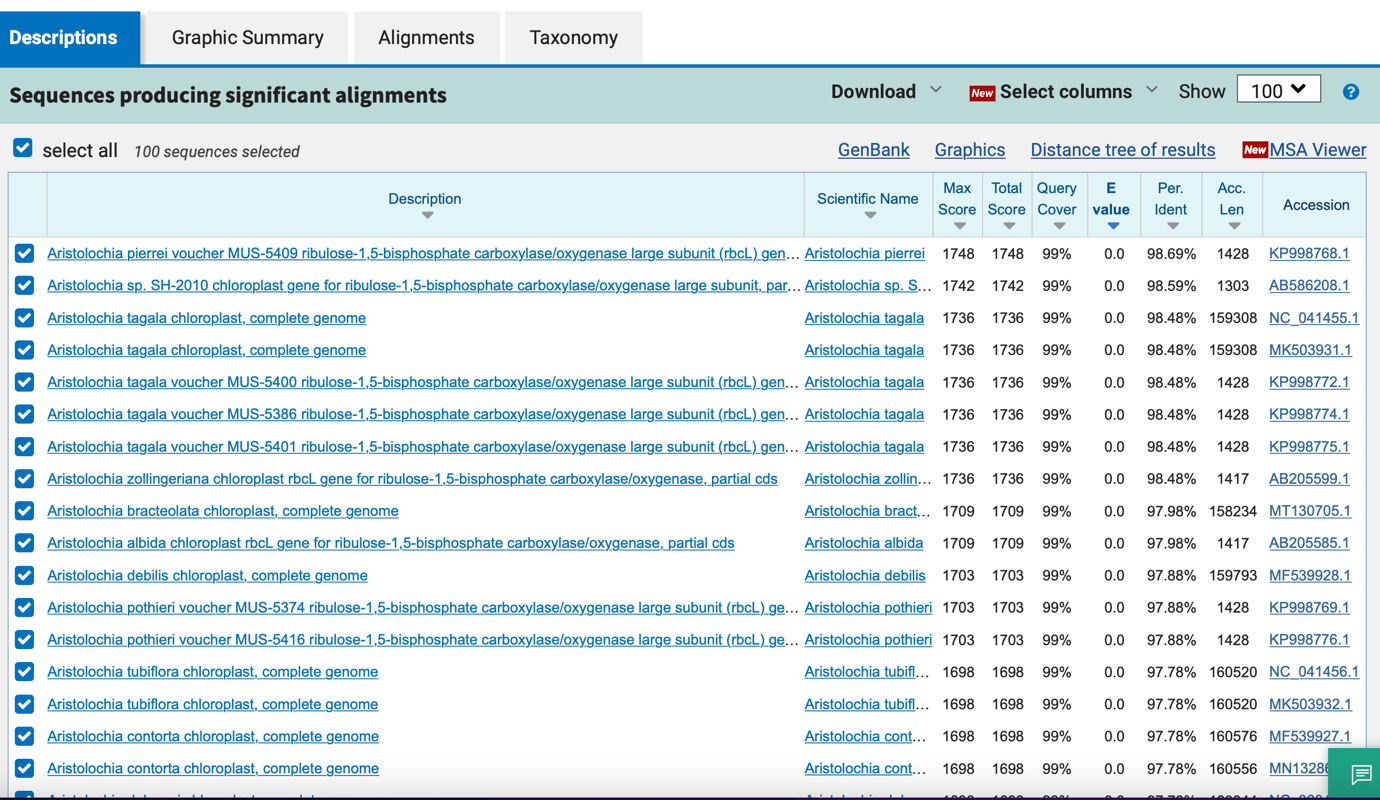
***

**3_*Cyanthillium cinereum***

ITS


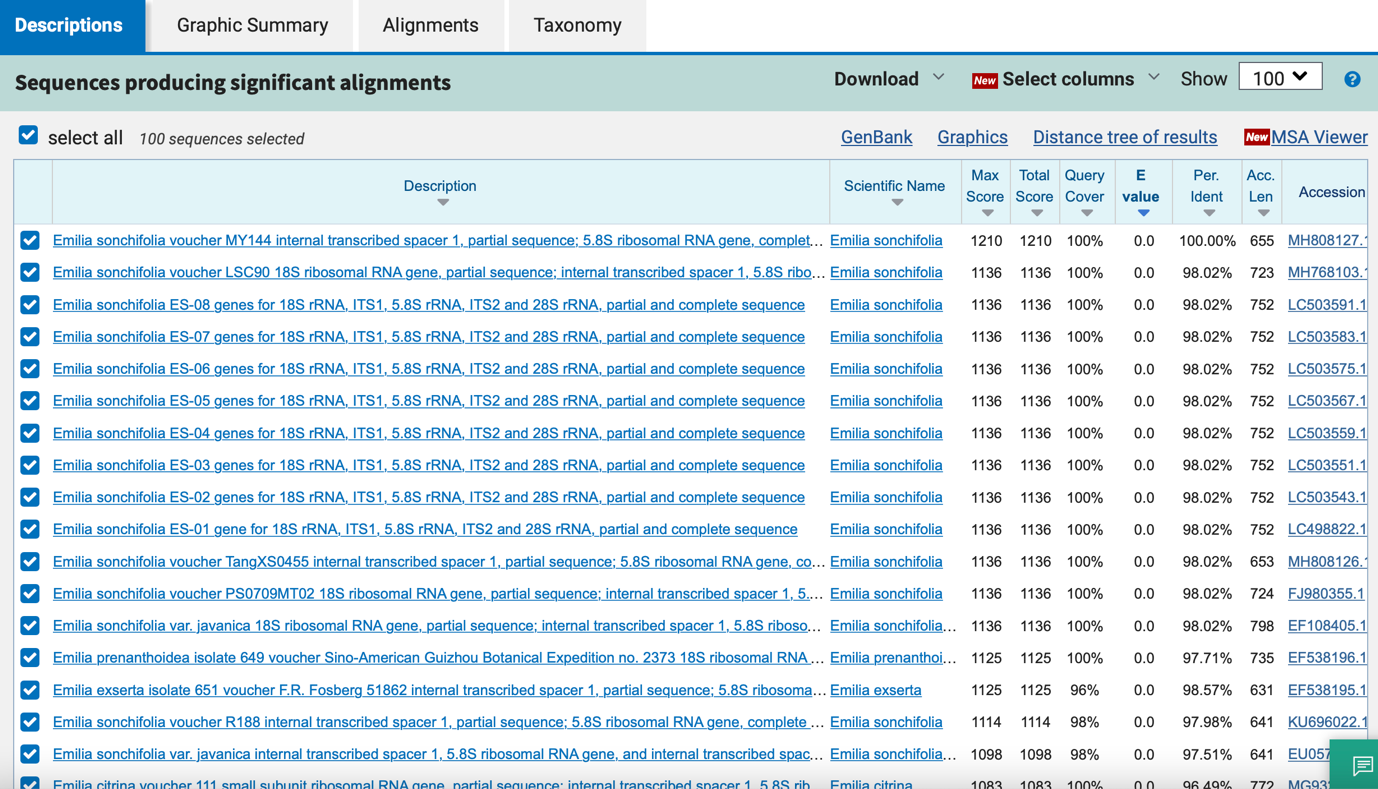


*mat*K


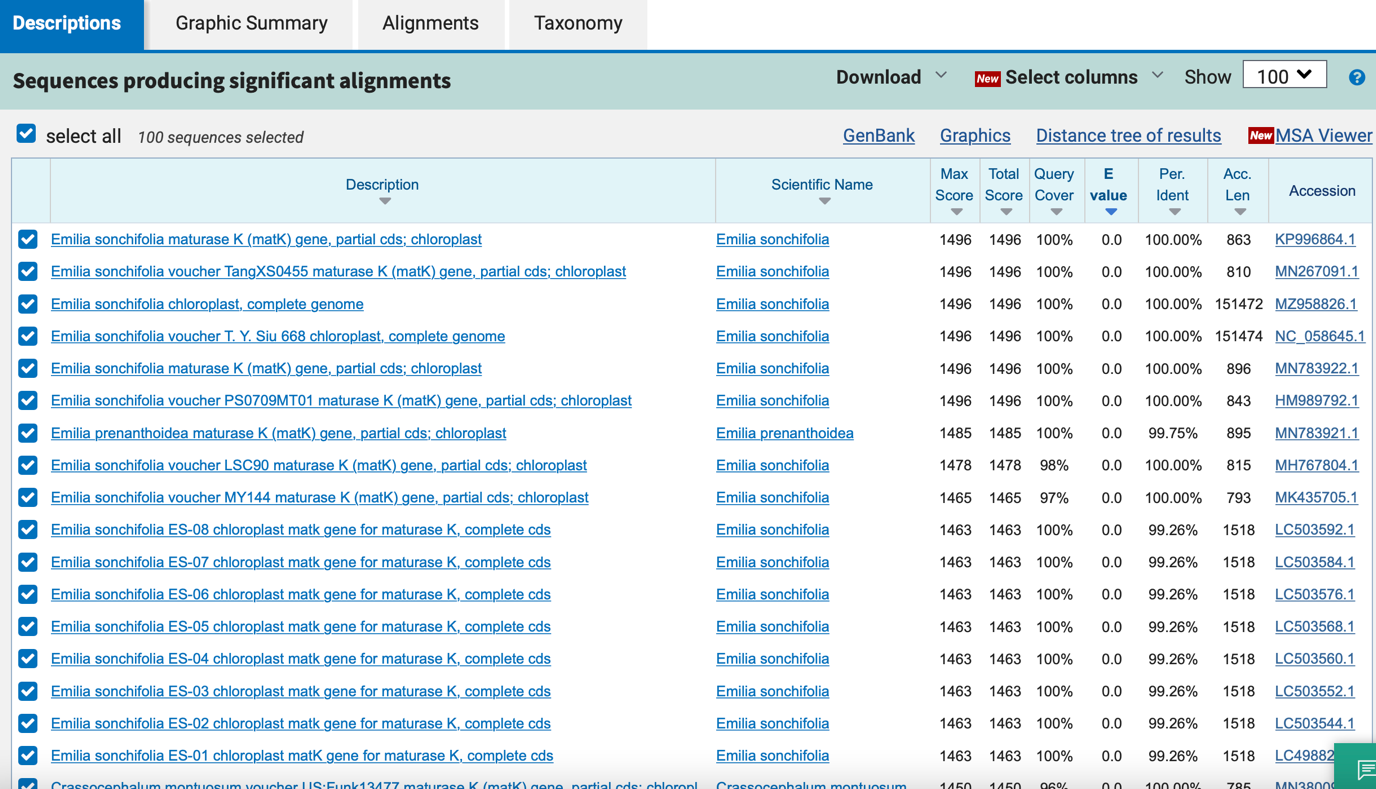


*psb*A_*trn*H


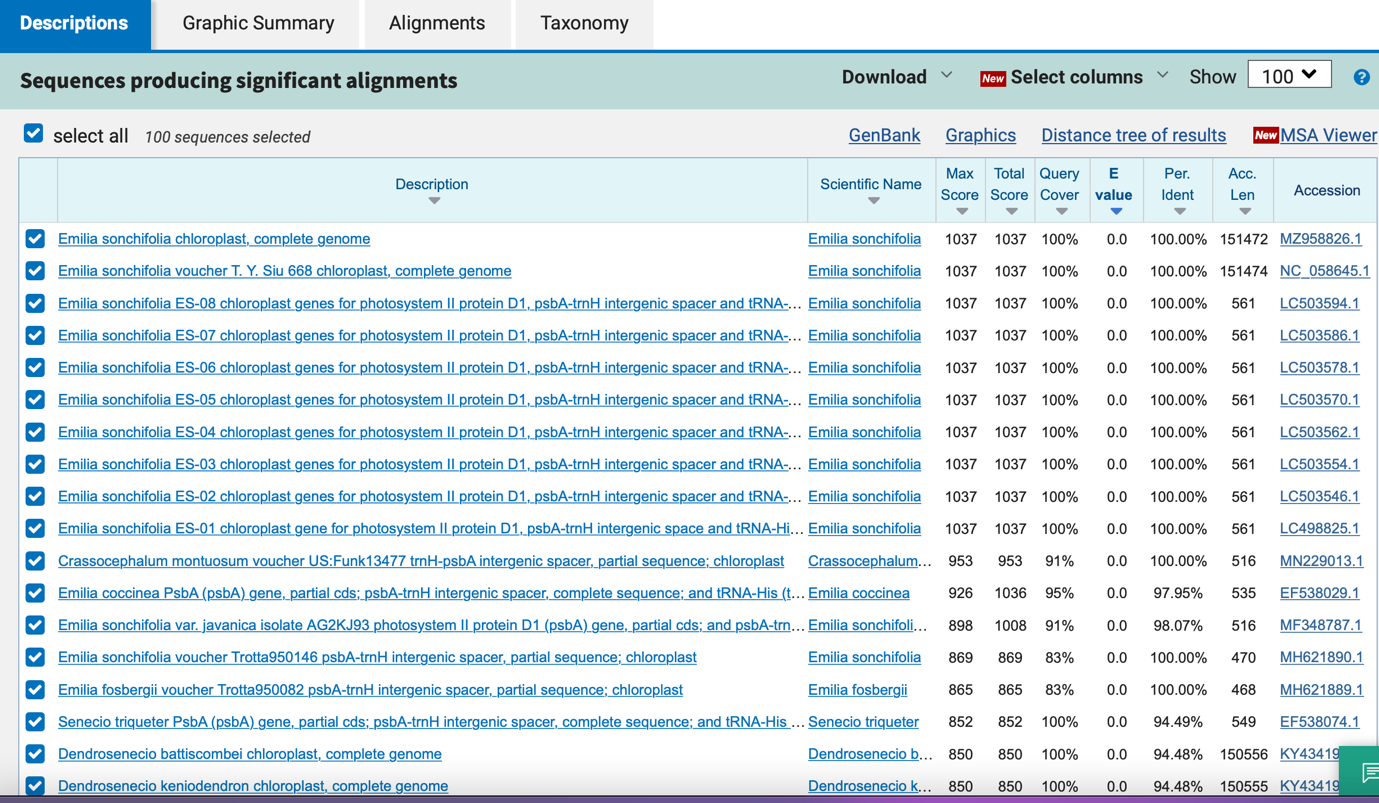


*rbc*L

**
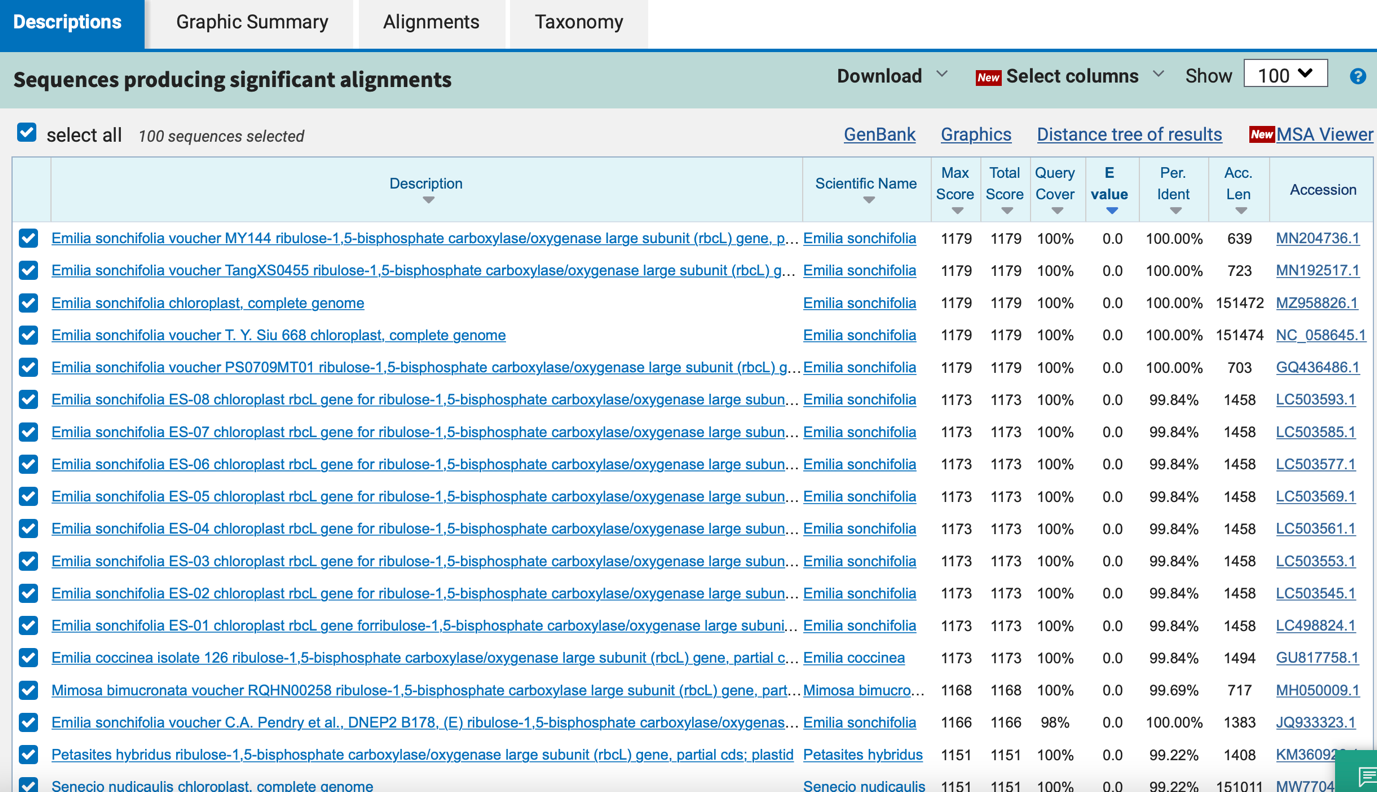
**

**4_** ***Thunbergia laurifolia***

ITS


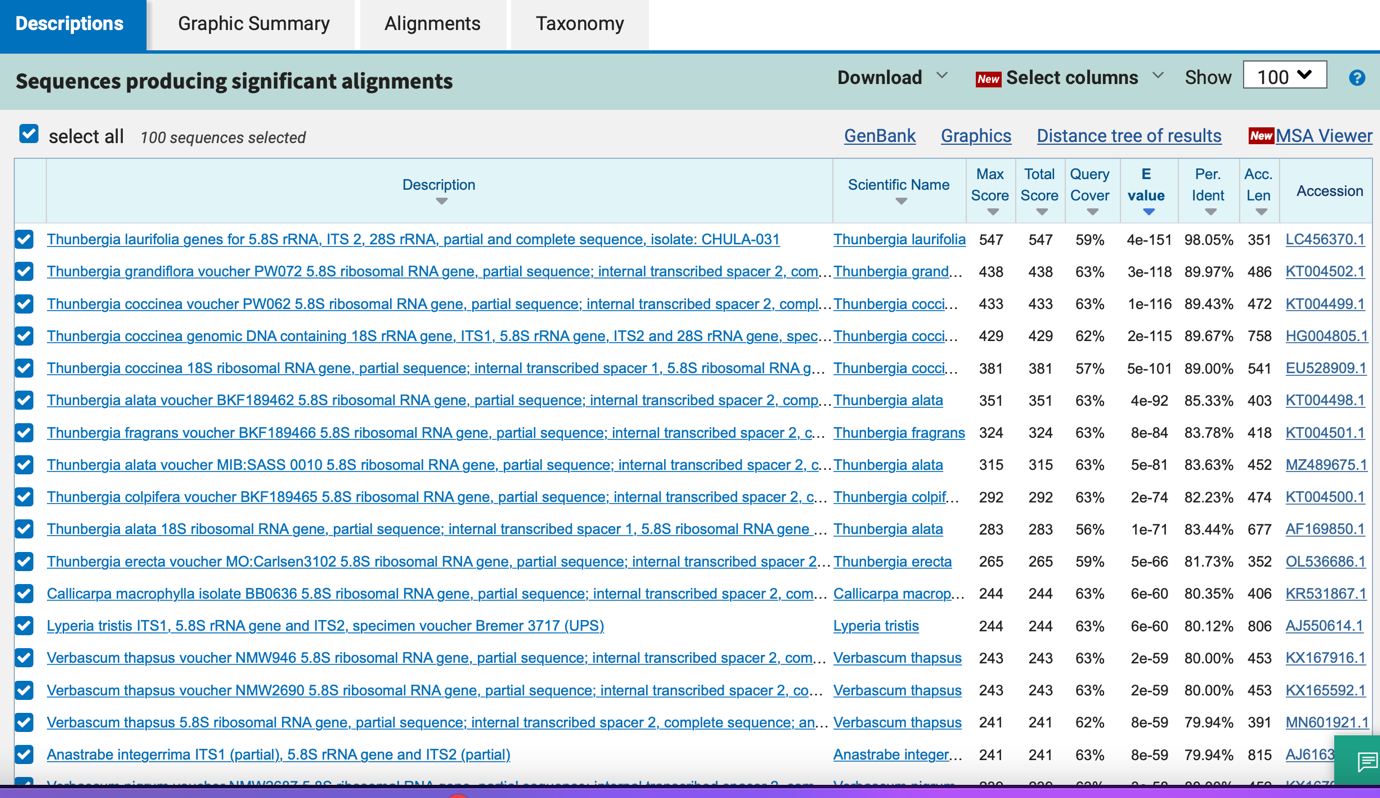


*mat*K


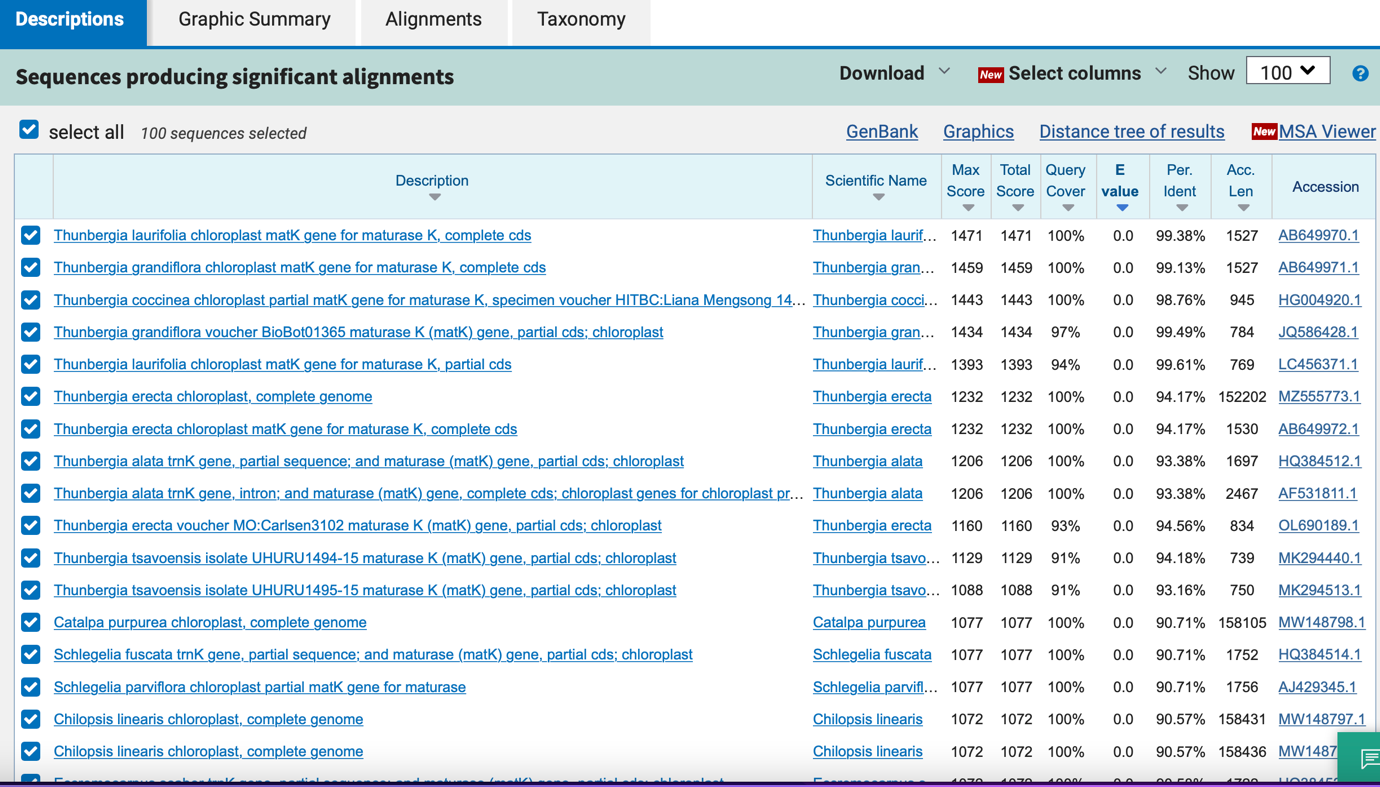


*psb*A_*trn*H


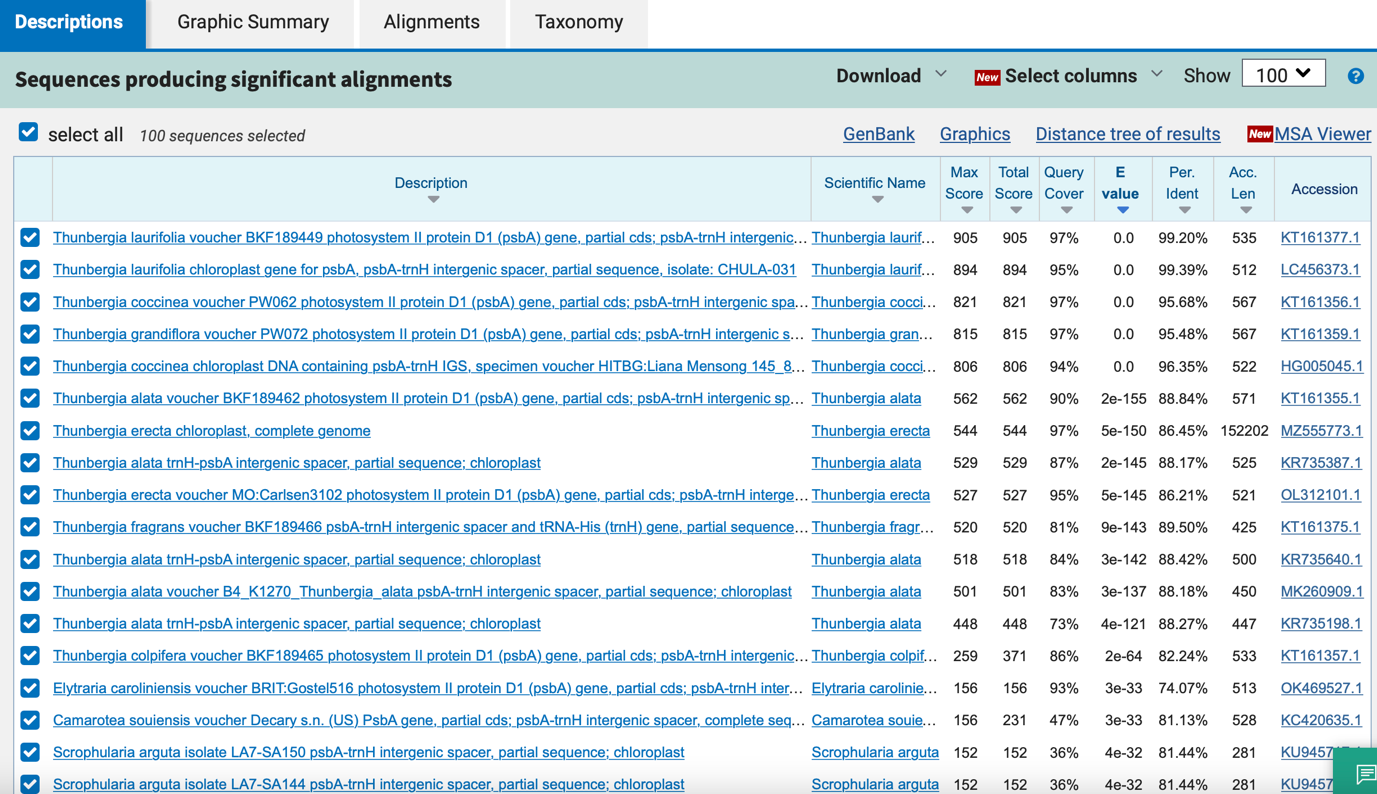


*rbc*L


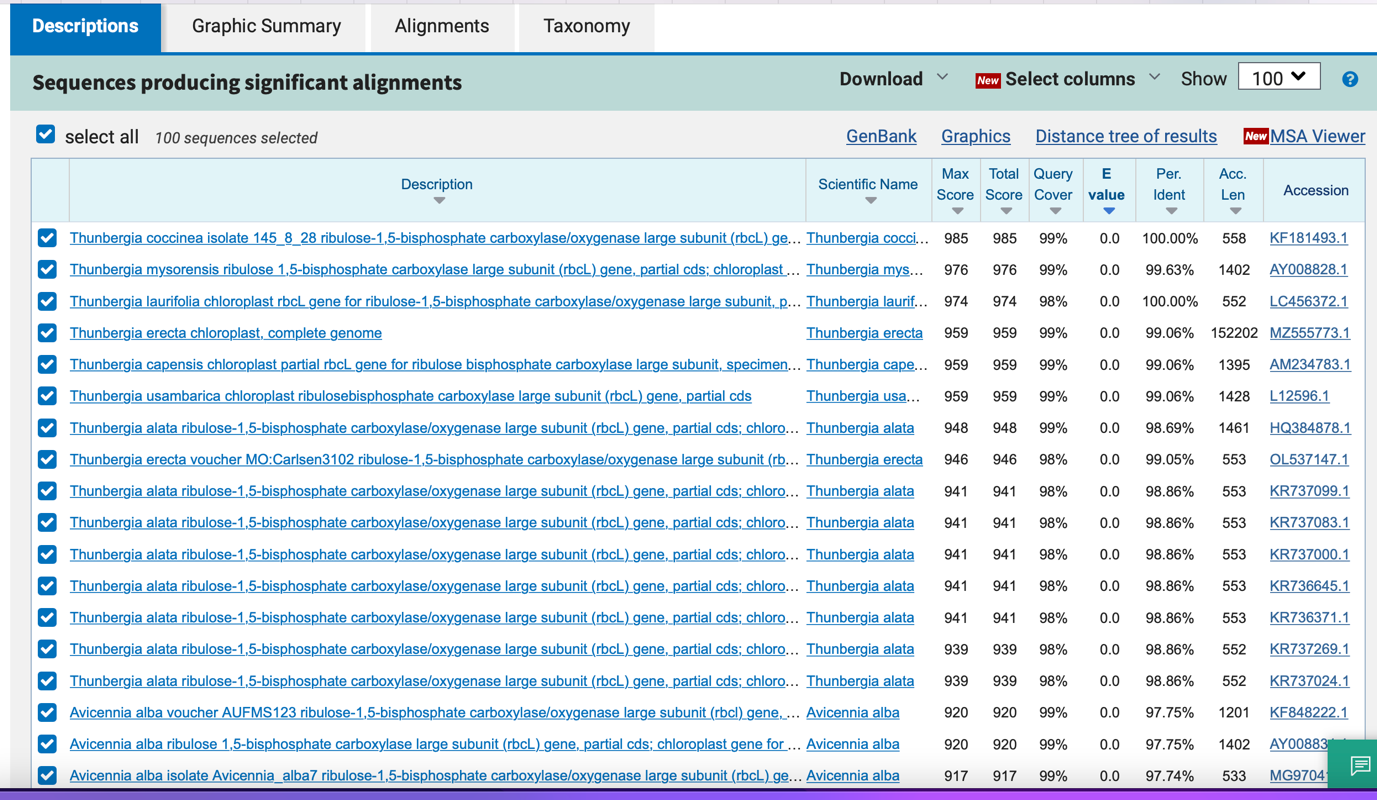


**5_** ***Phyllanthus emblica***

ITS


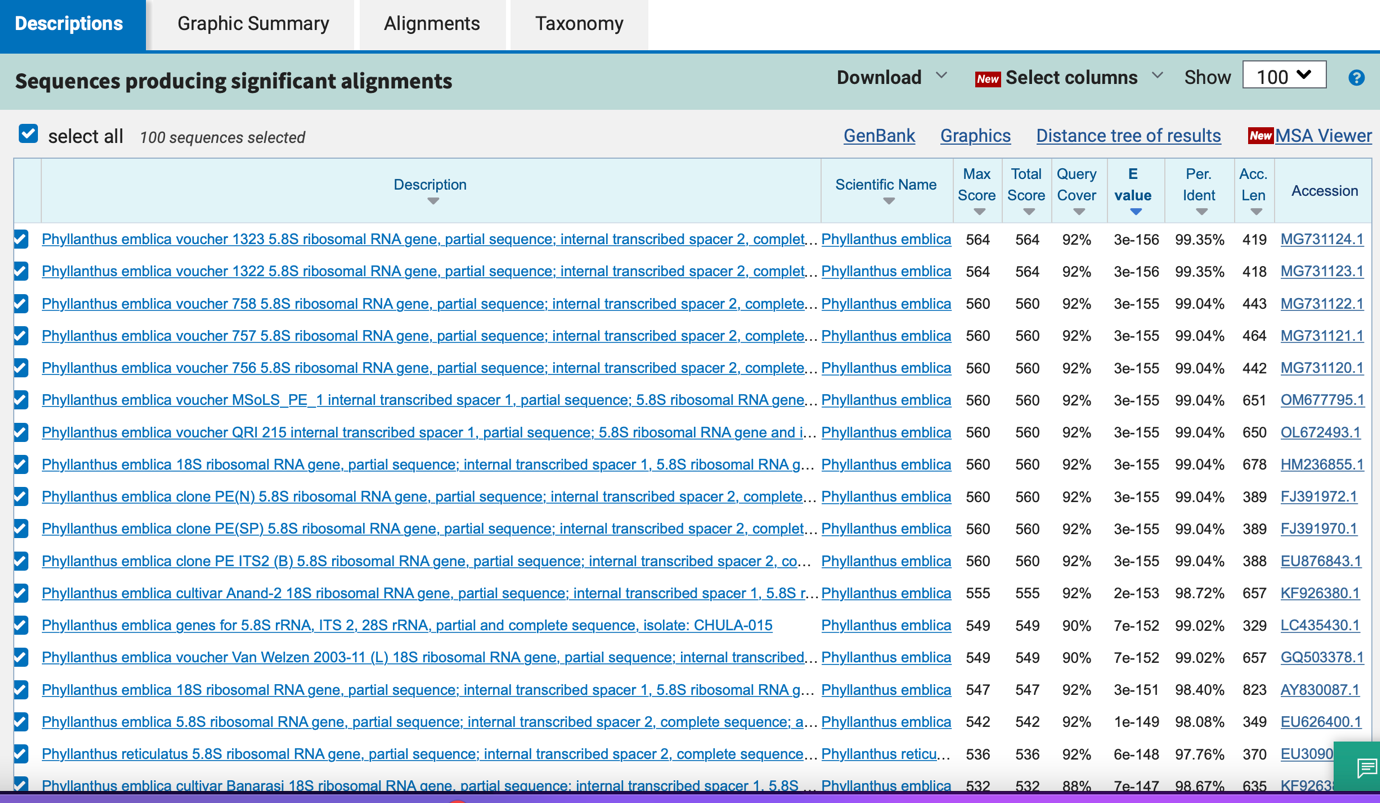


*mat*K


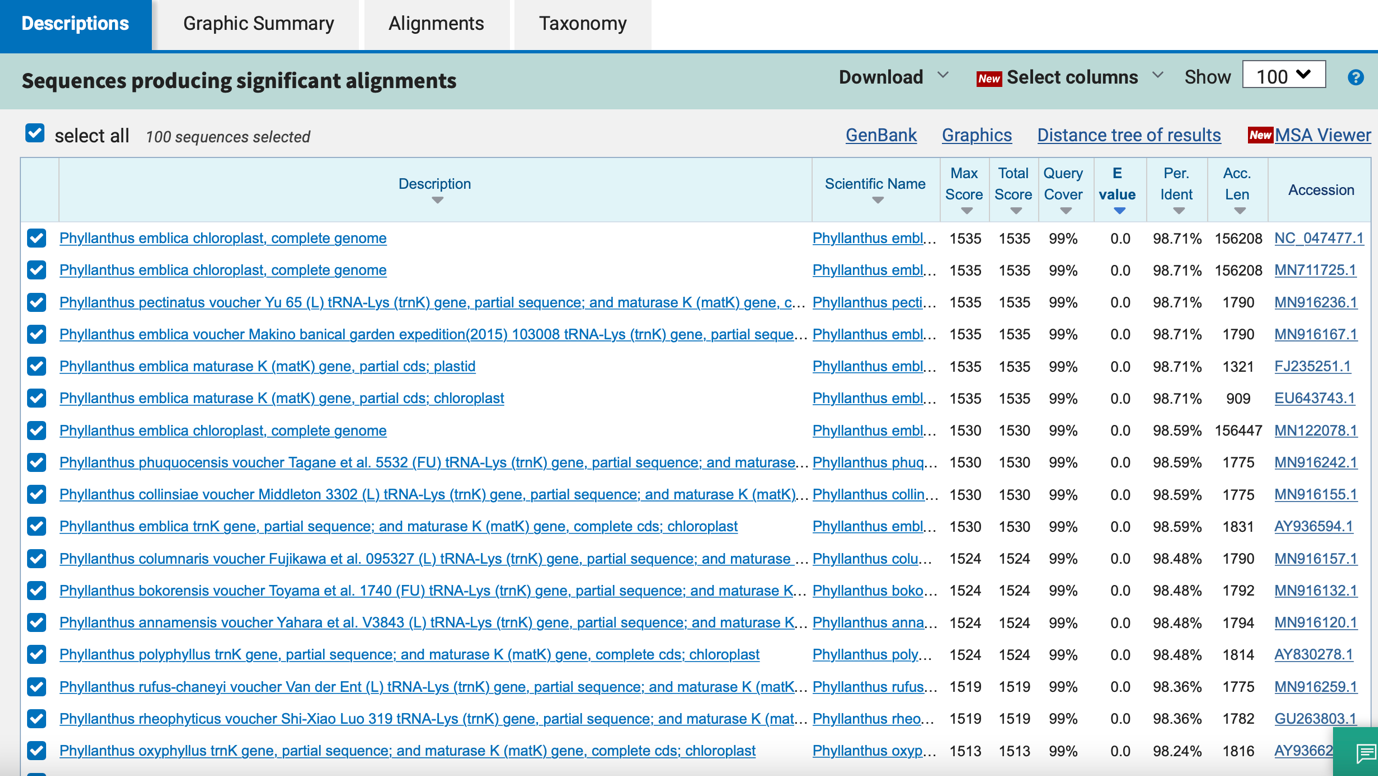


*psb*A_*trn*H


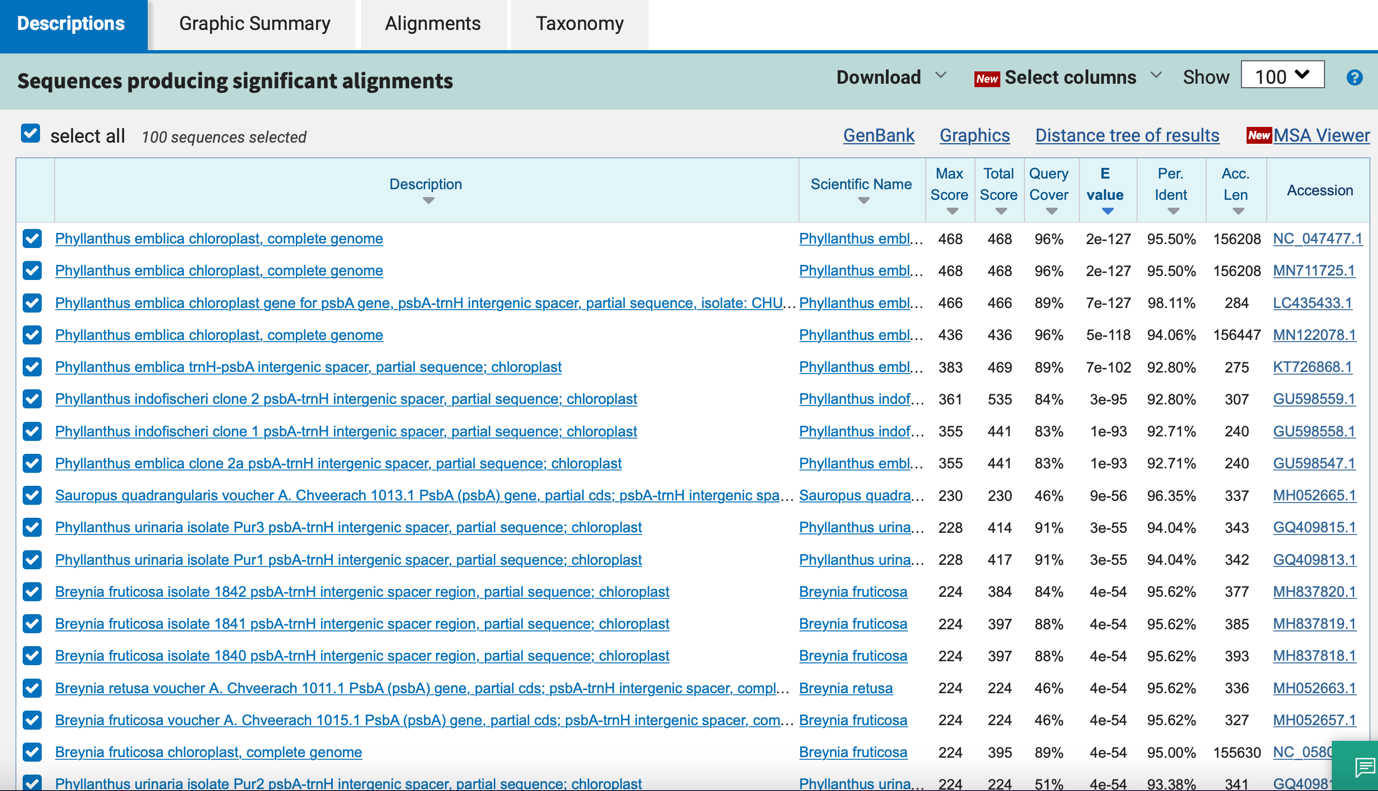


*rbc*L


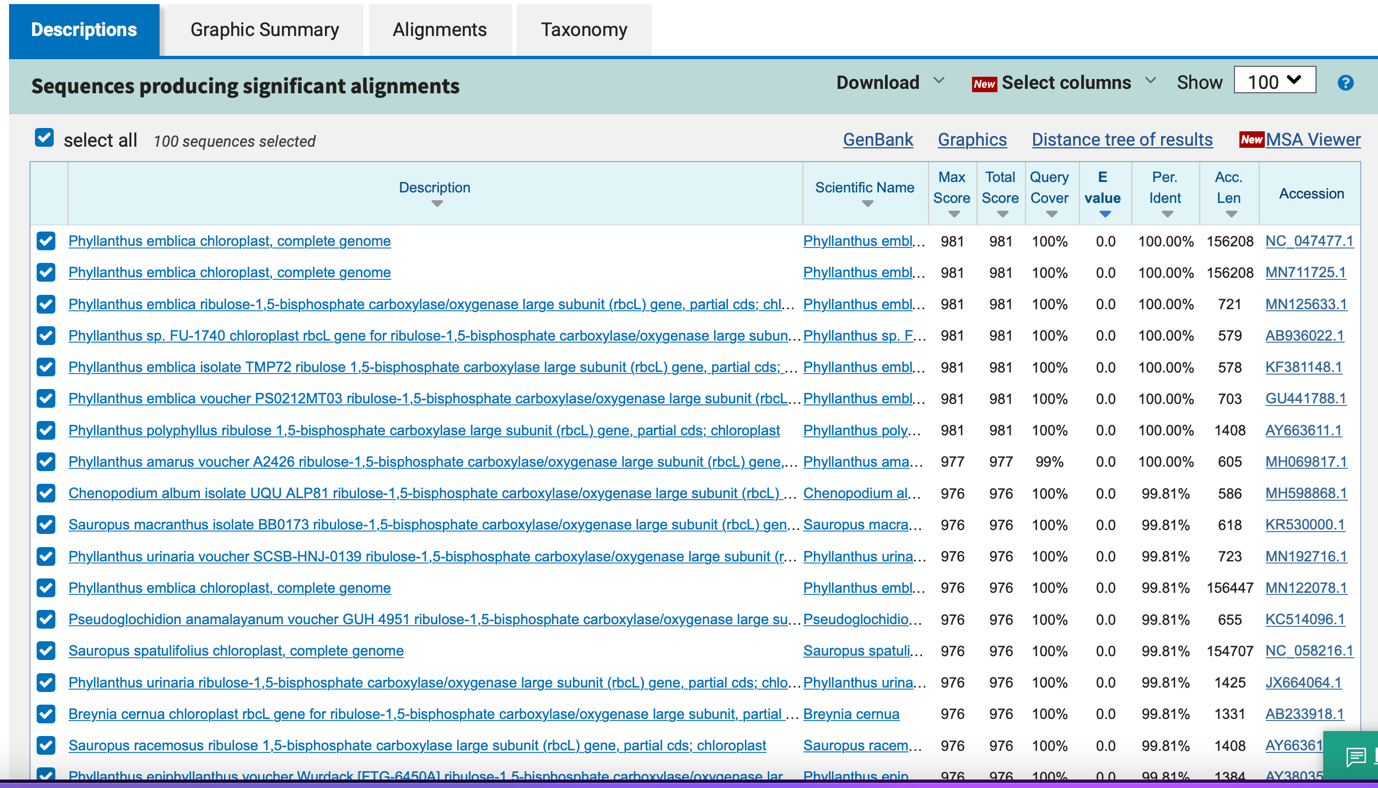


**6_** ***Andrographis paniculata***

ITS


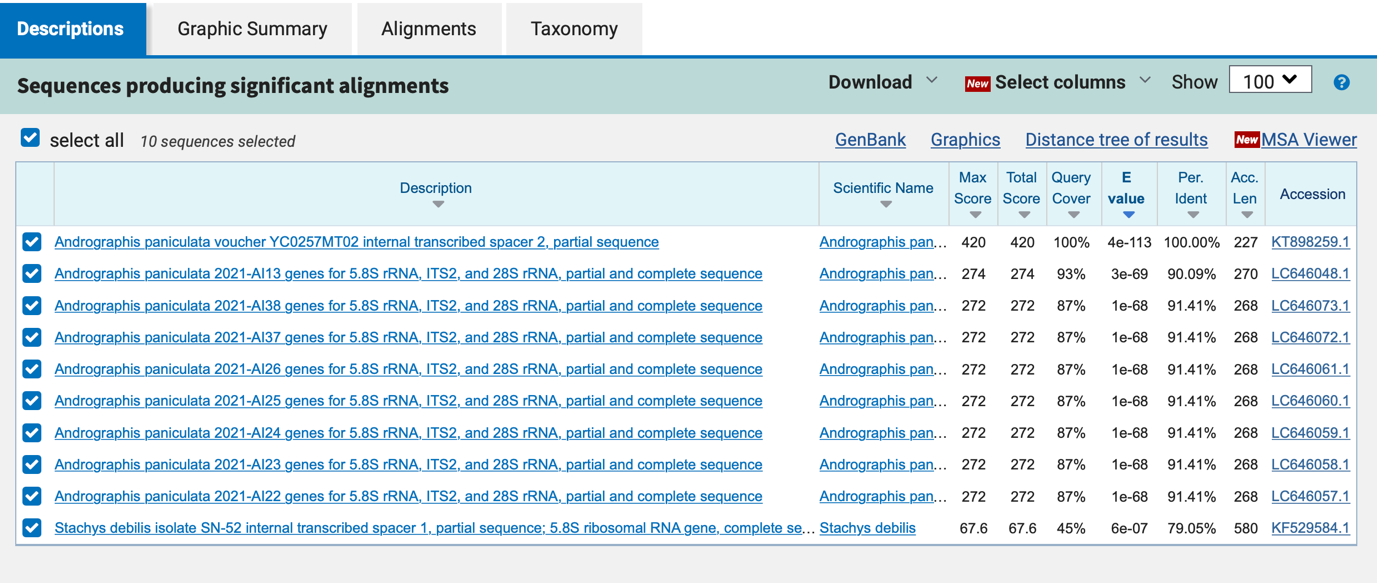


*mat*K


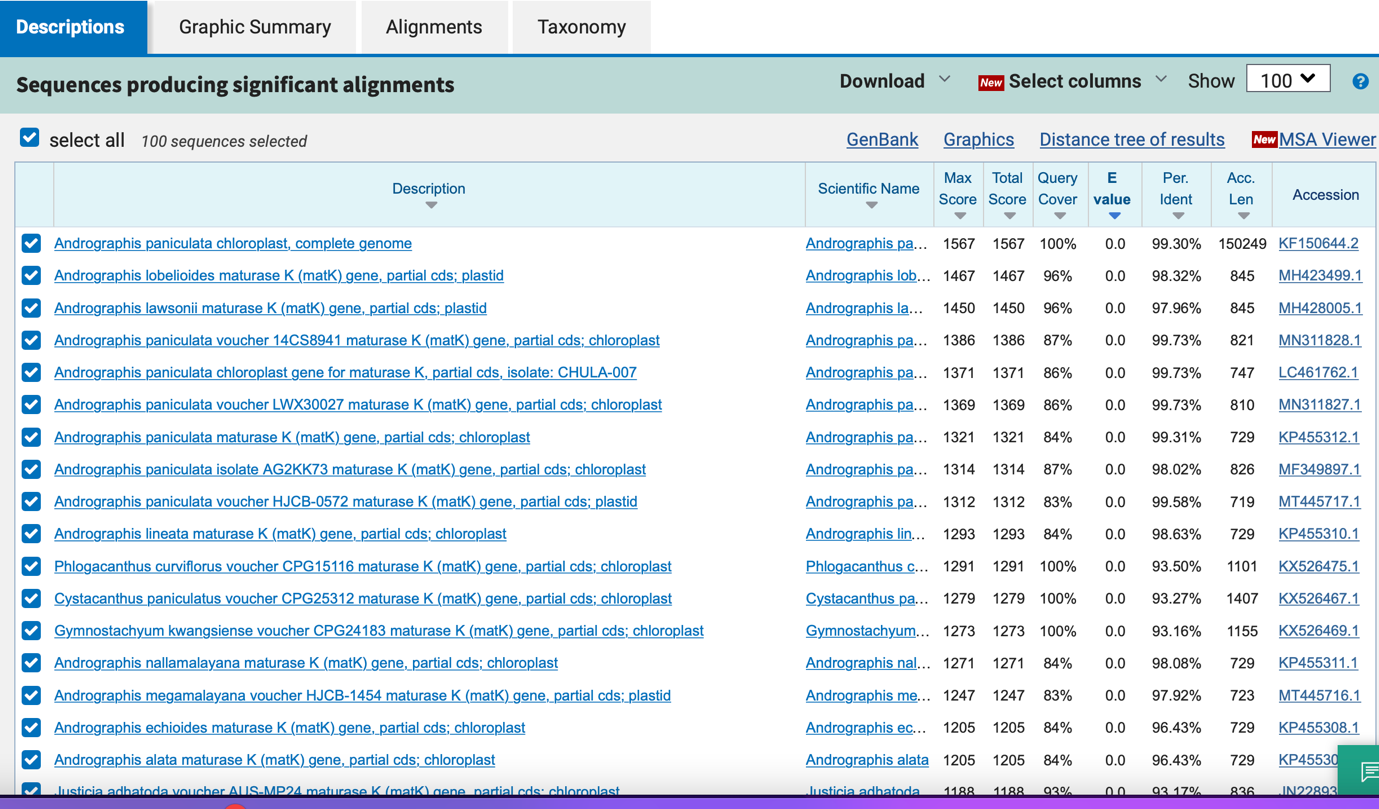


*psb*A_*trn*H


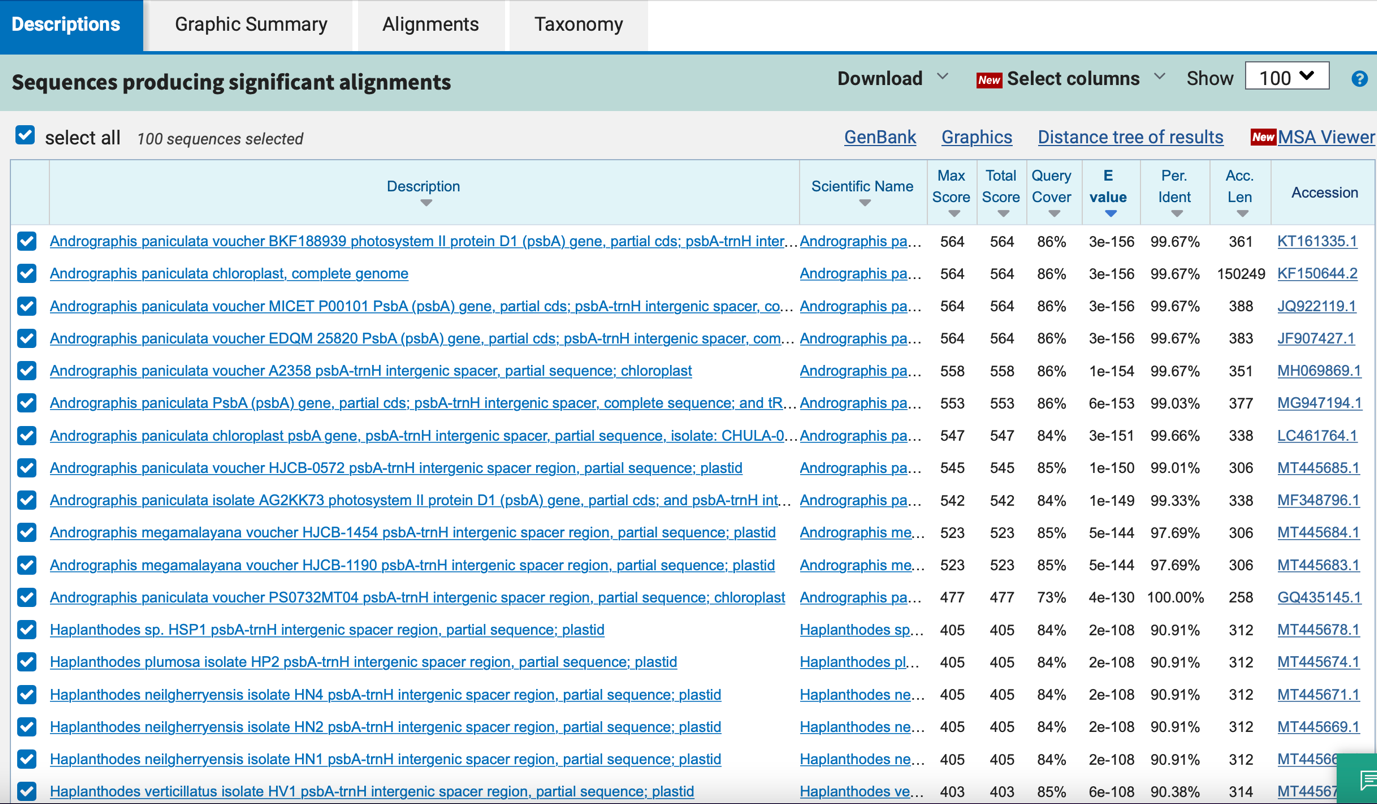


*rbc*L


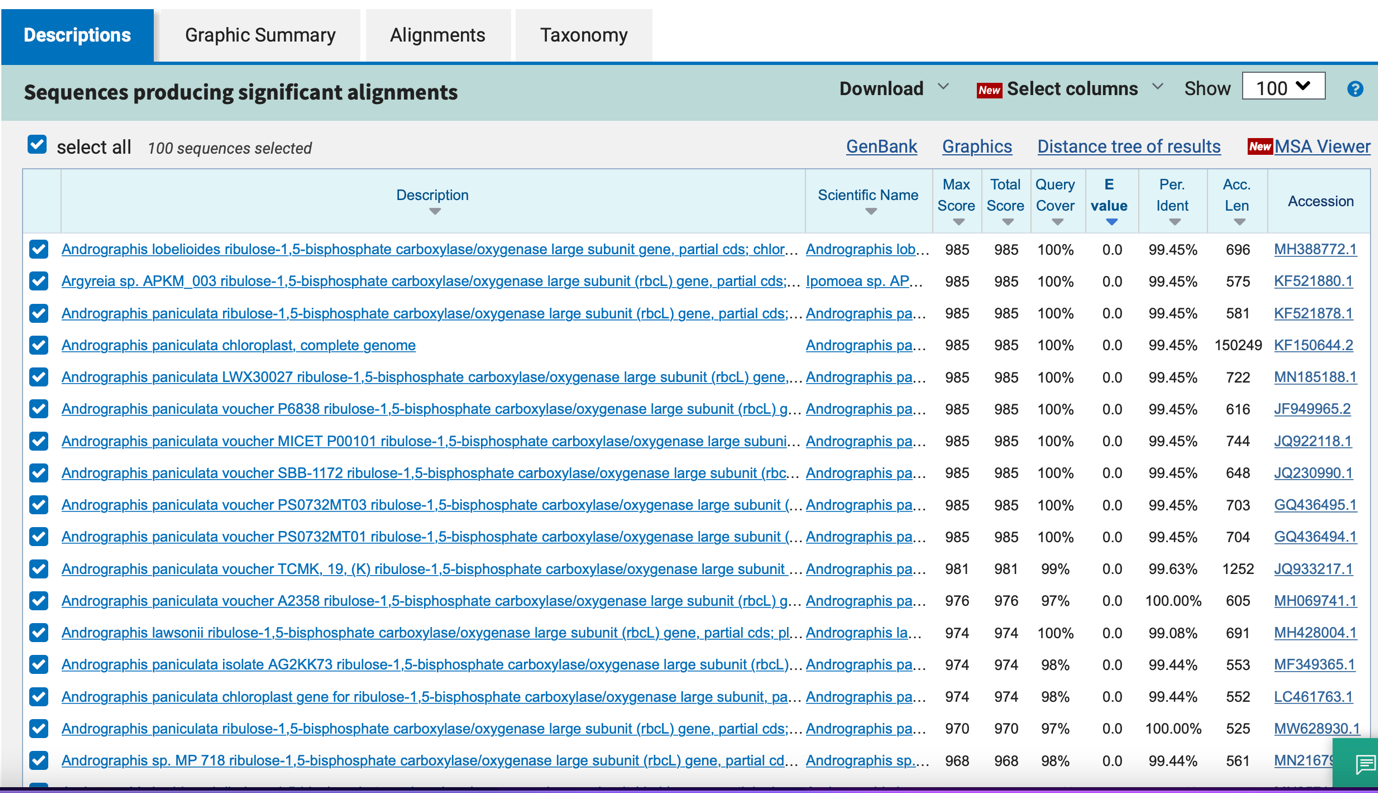


**7_** ***Pueraria candollei***

ITS


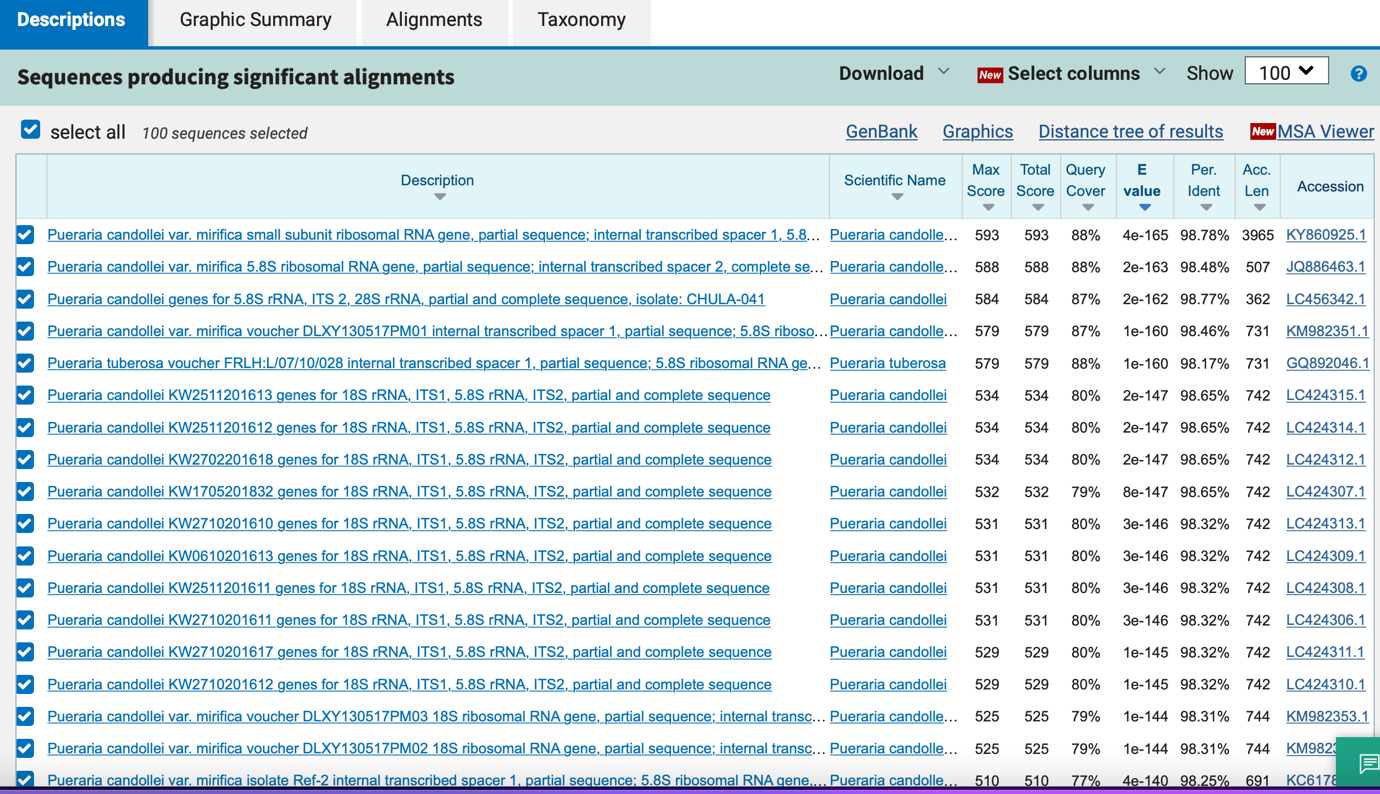


*mat*K


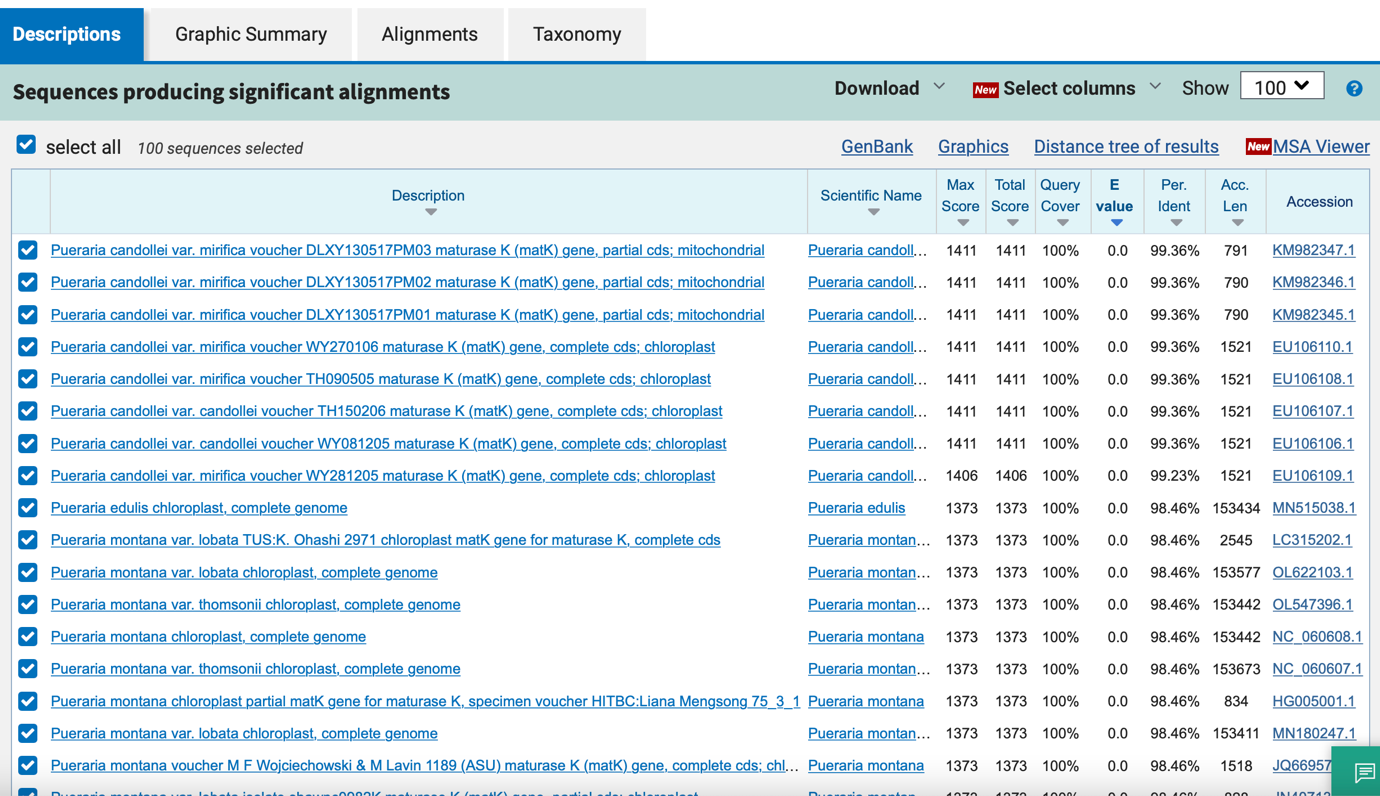


*psb*A_*trn*H


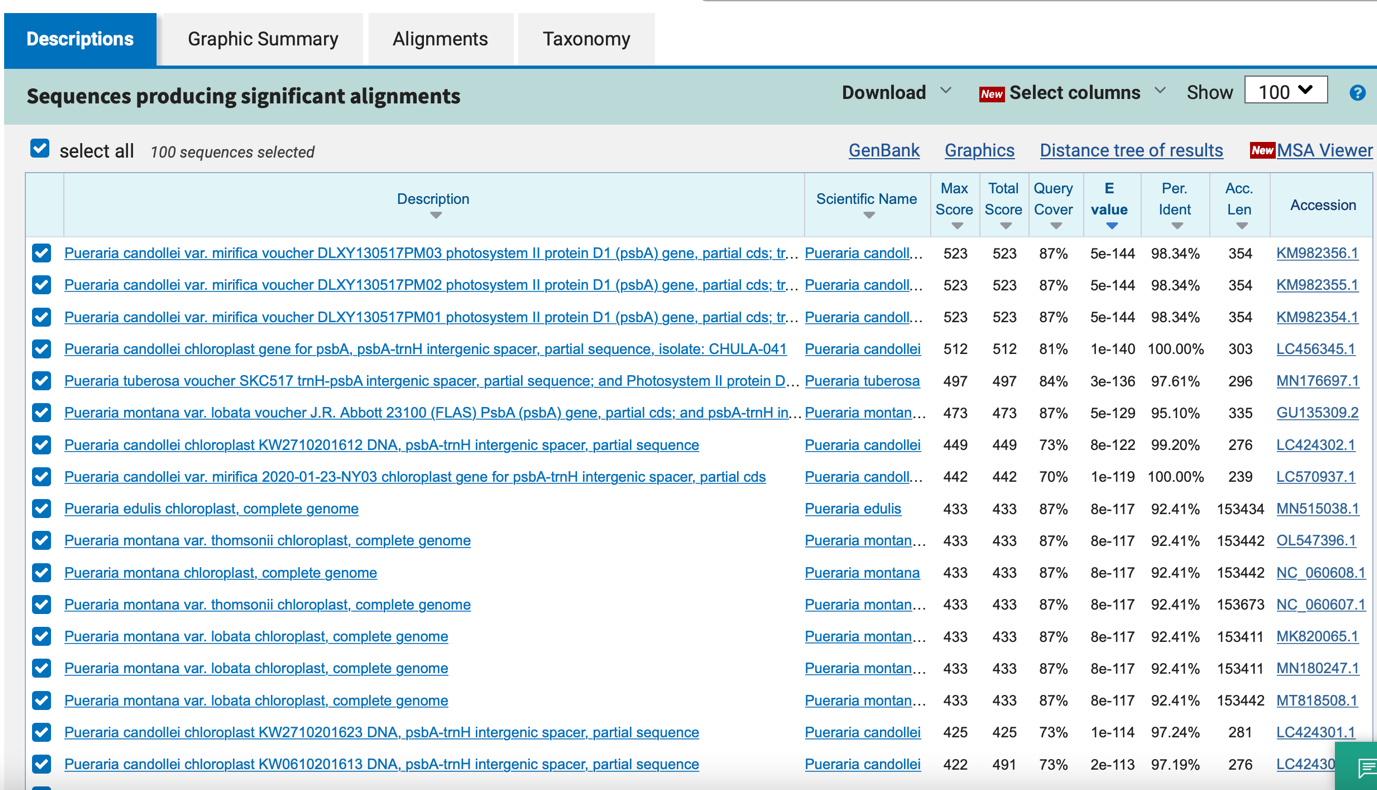


*rbc*L


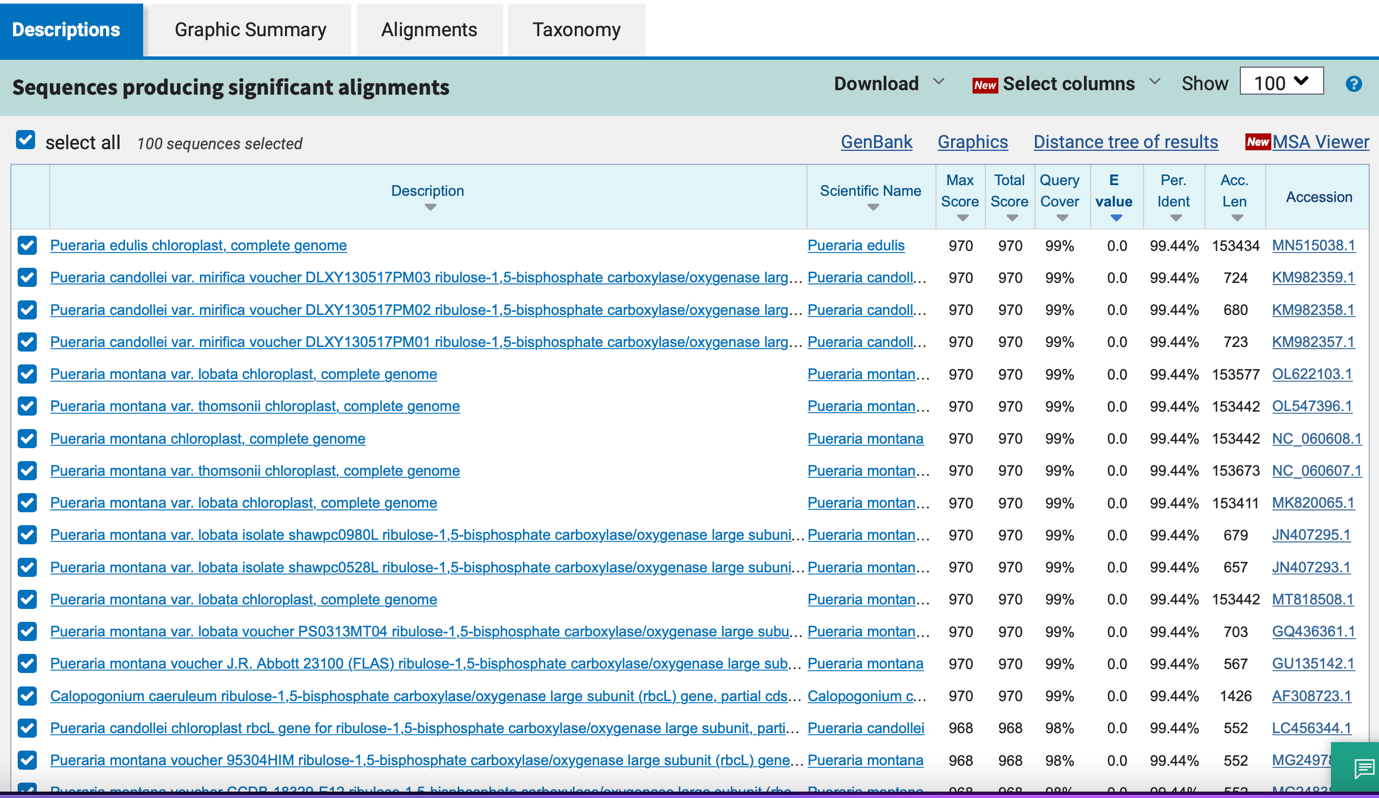


**8_** ***Senna alata***

ITS


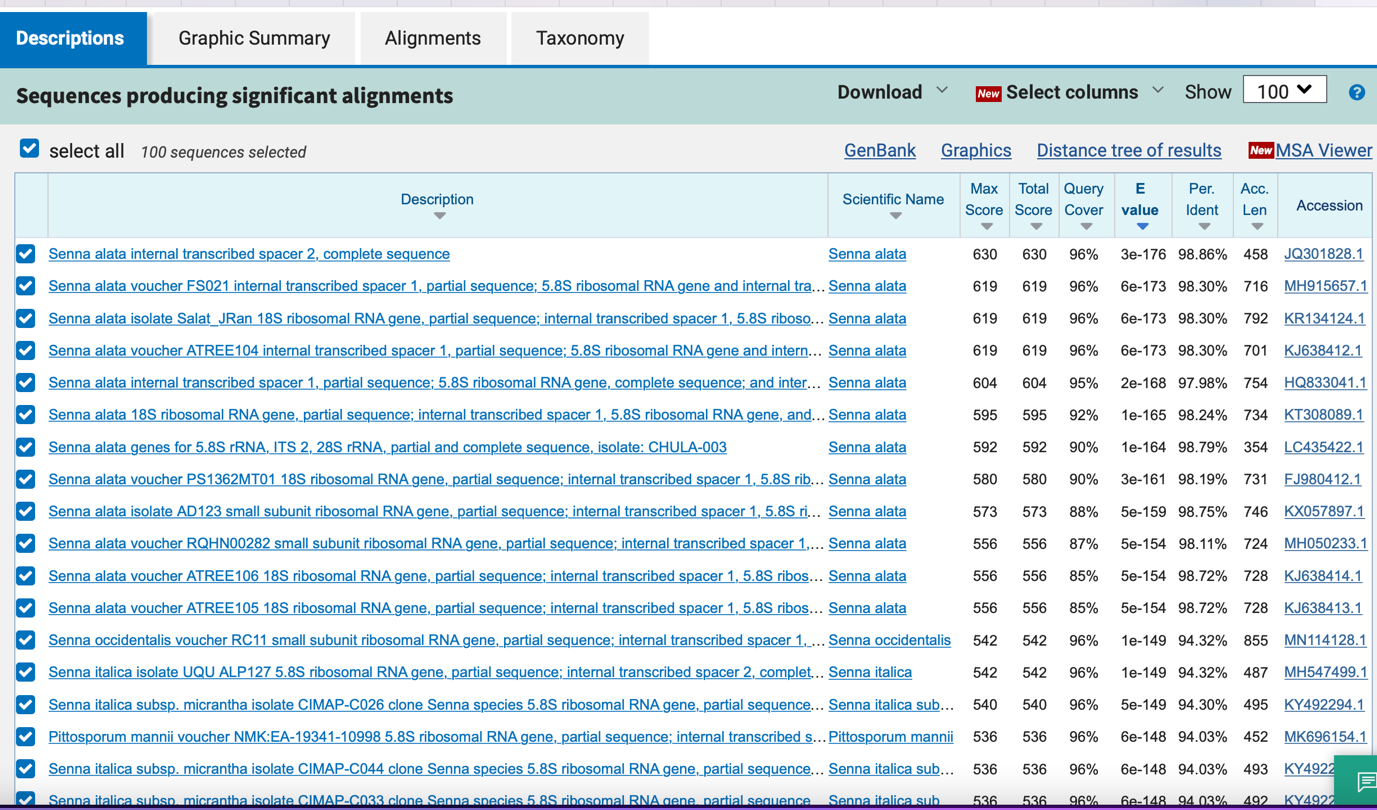


*mat*K


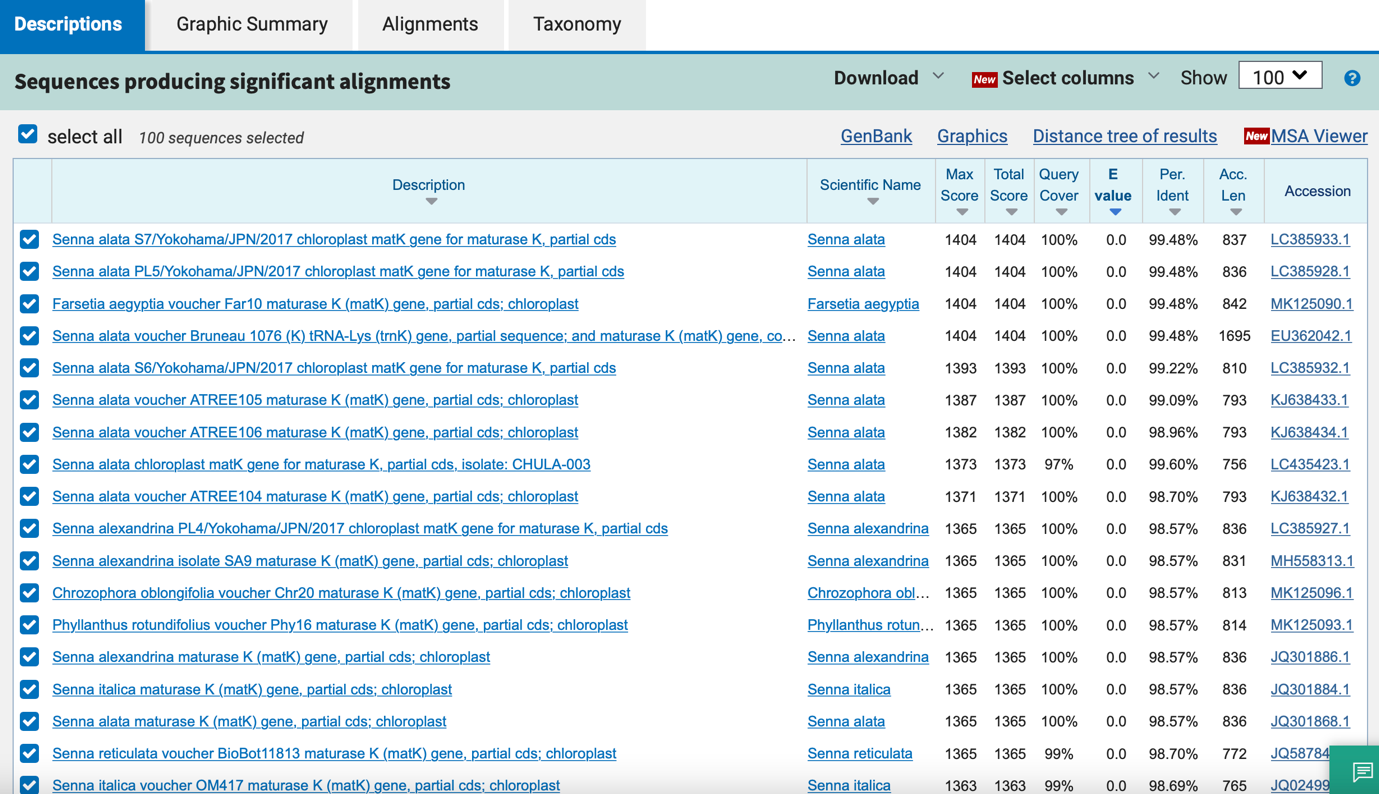


*psb*A_*trn*H


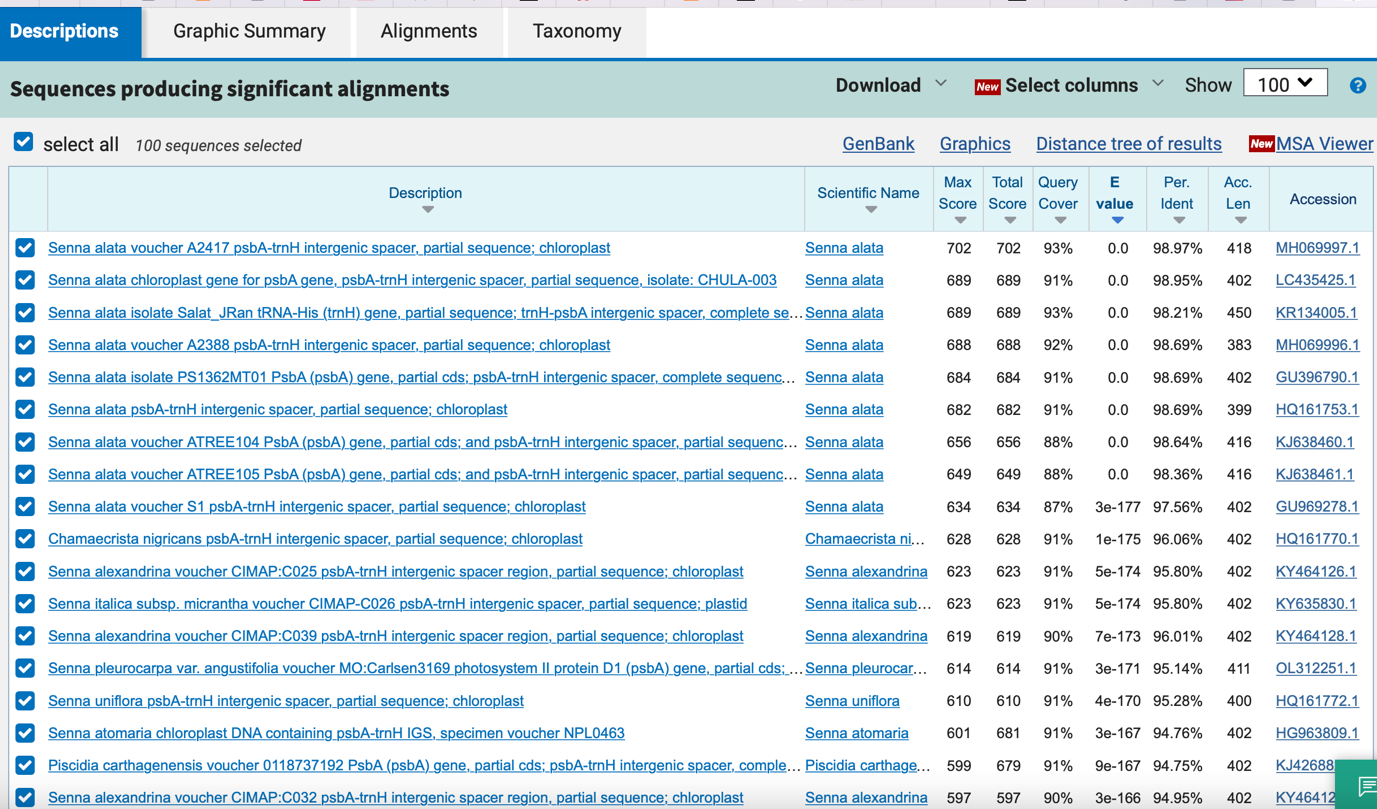


*rbc*L


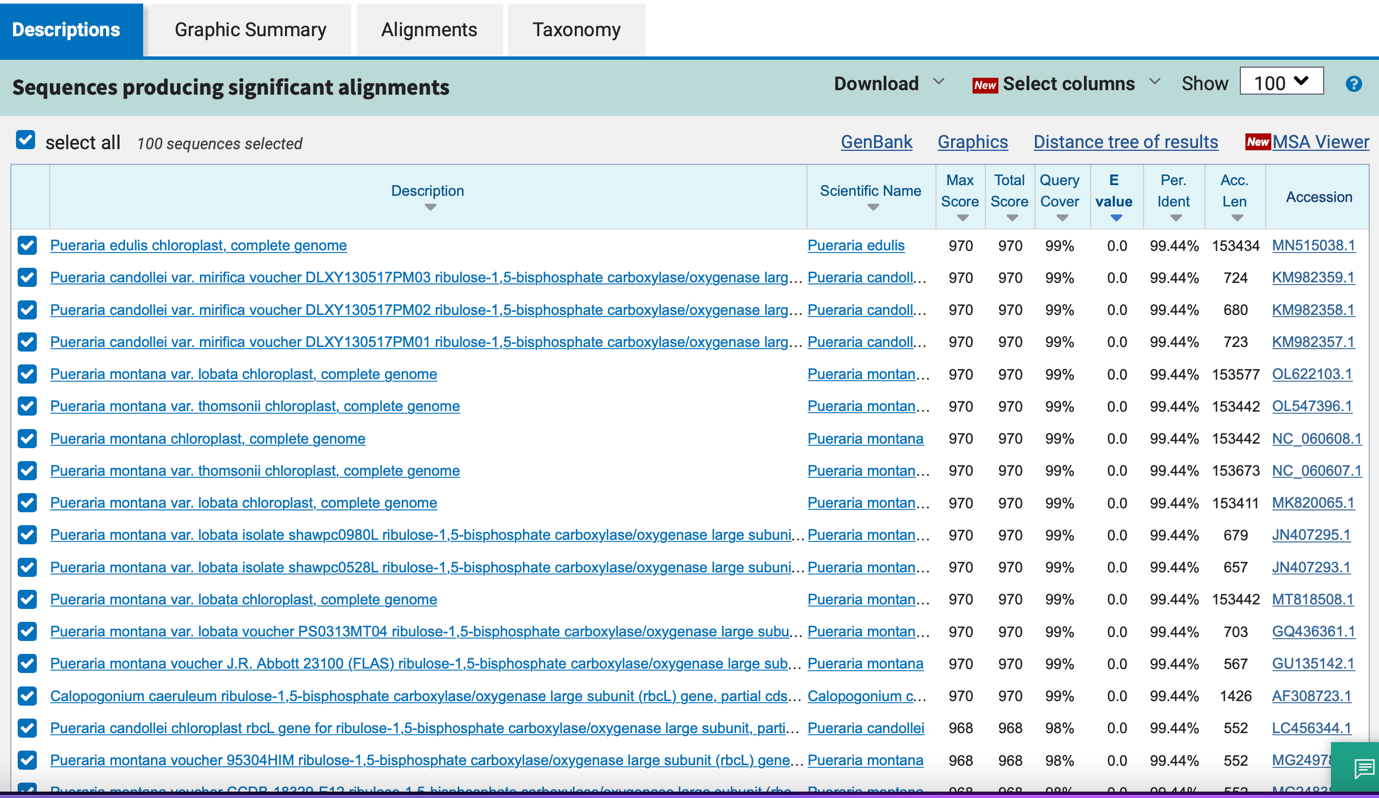


**9_** ***Boesenbergia rotunda***

ITS


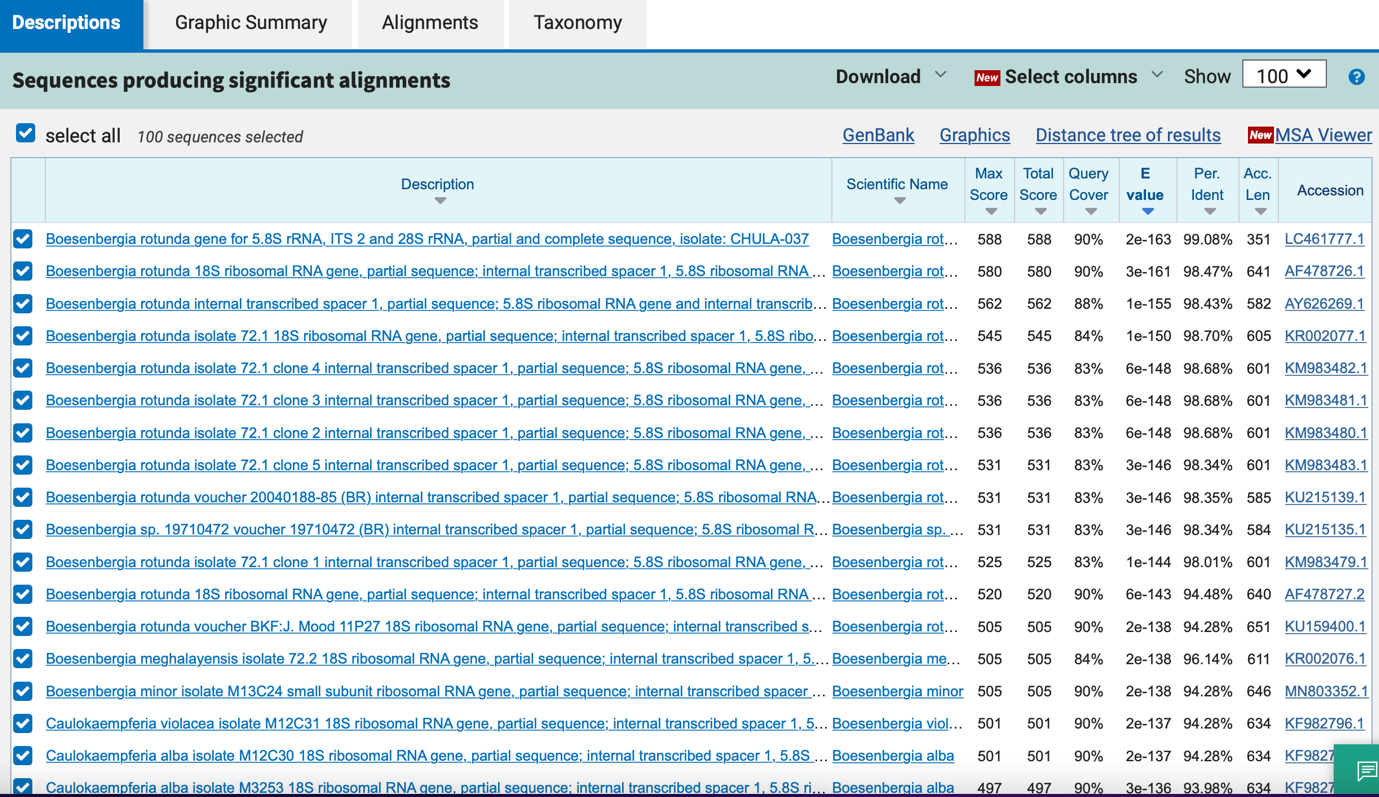


*mat*K


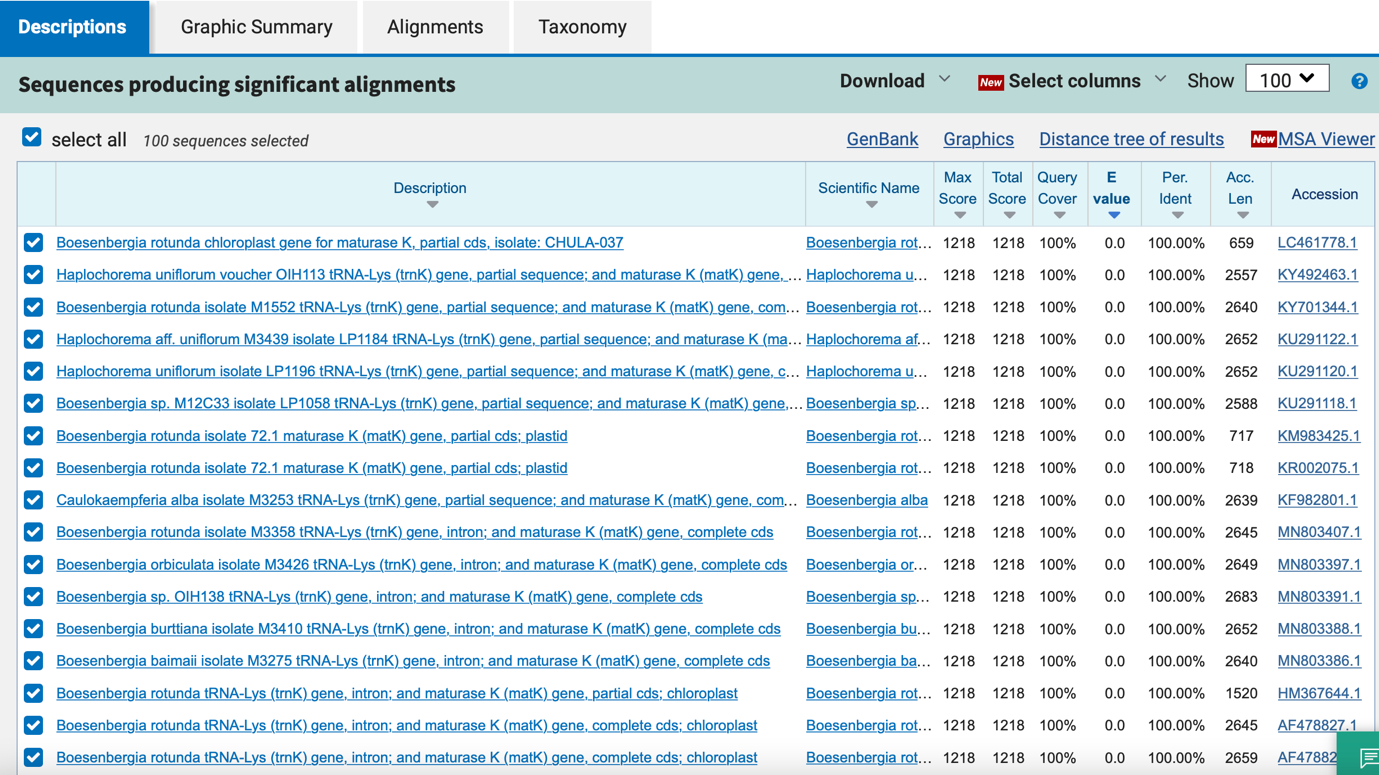


*psb*A_*trn*H

*
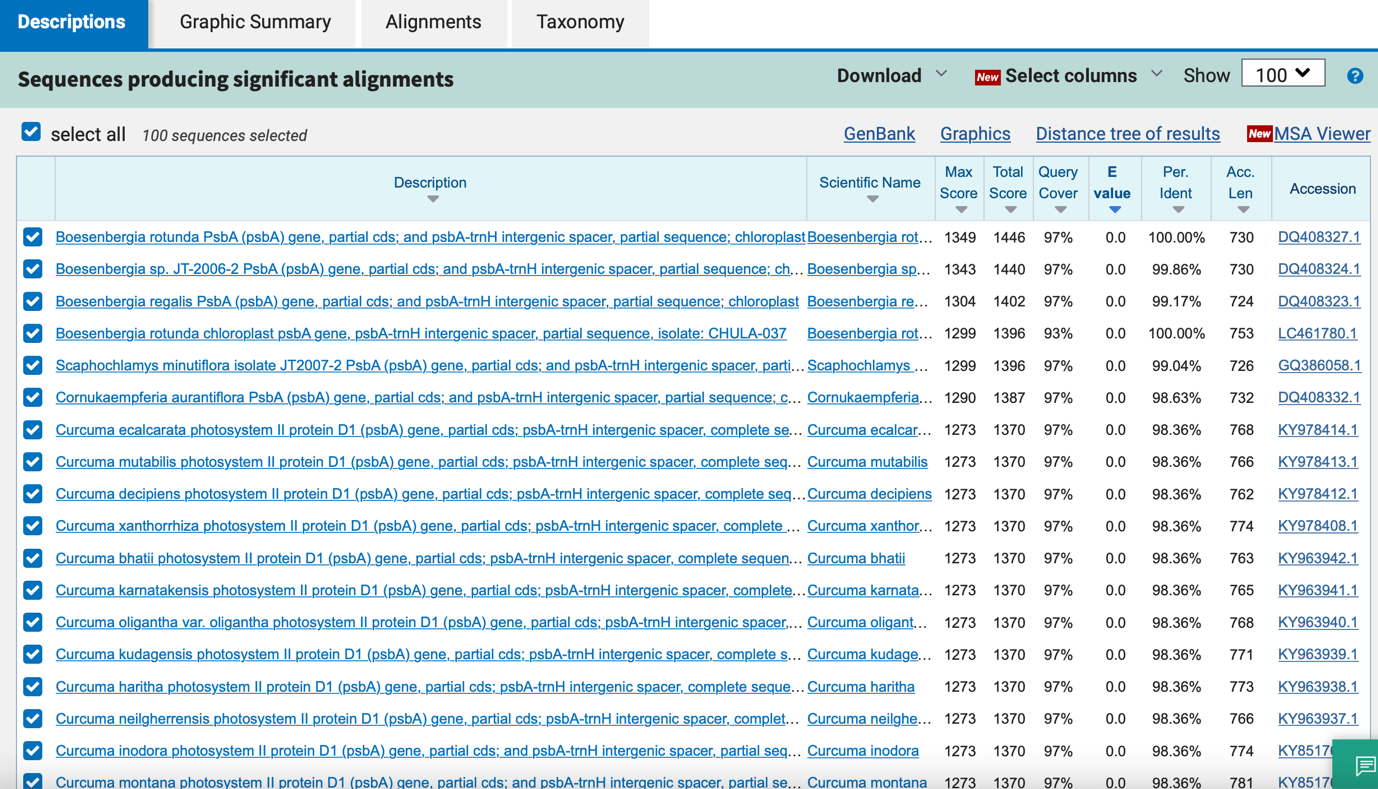
*

*rbc*L


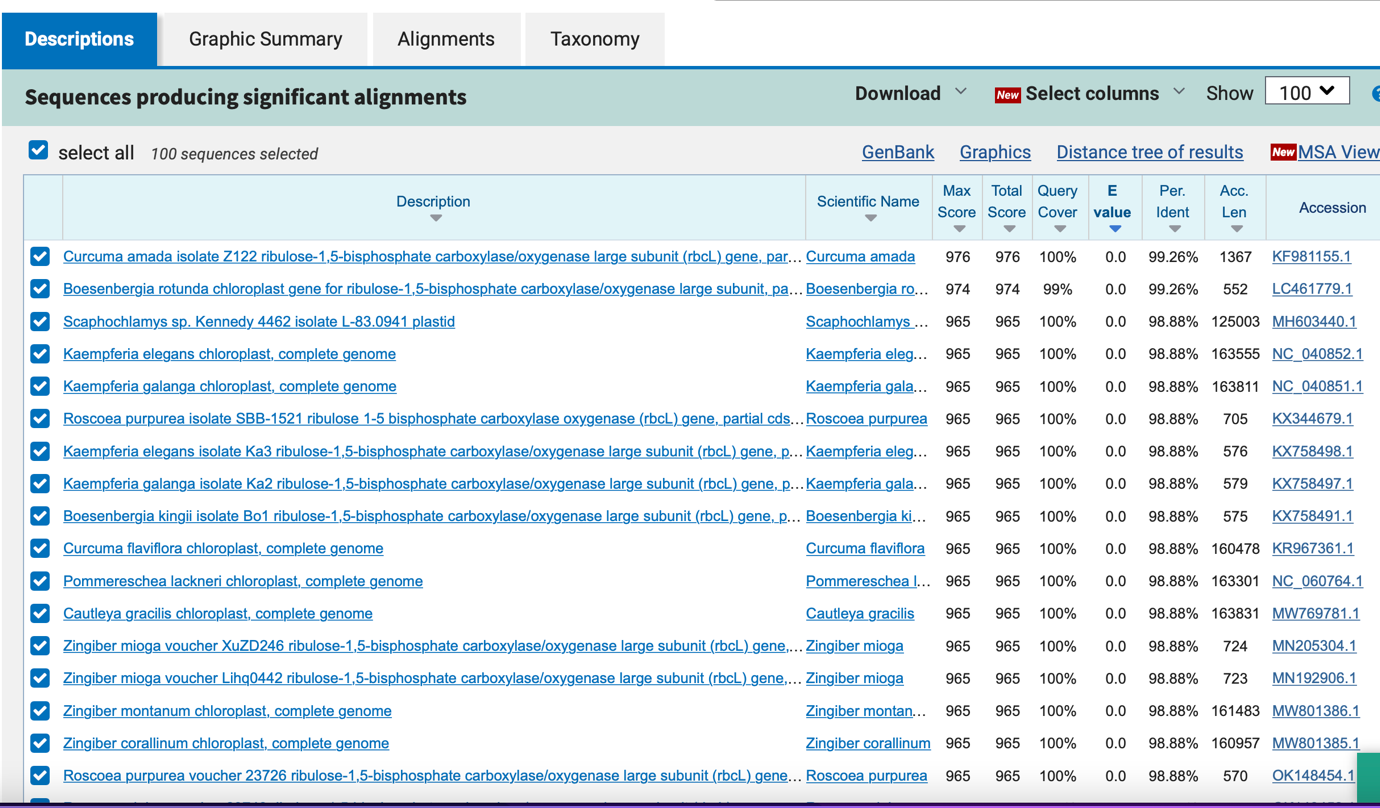


**10_** ***Clinacanthus nutans***

ITS


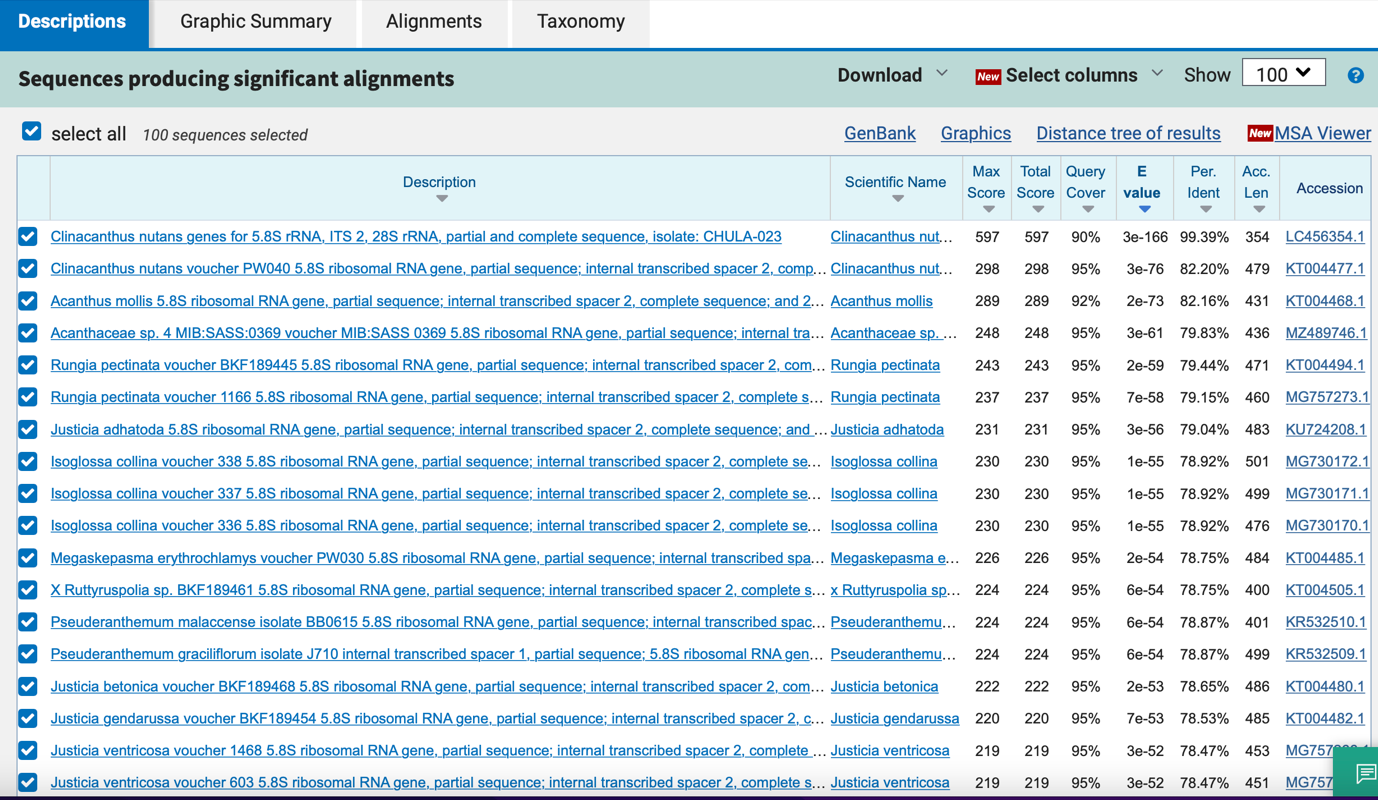


*mat*K


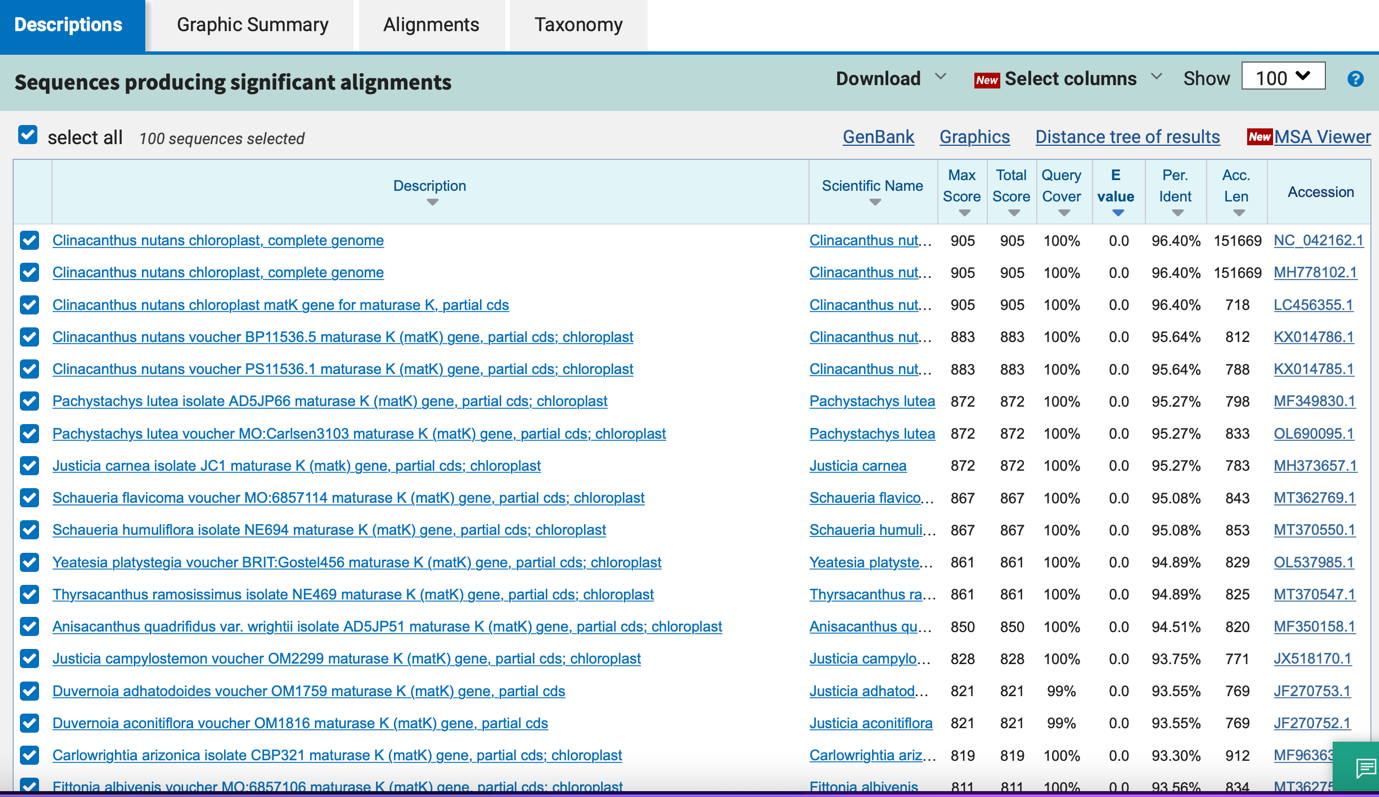


*psb*A_*trn*H


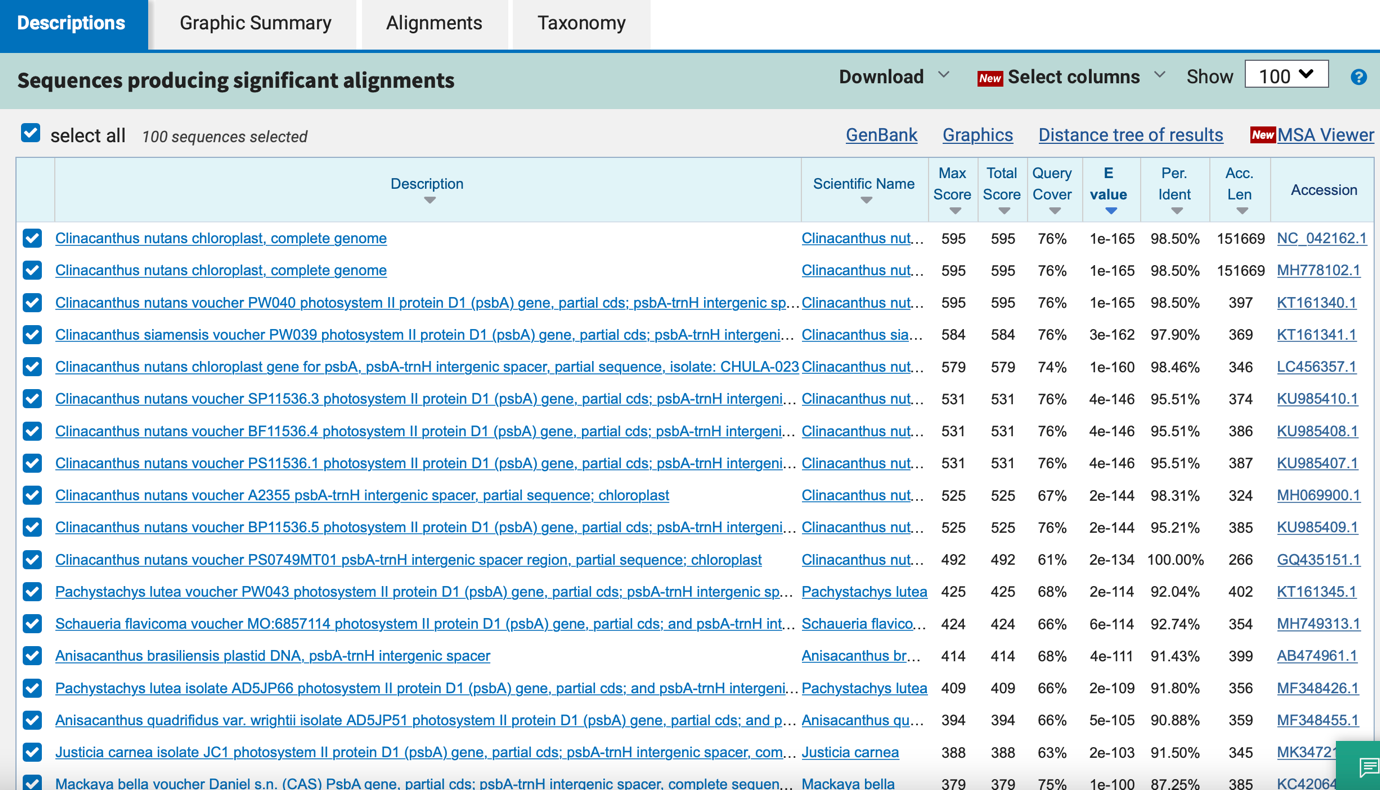


*rbc*L


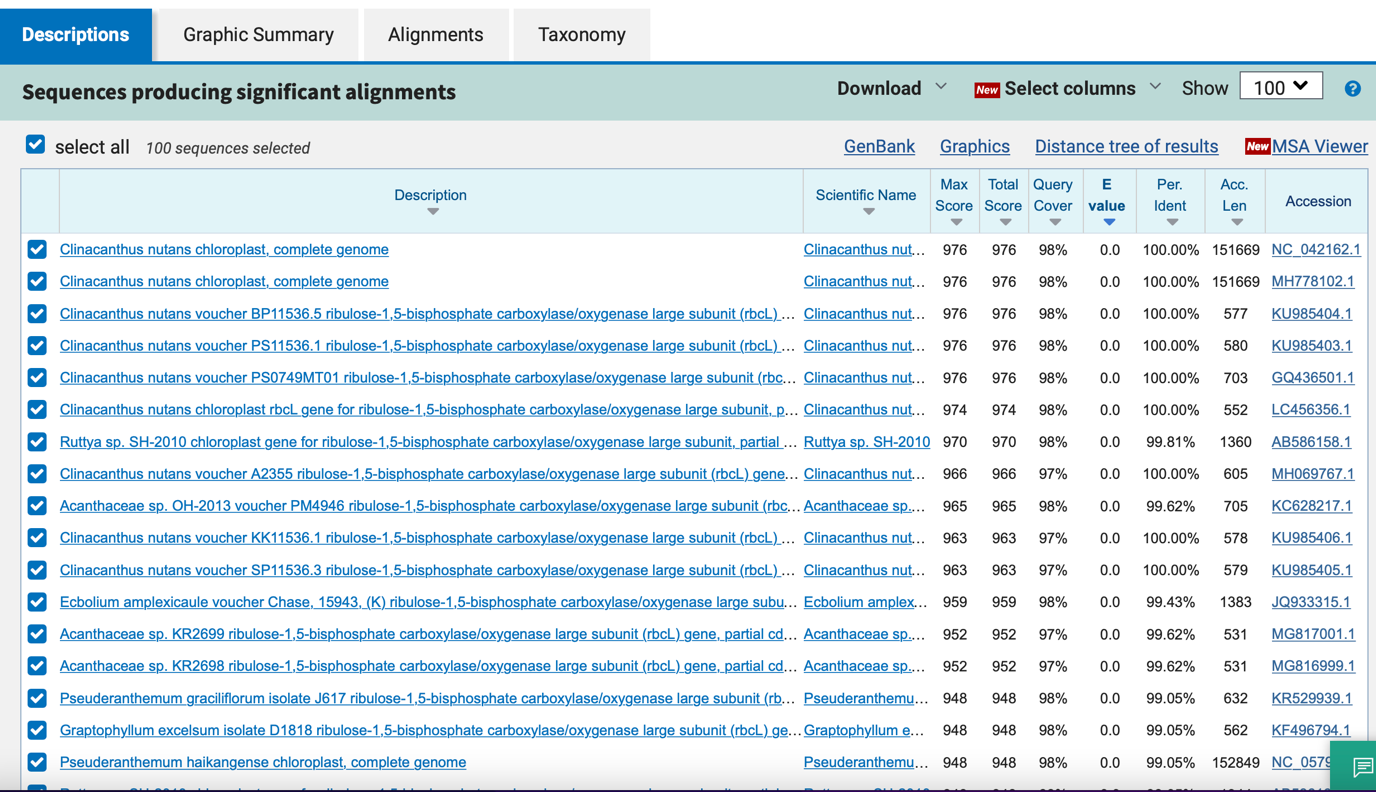


**11_** ***Curcuma longa***

ITS


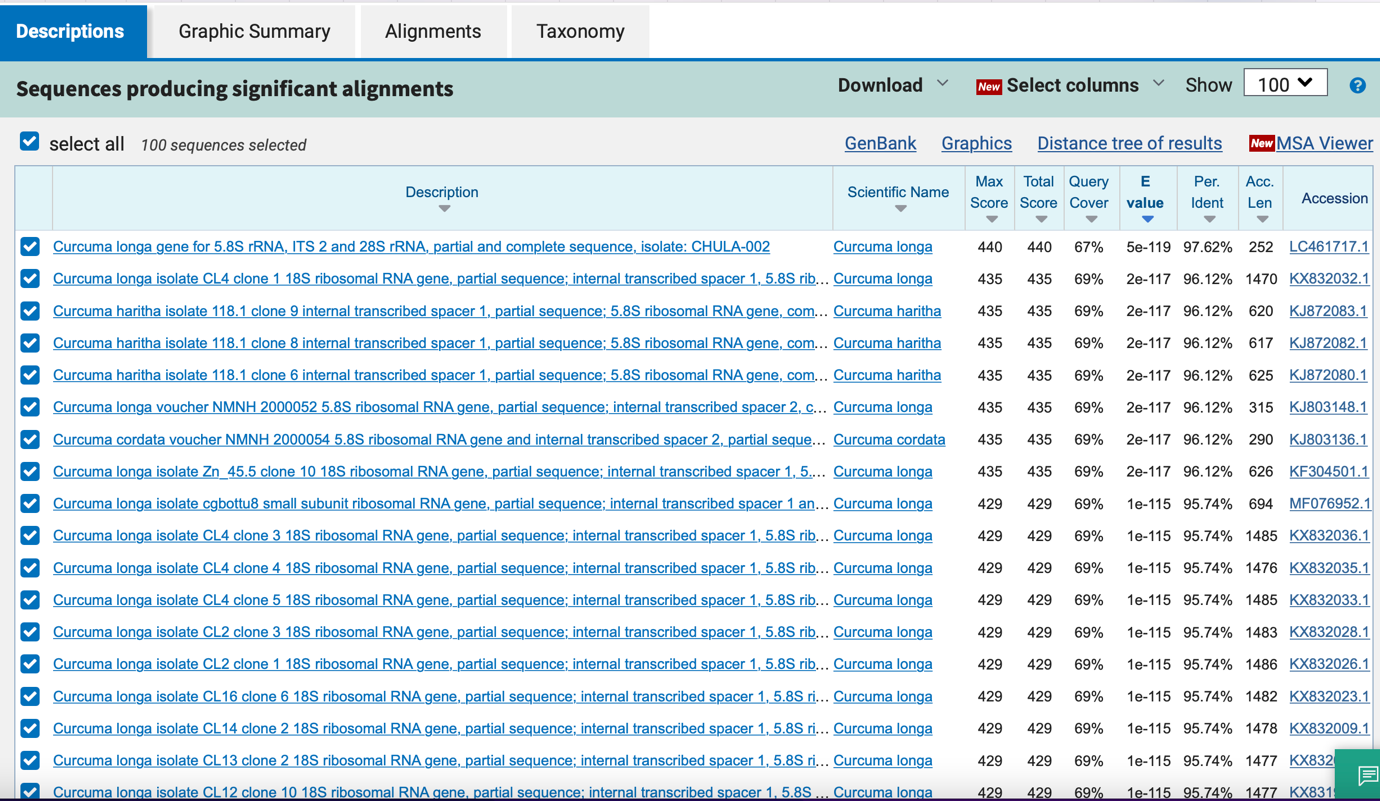


*mat*K

***
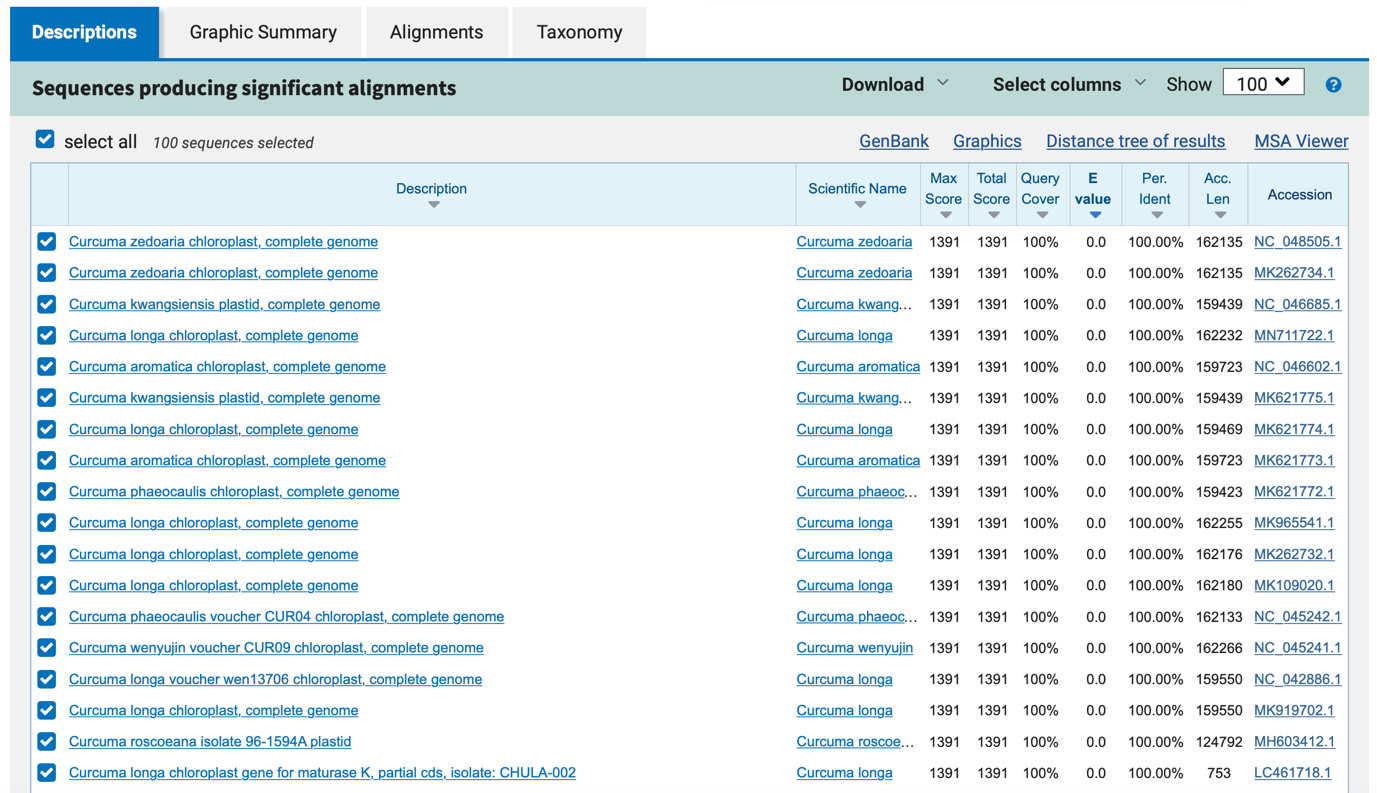
***

*psb*A_*trn*H


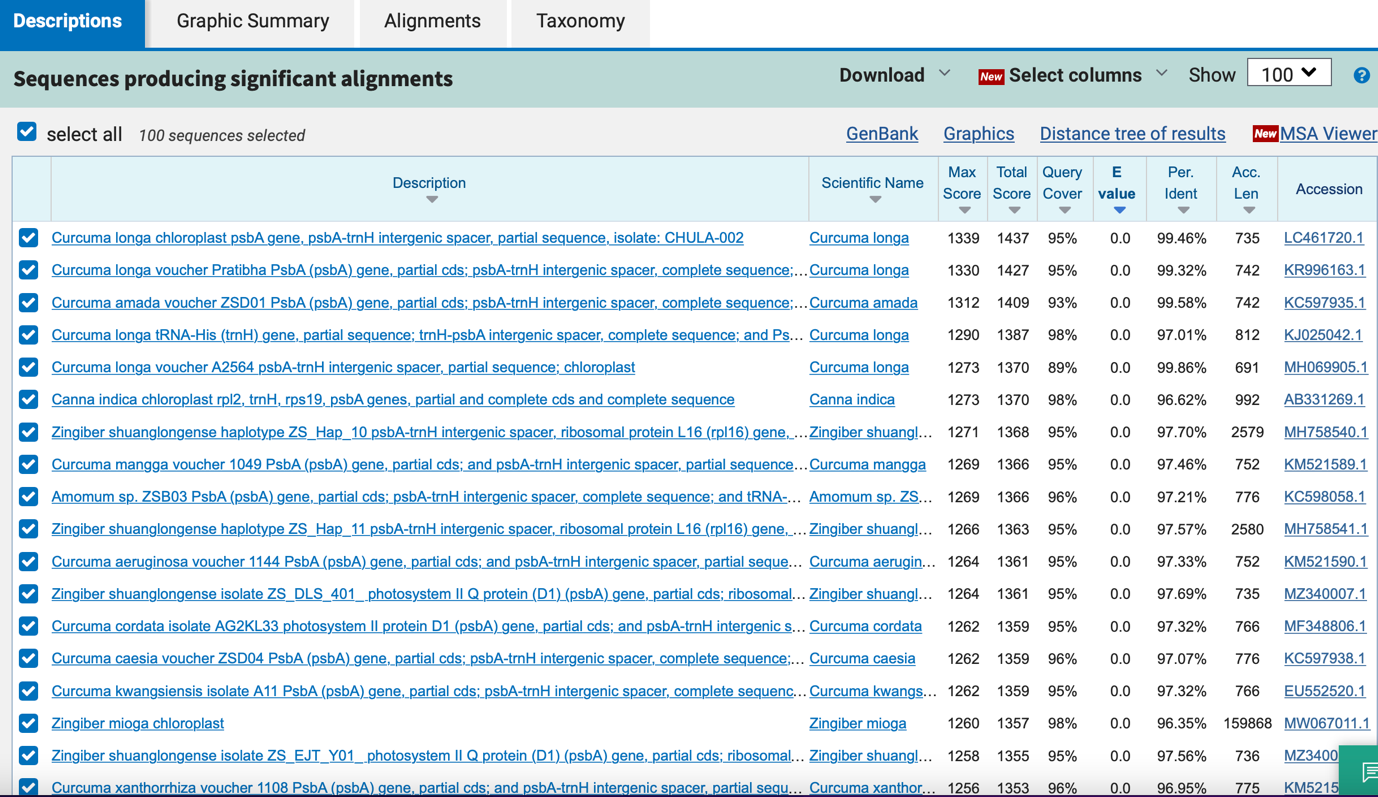


*rbc*L


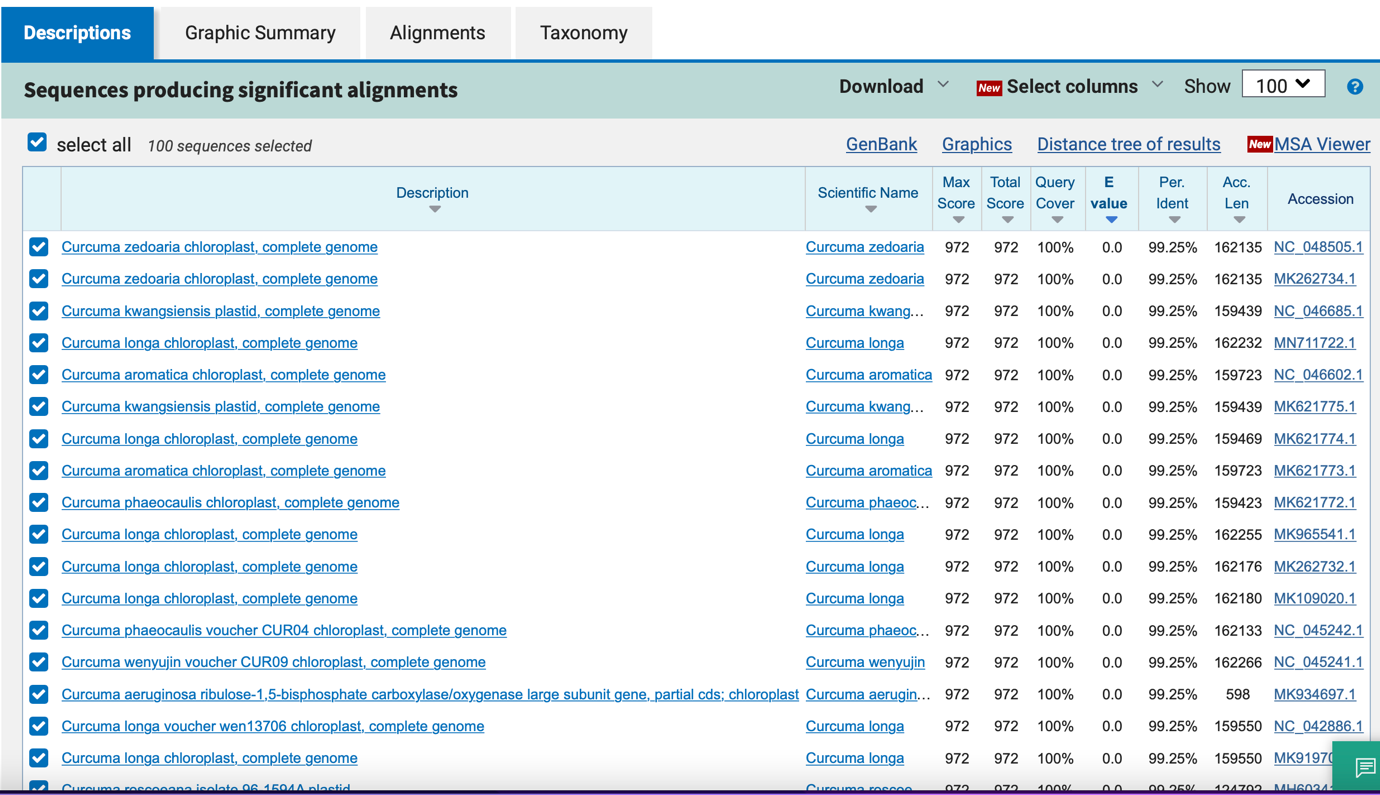


**12_** ***Centella asiatica***

ITS


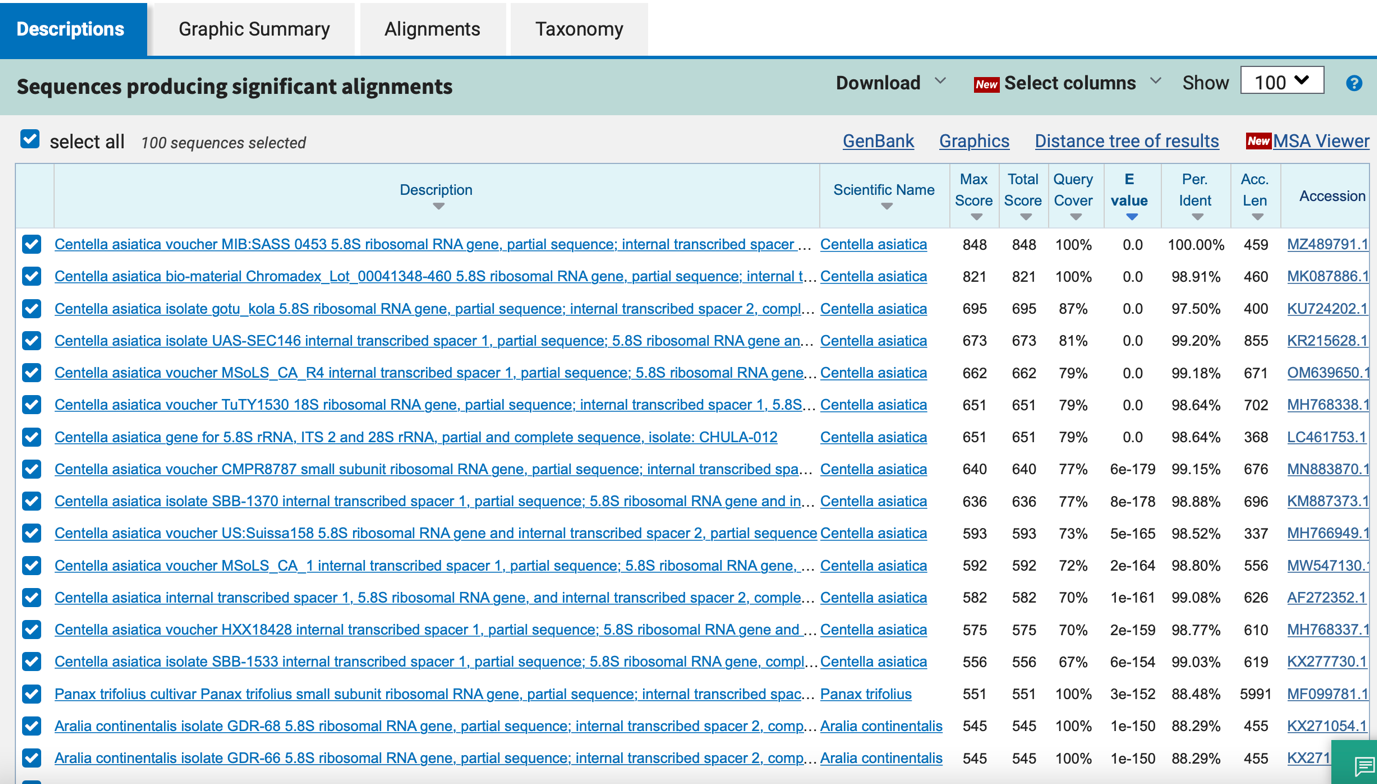


*mat*K


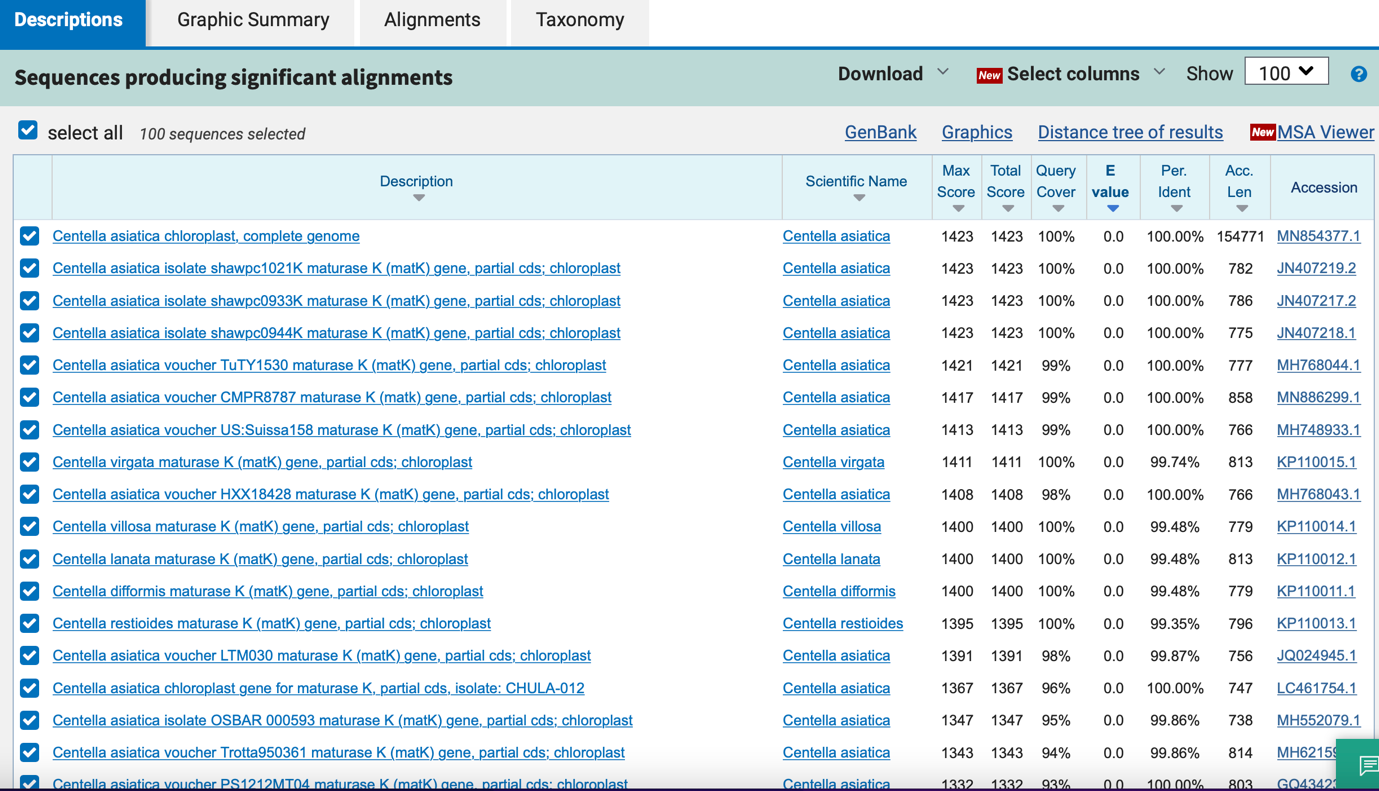


*psb*A_*trn*H


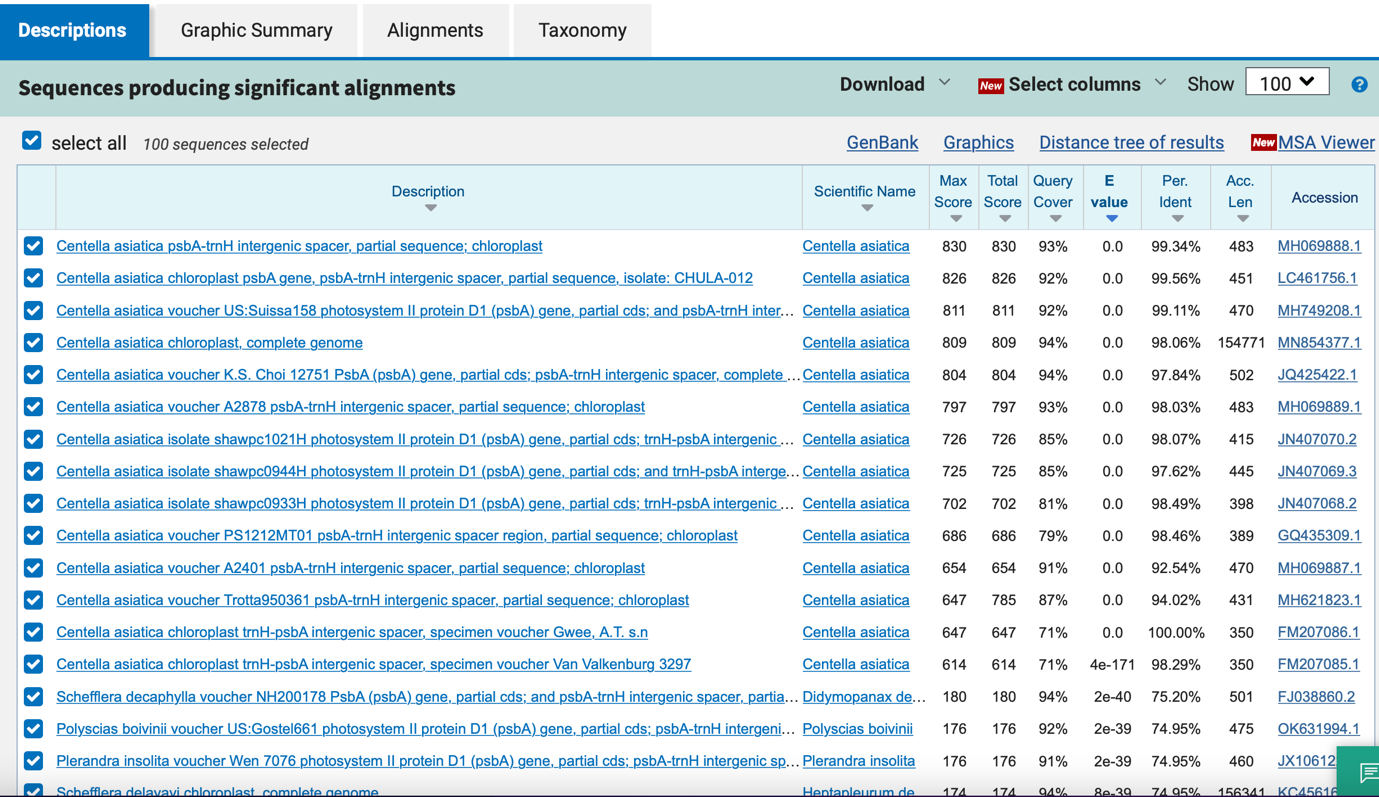


*rbc*L


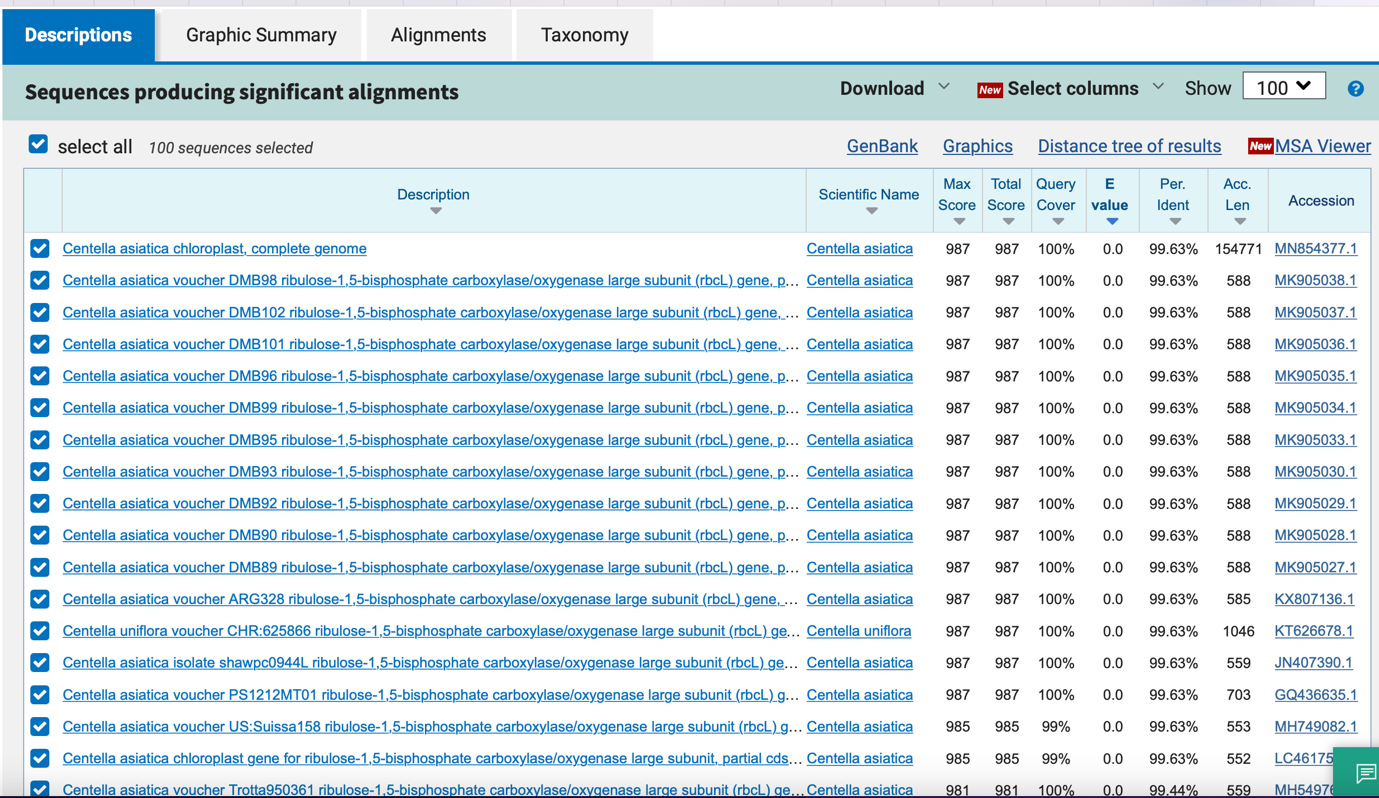


**13_** ***Pueraria candollei butea***

ITS


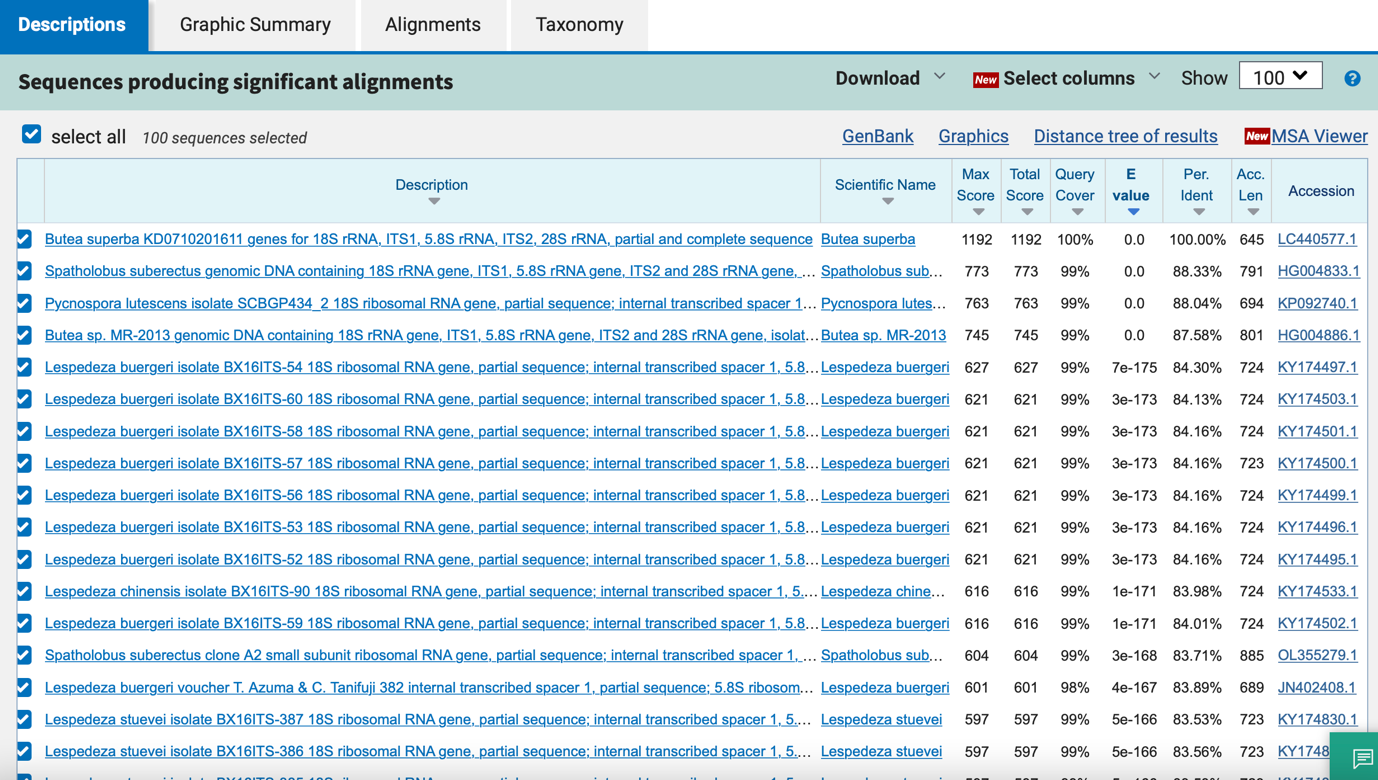


*mat*K


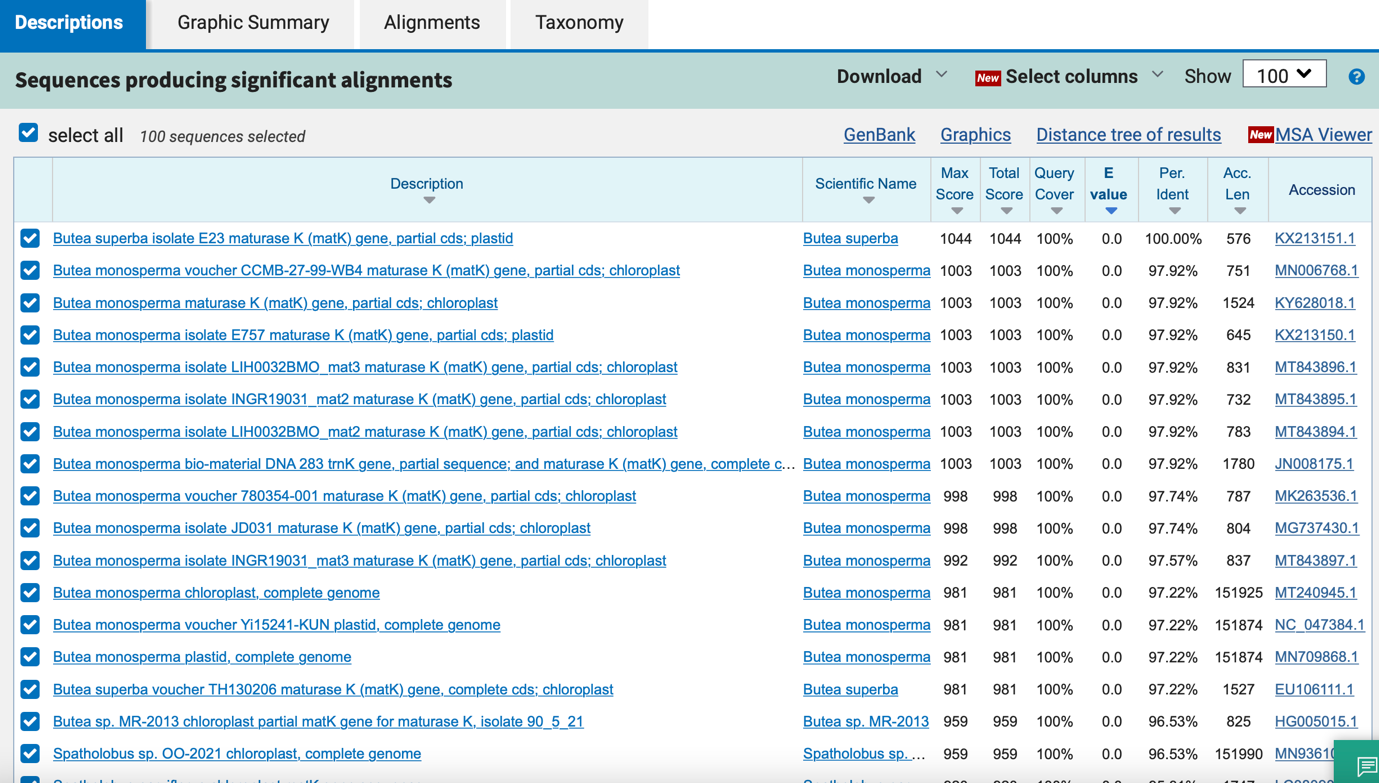


*psb*A_*trn*H


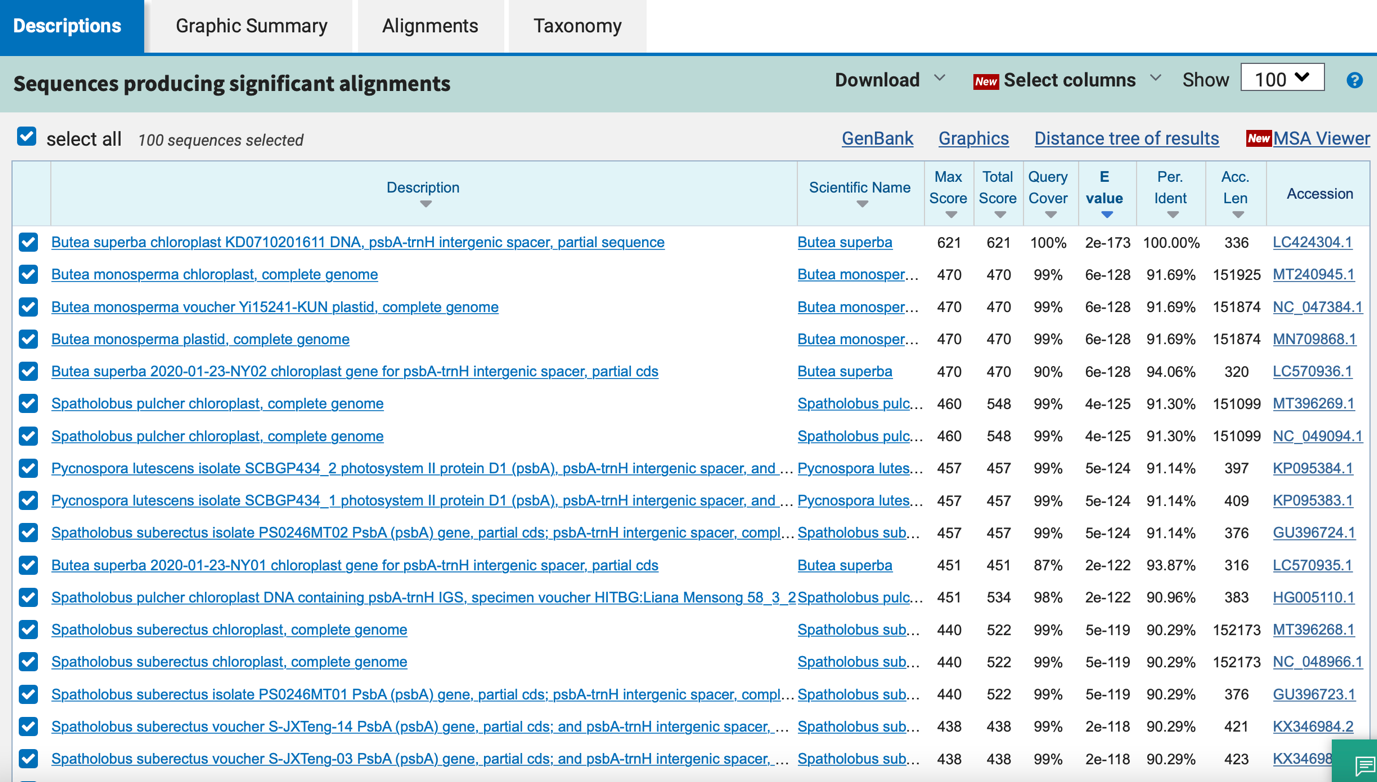


*rbc*L


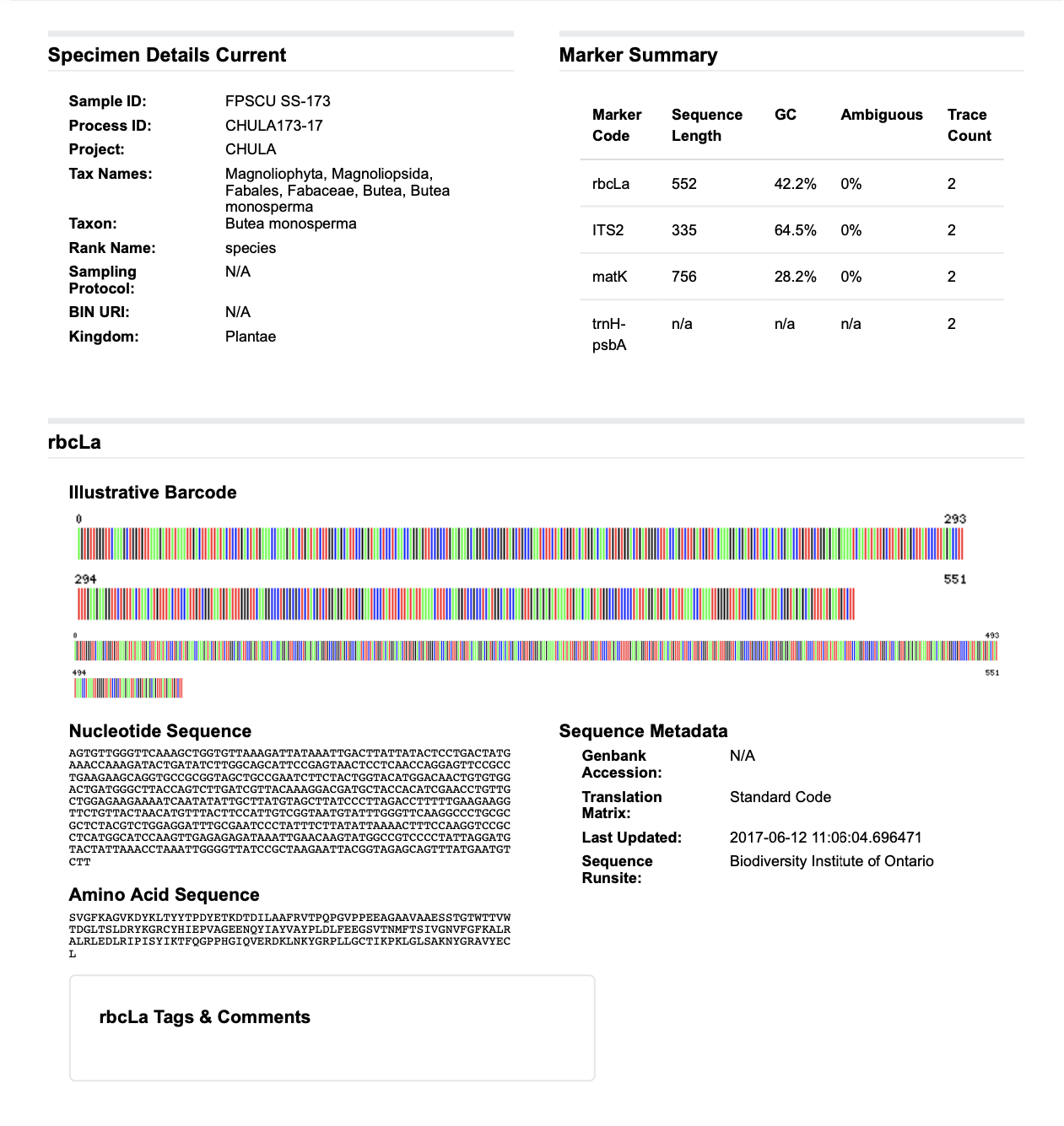


**14_** ***Centella asiatica***

ITS


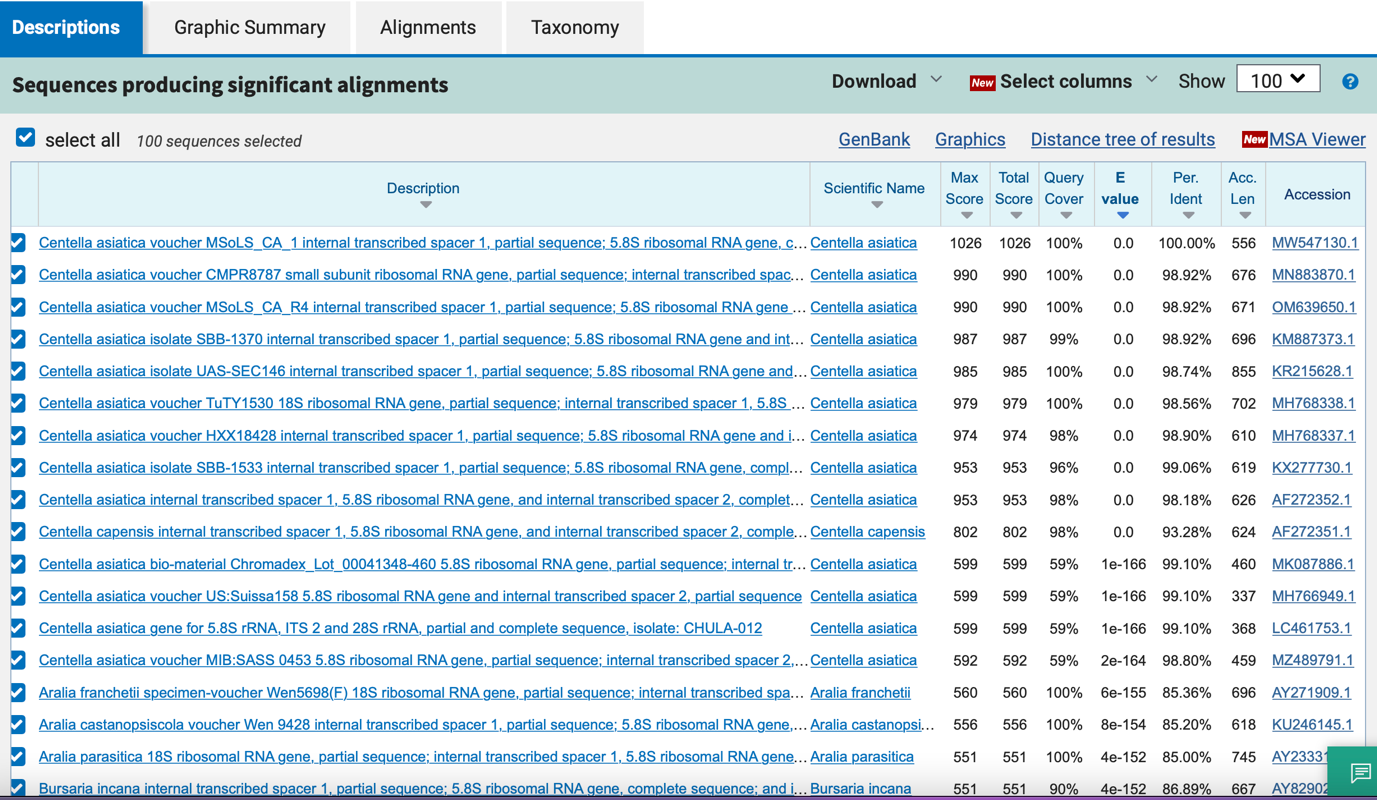


*mat*K


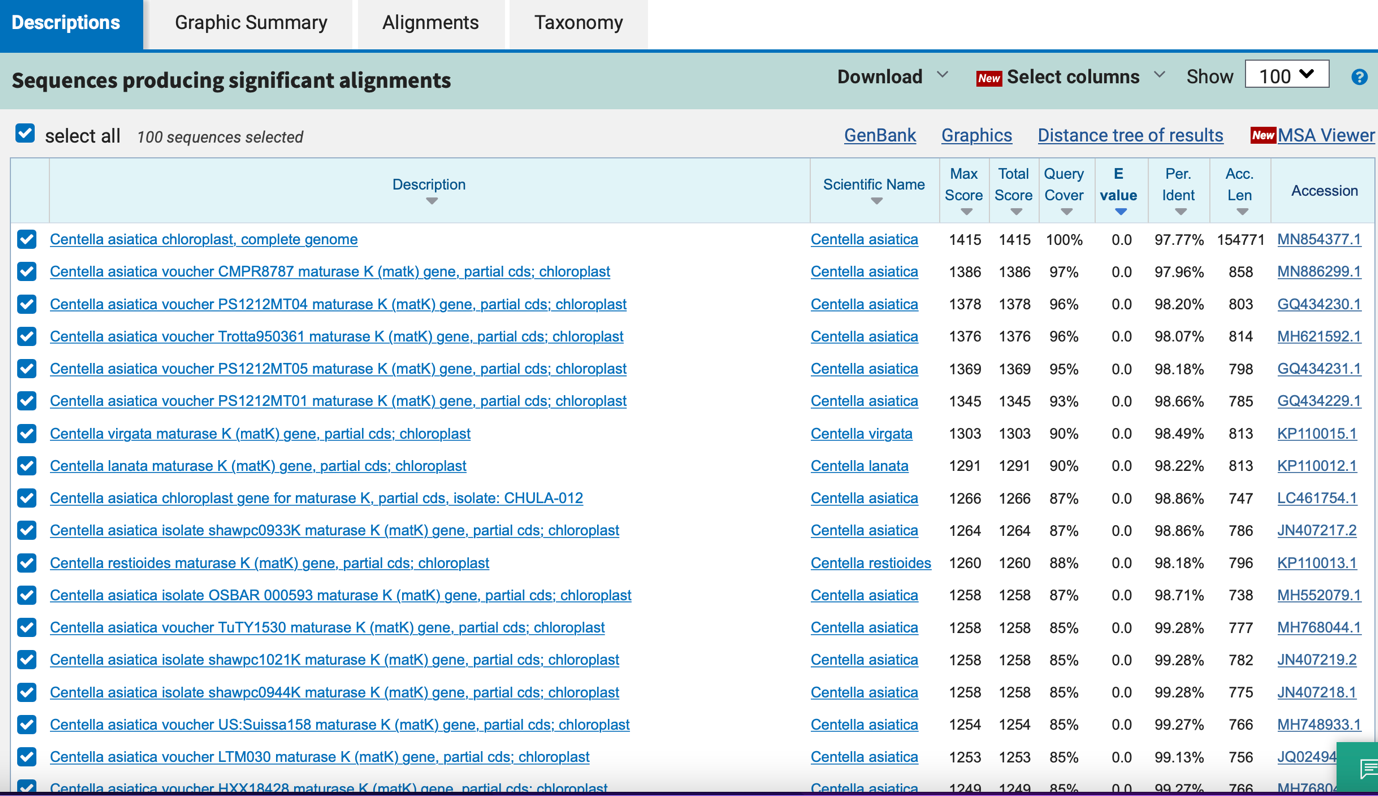


*psb*A_*trn*H


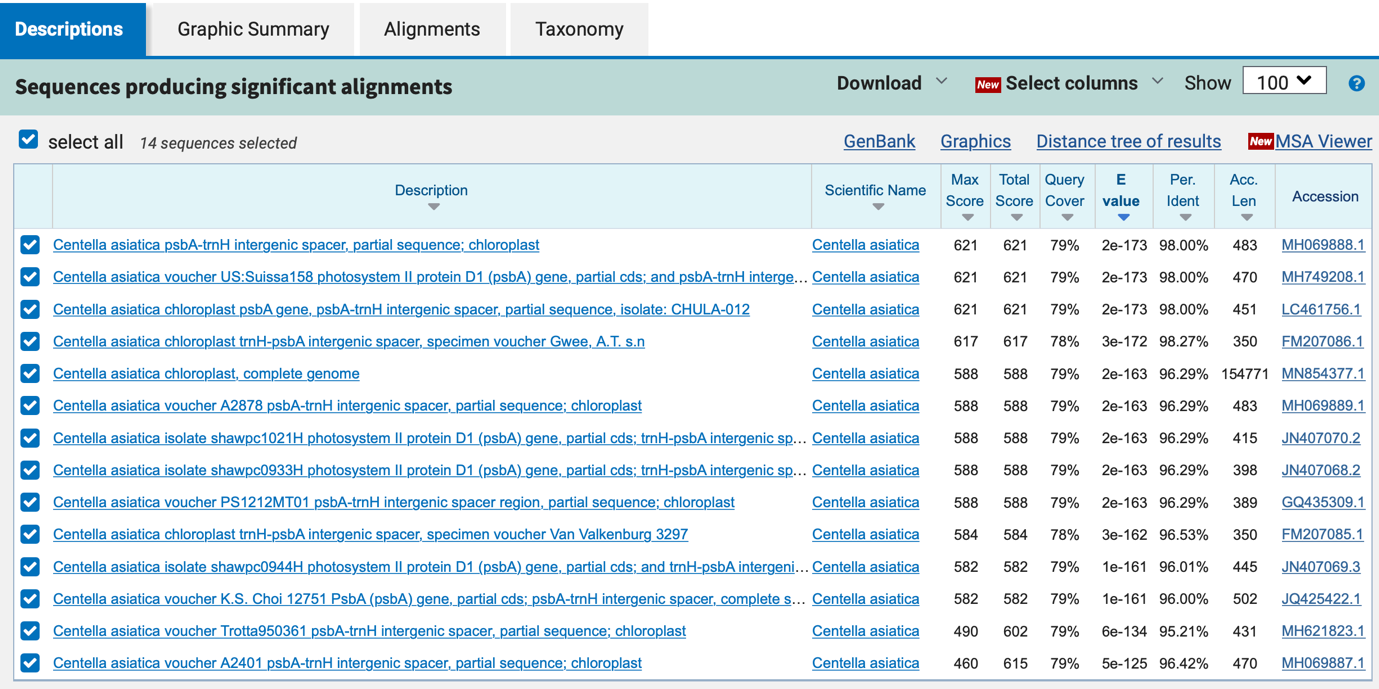


*rbc*L


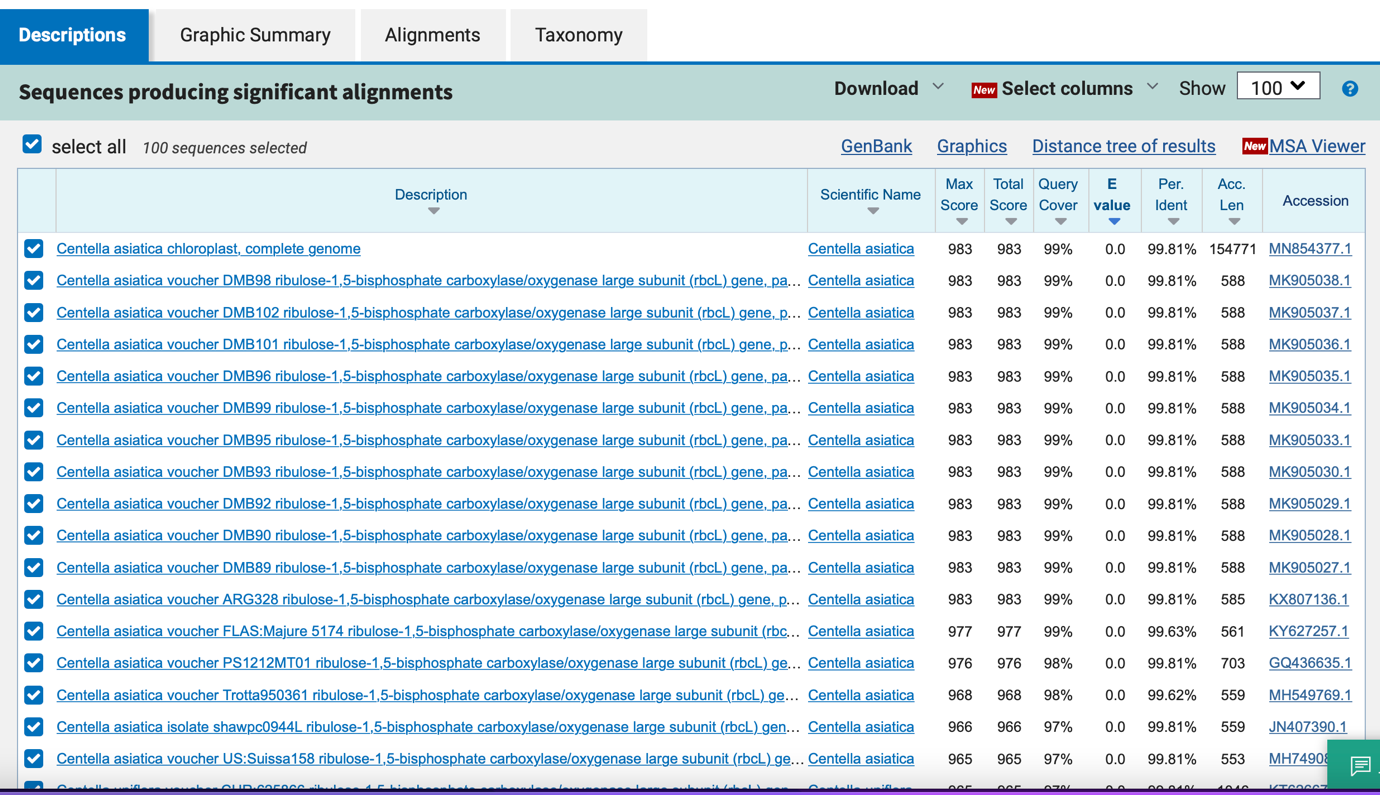


**15_** ***Curcuma longa***

ITS


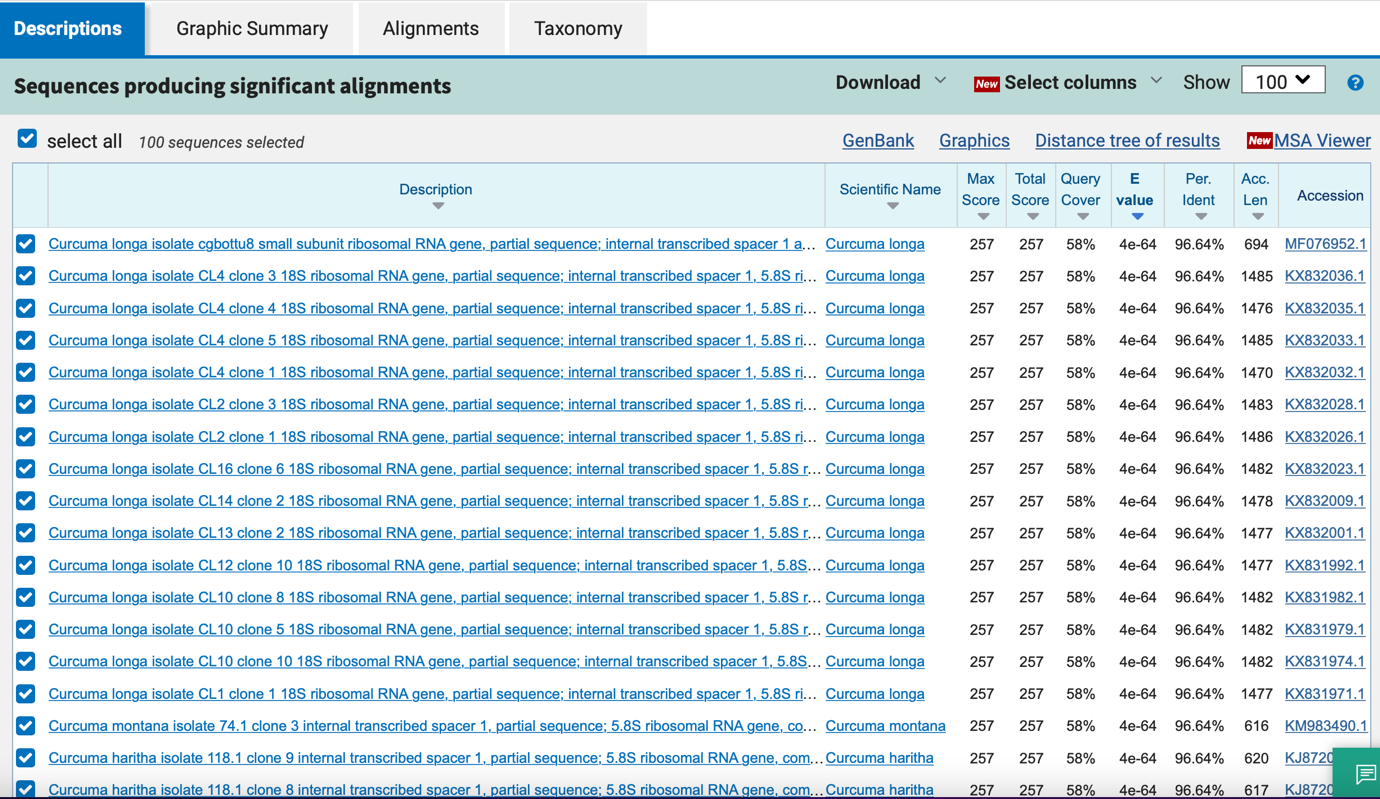


*mat*K

*
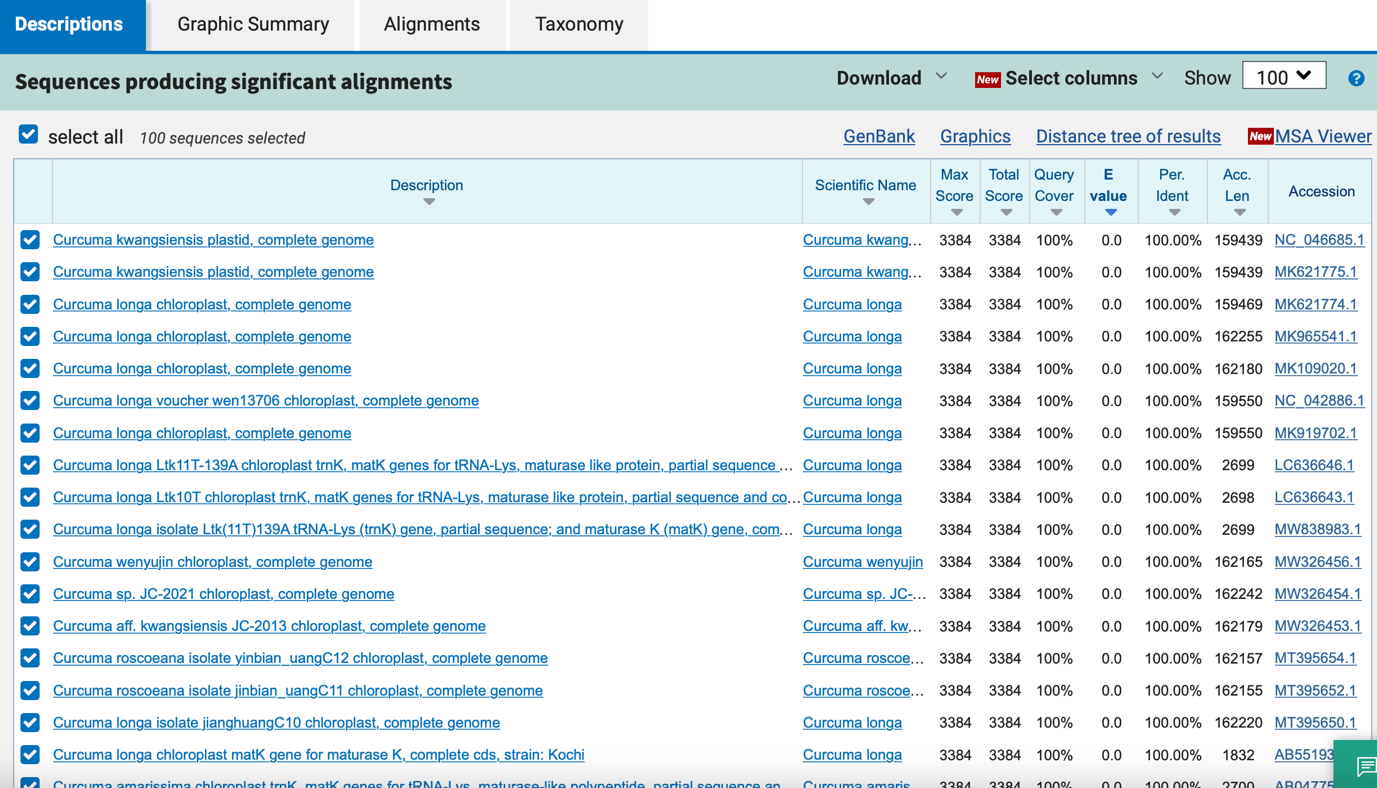
*

*psb*A_*trn*H


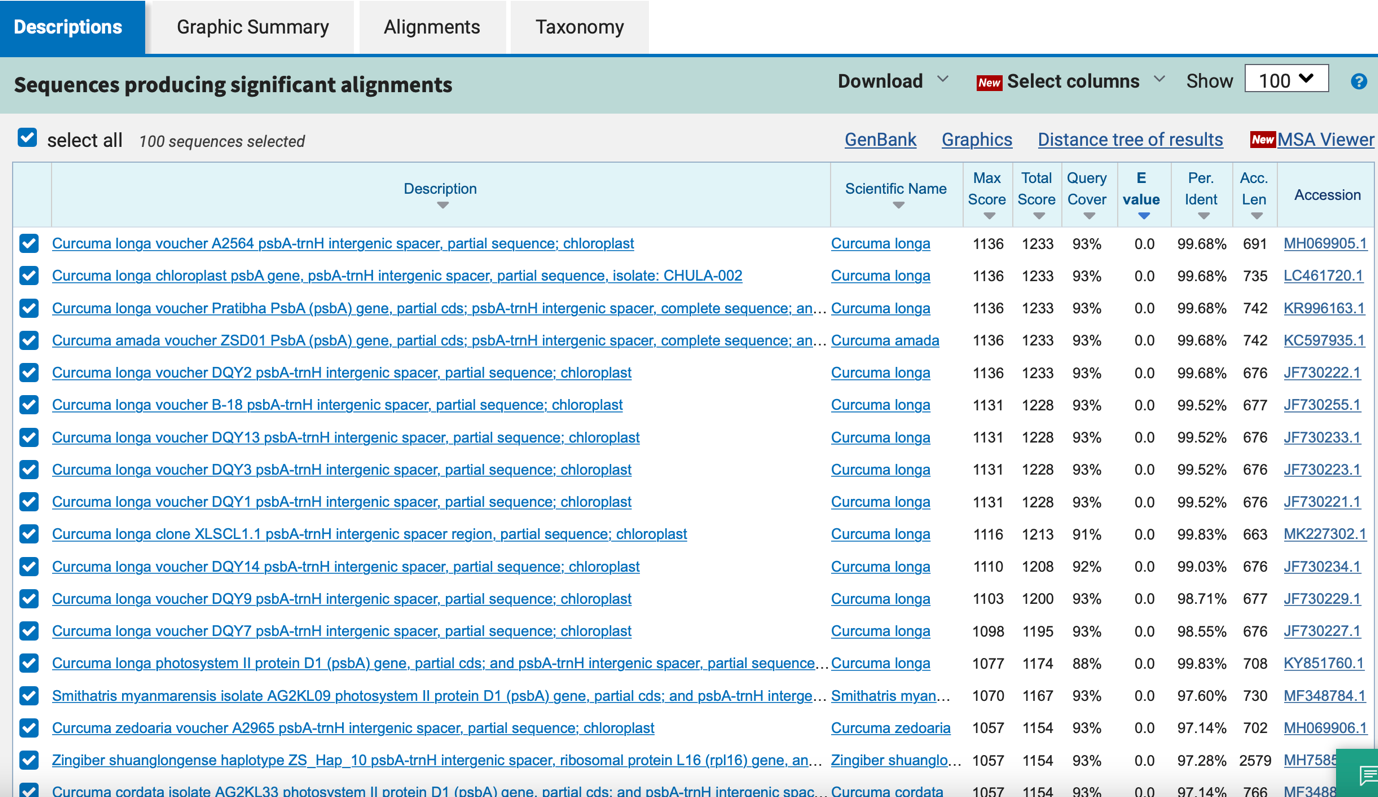


*rbc*L


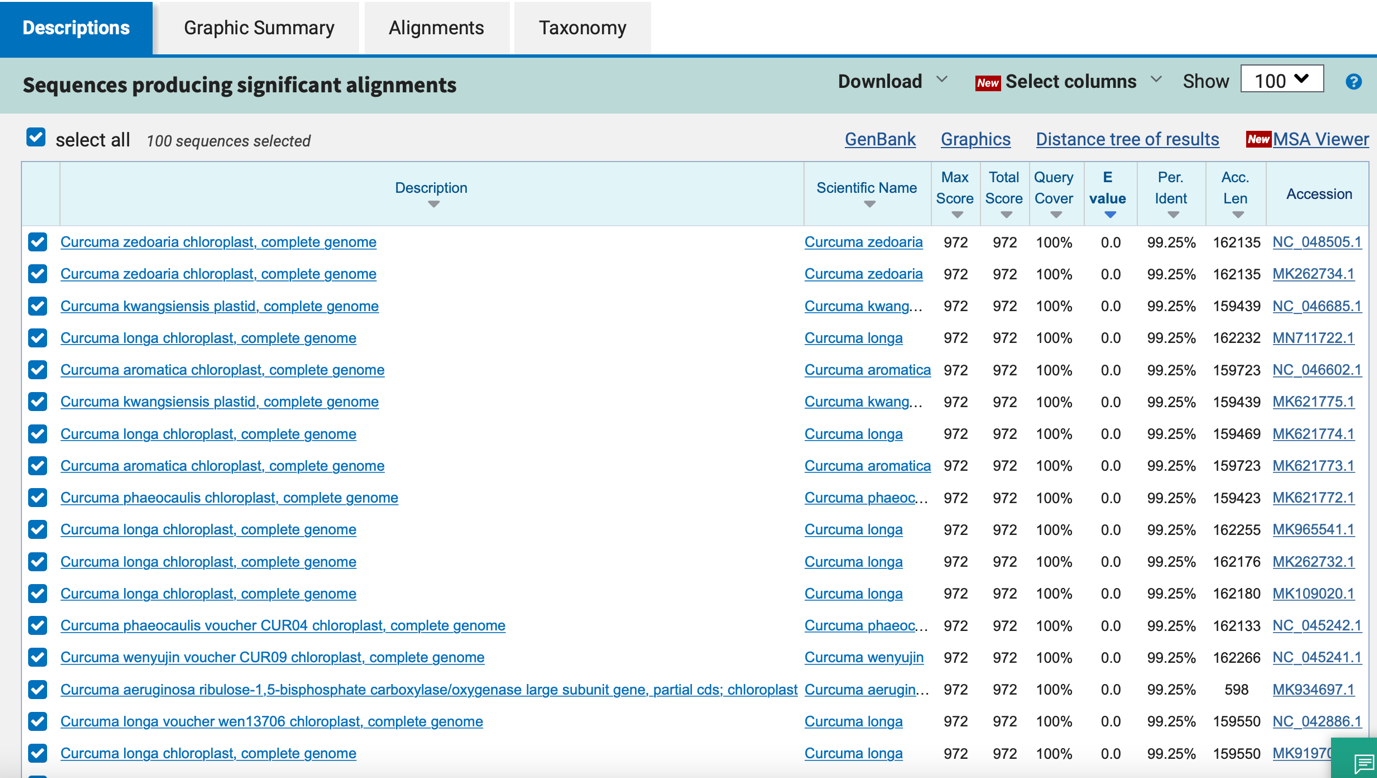


**16_** ***Kaempferia parviflora***

ITS


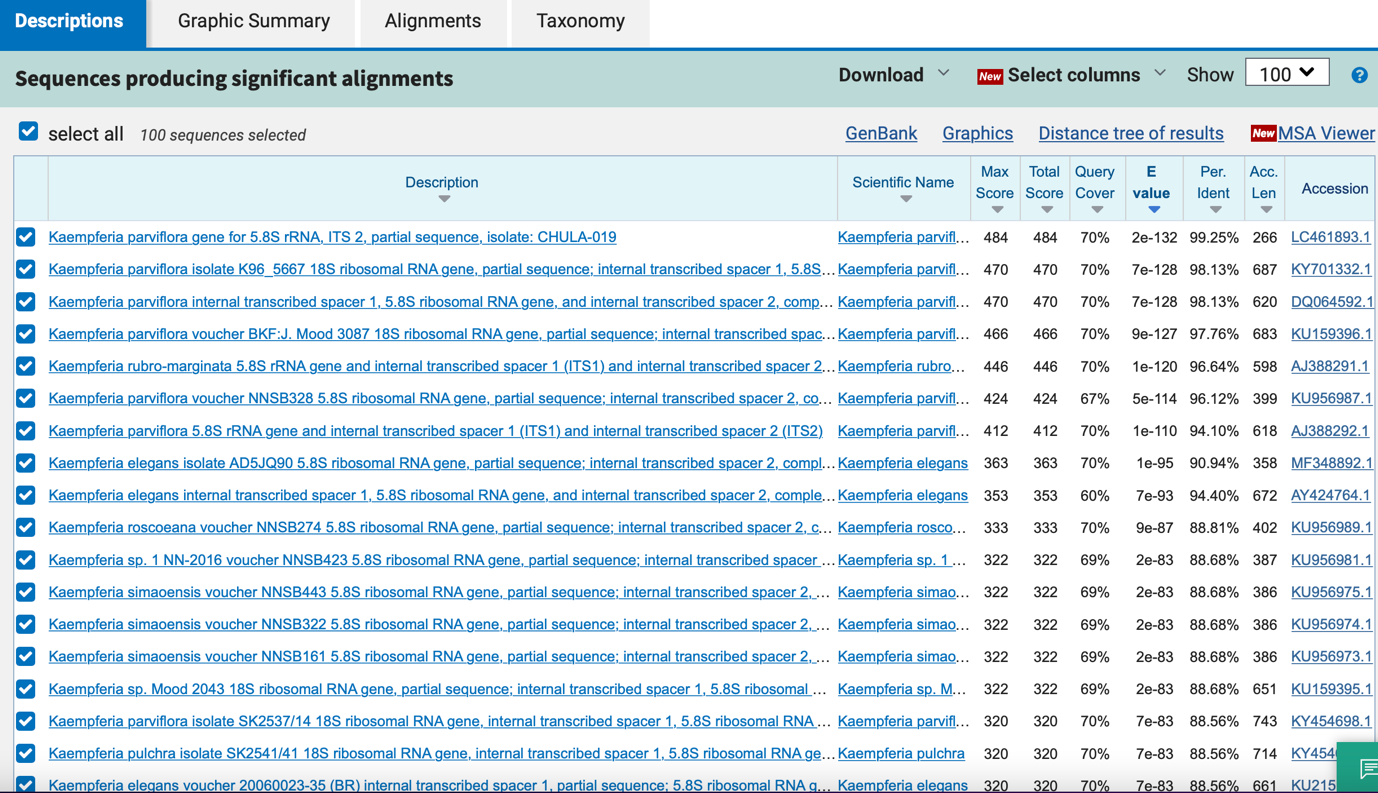


*mat*K


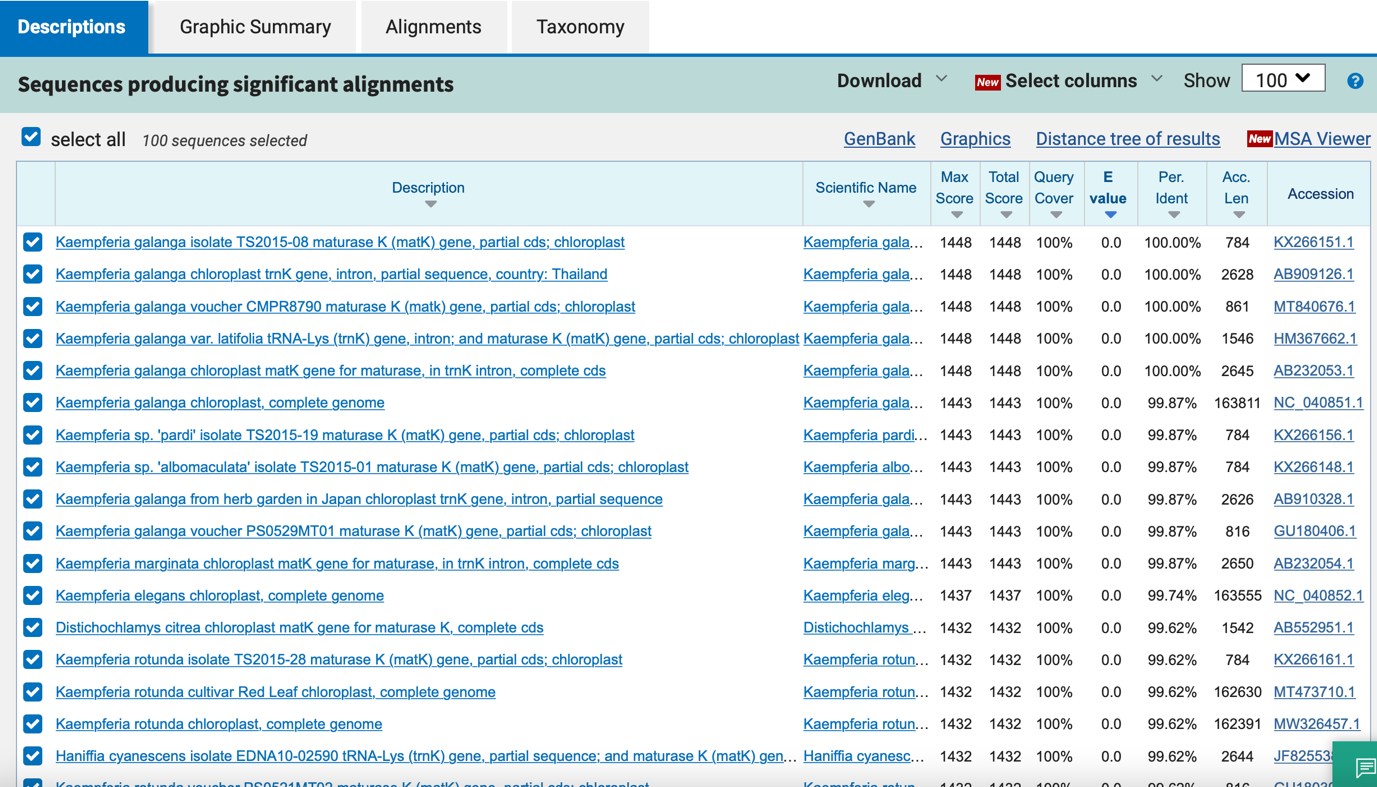


*psb*A_*trn*H


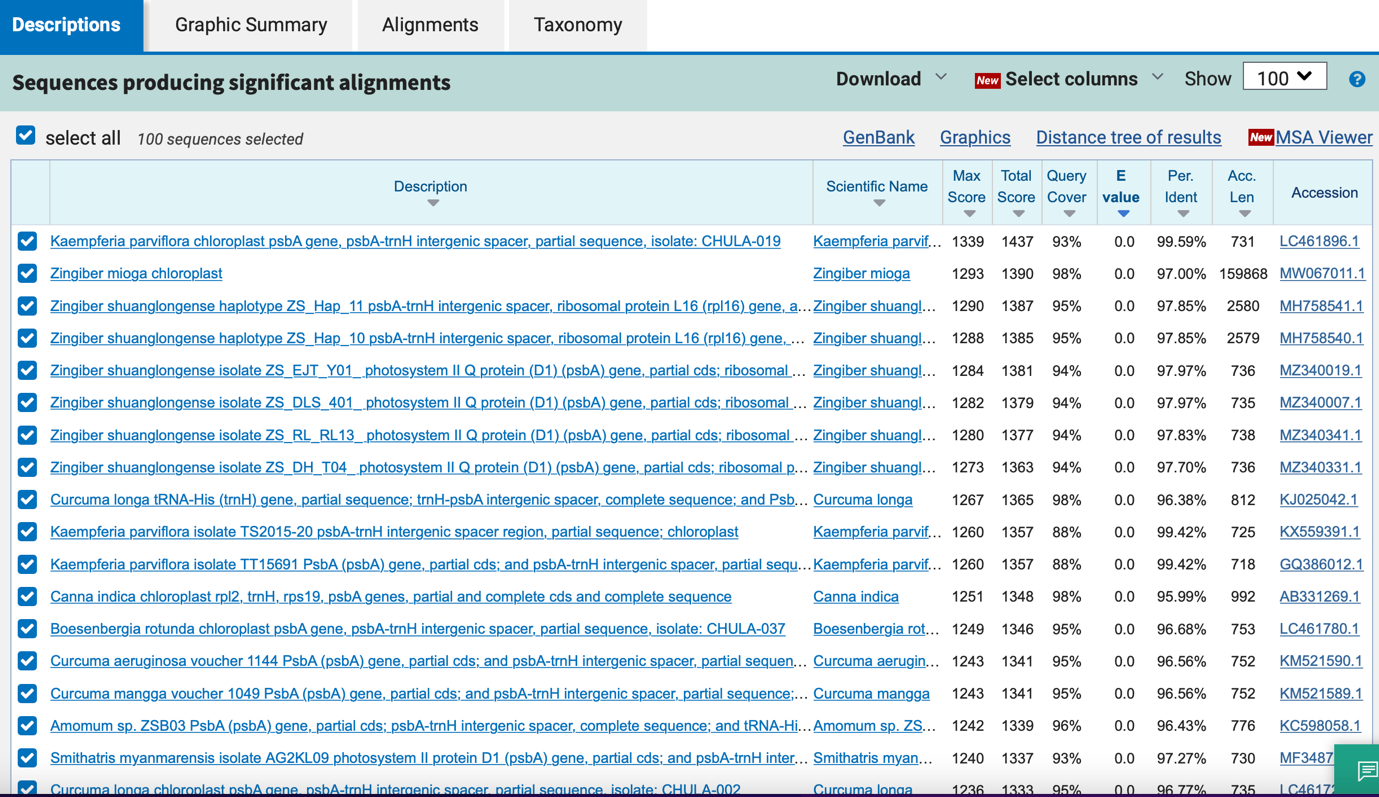


*rbc*L


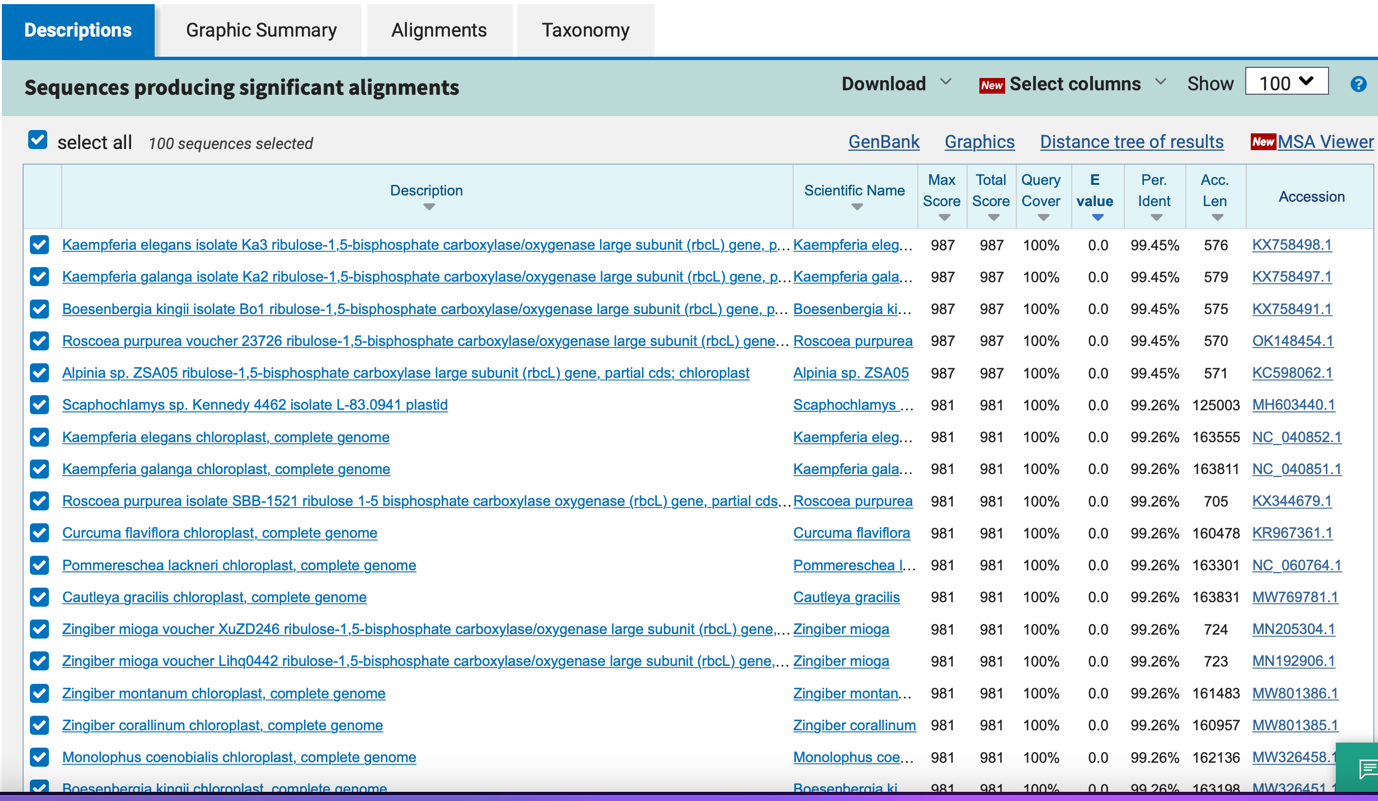


**17_** ***Centella asiatica***

ITS


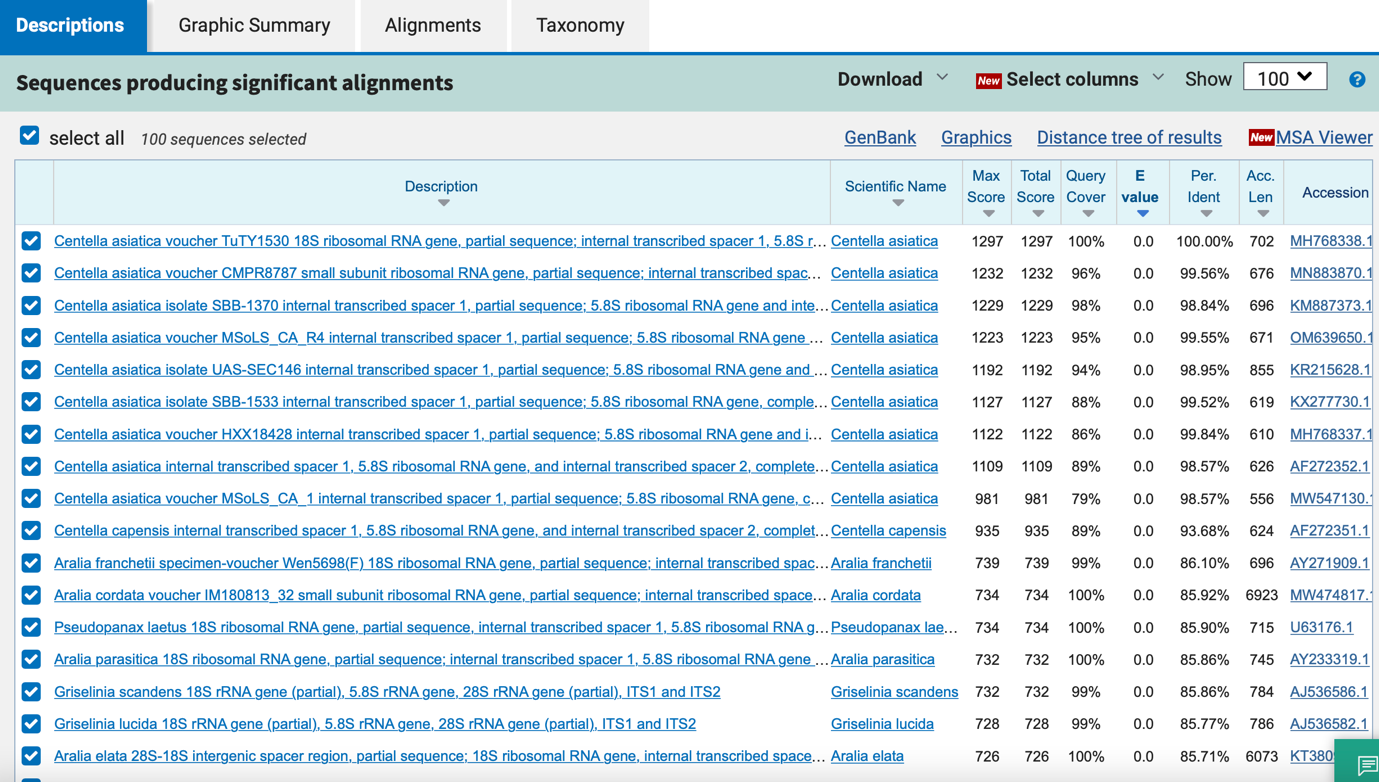


*mat*K


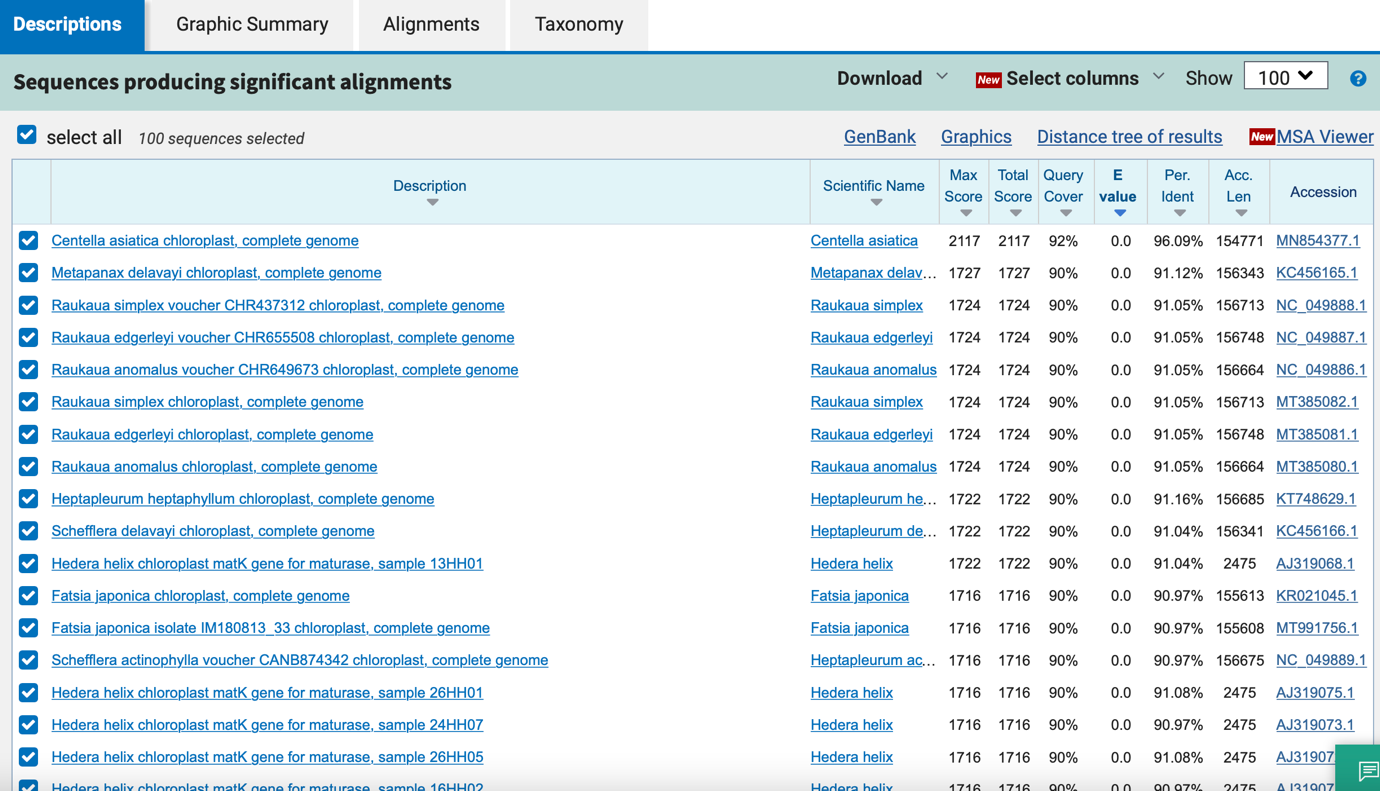


*psb*A_*trn*H


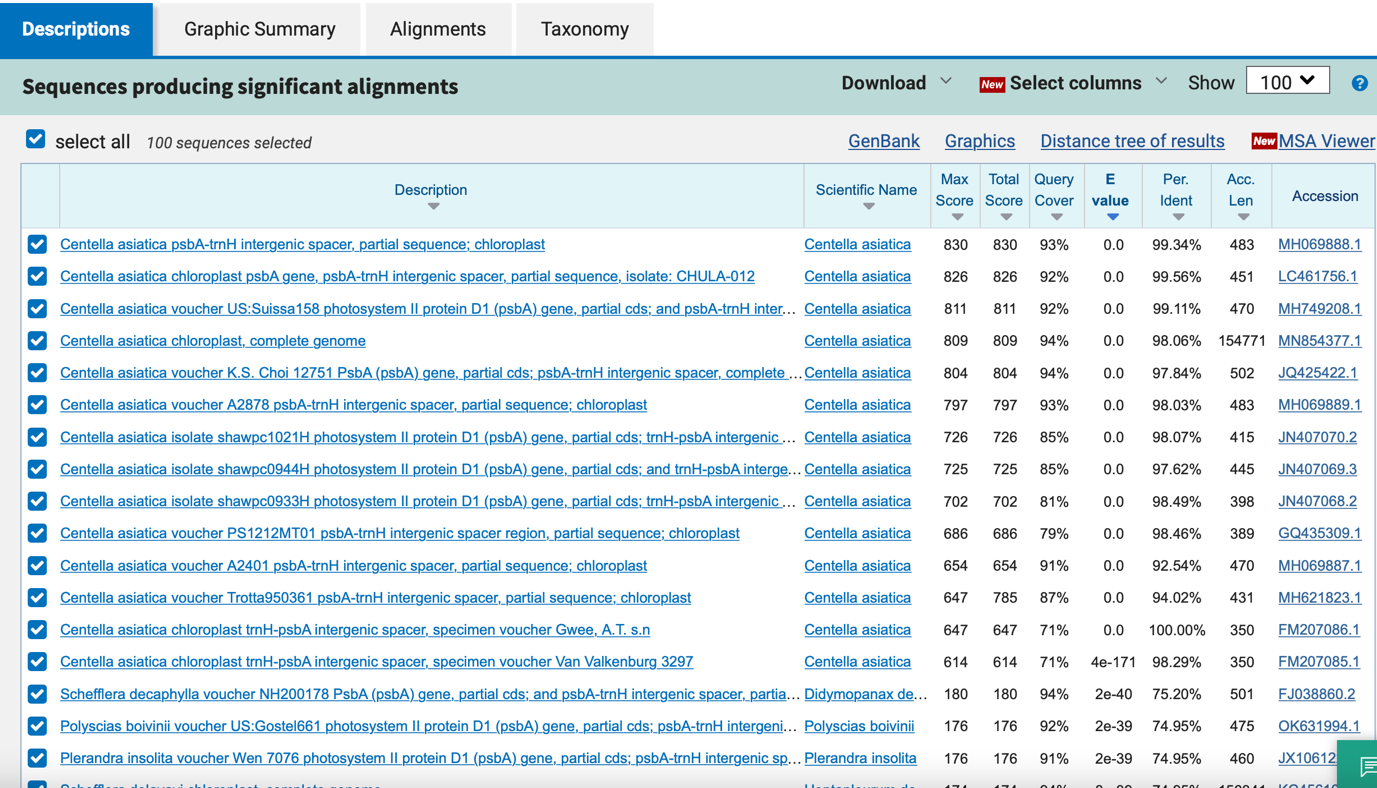


*rbc*L


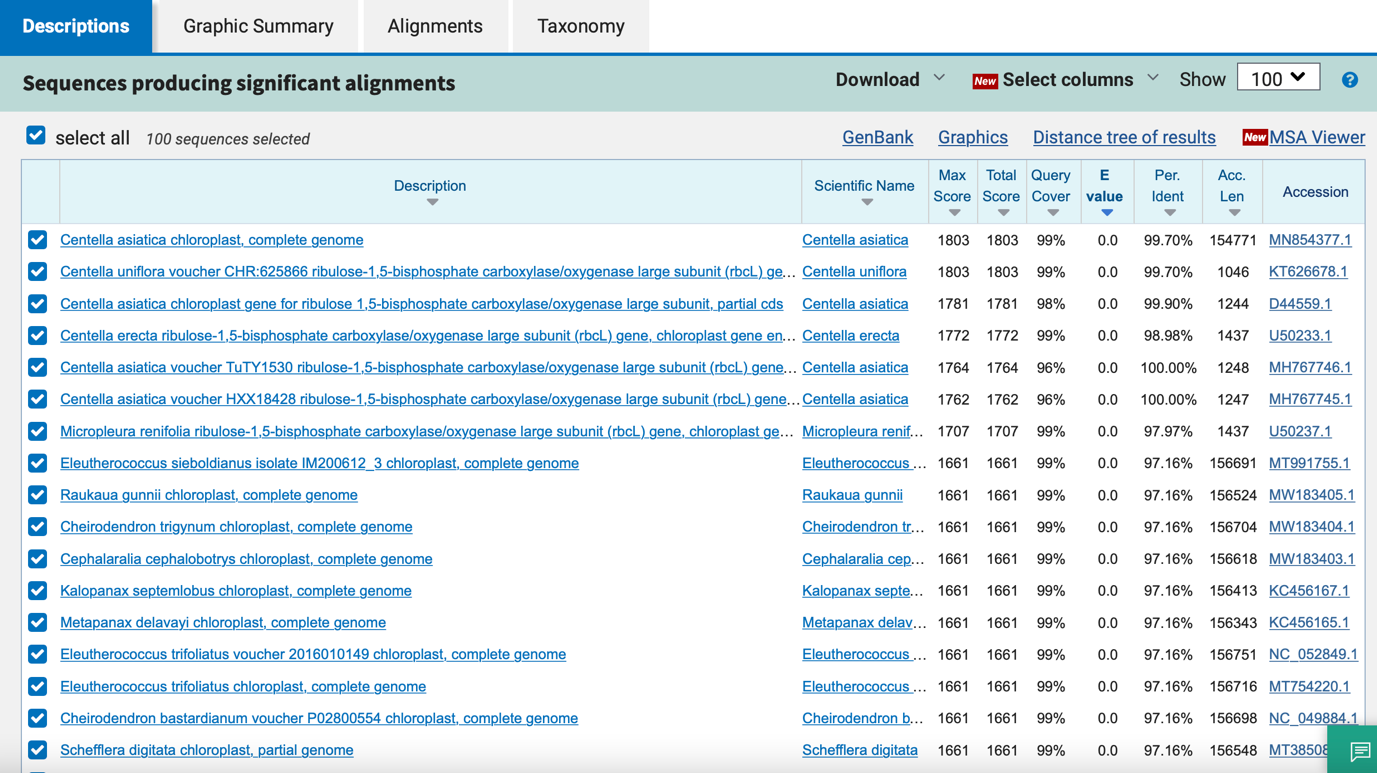


**18_** ***Centella asiatica***

ITS

*
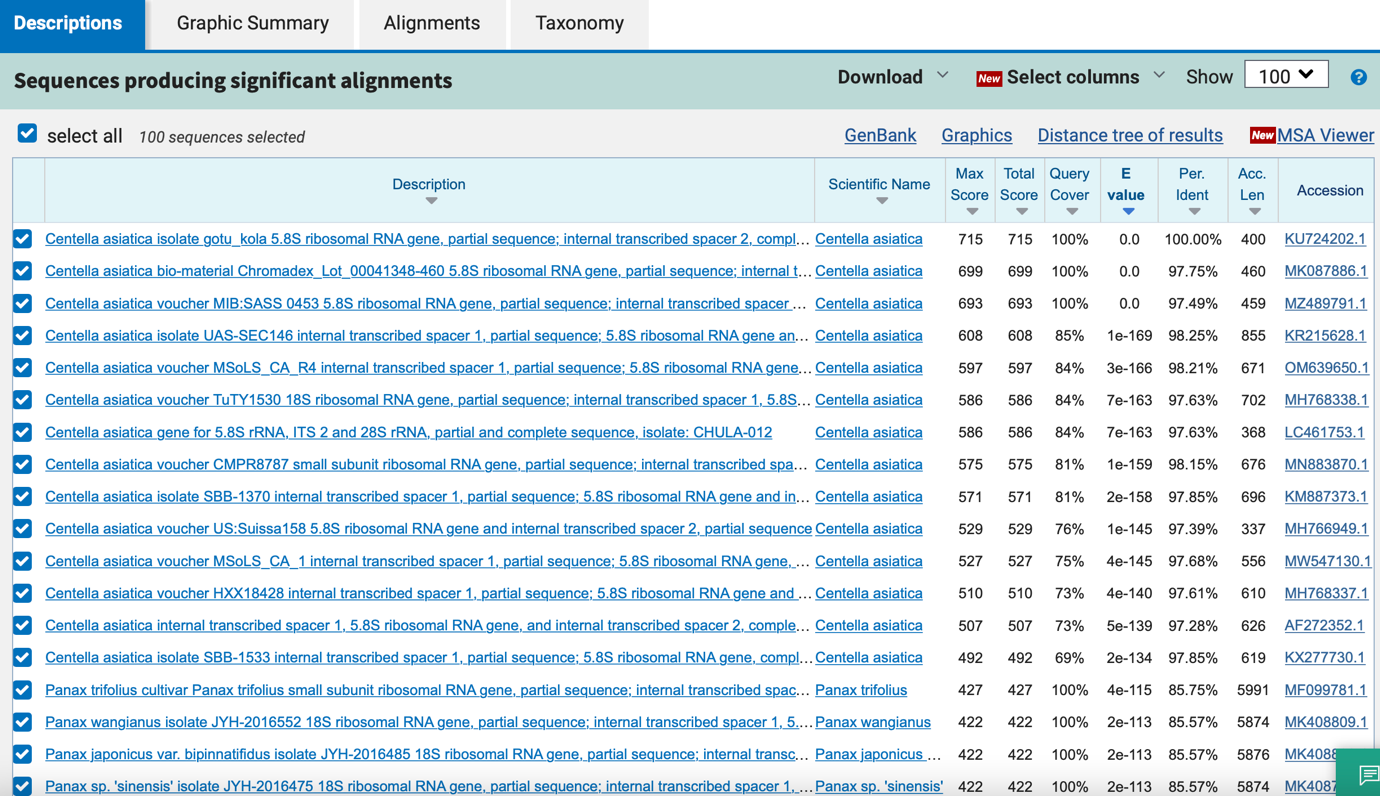
*

*mat*K


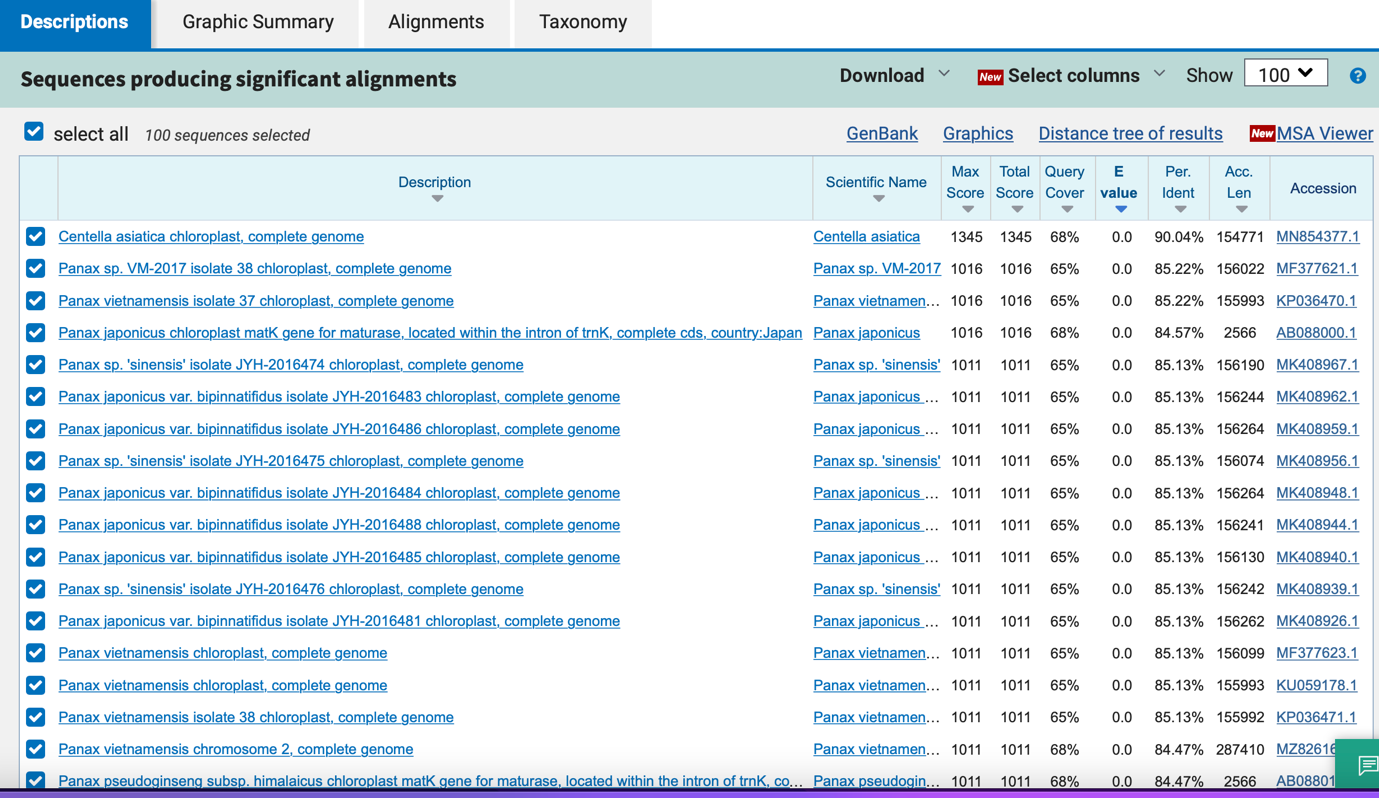


*psb*A_*trn*H


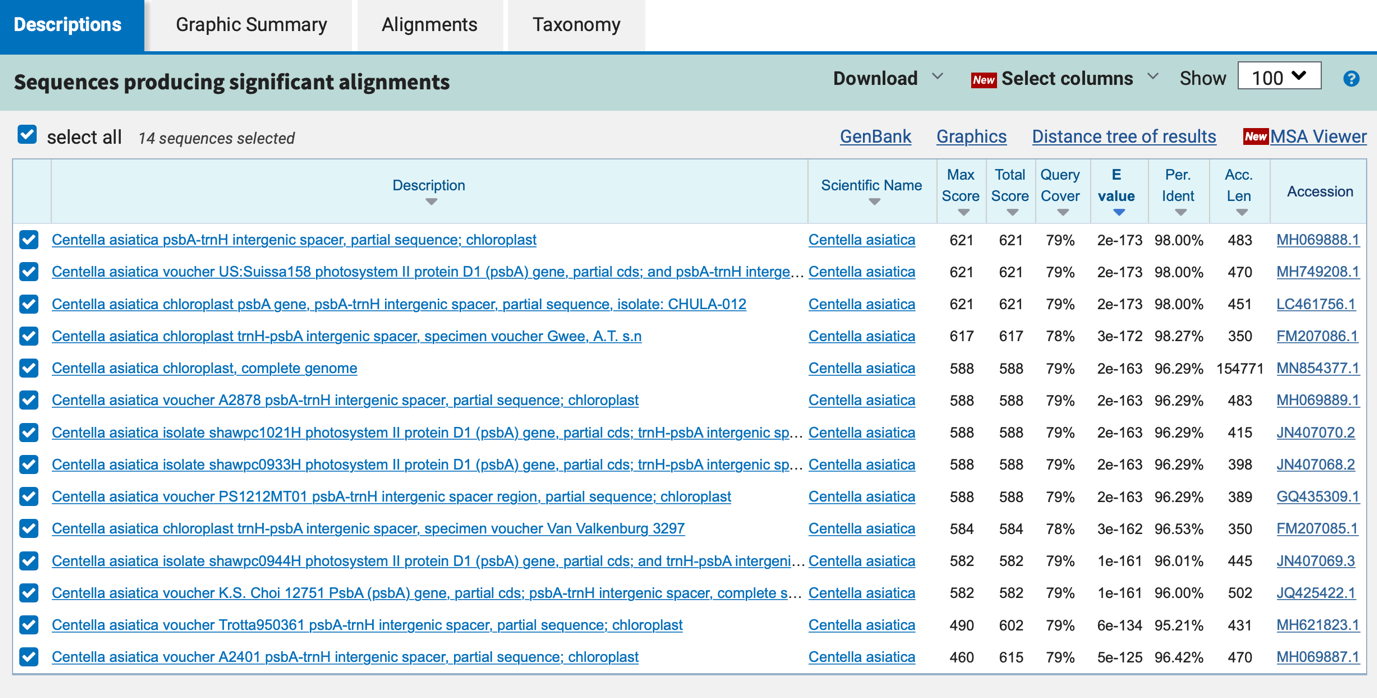


*rbc*L


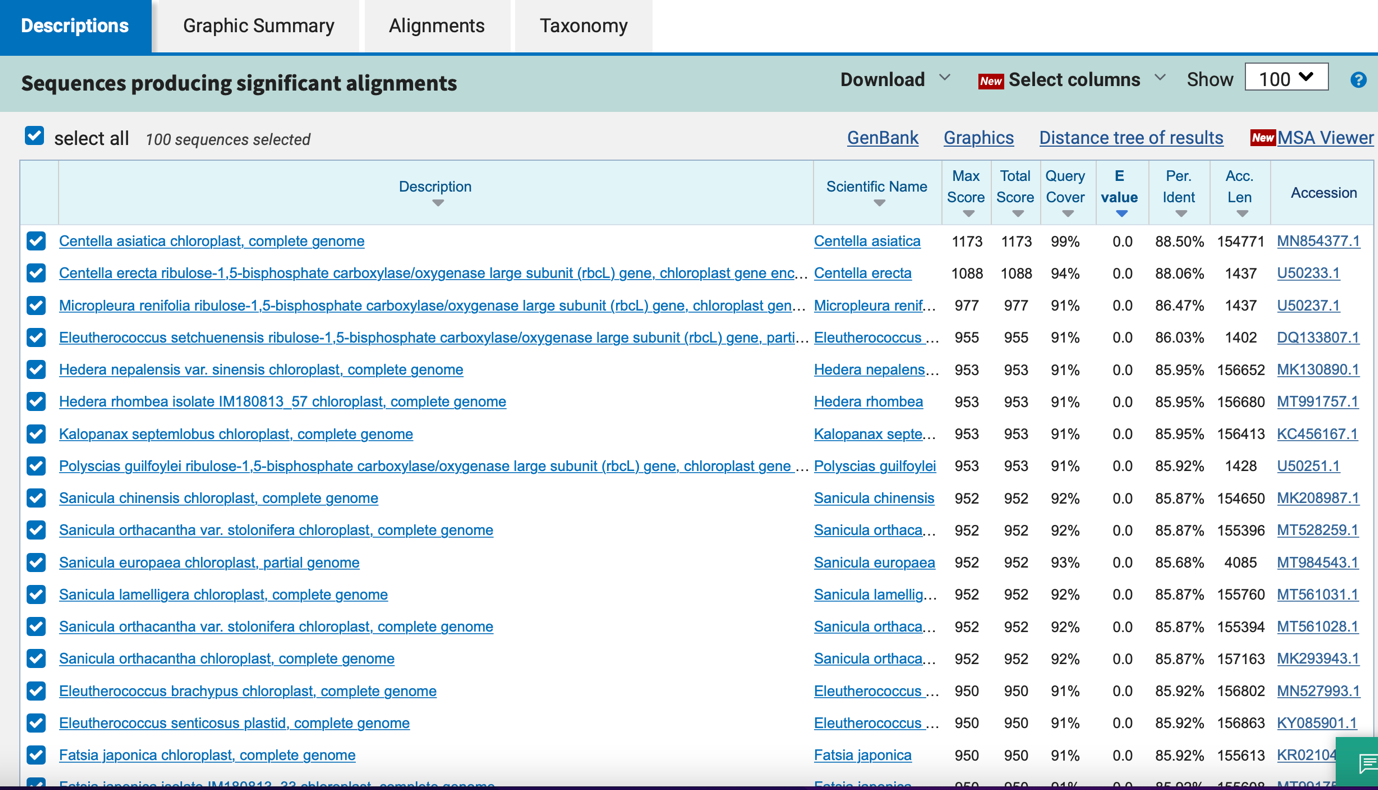


**19_** ***Curcuma longa***

ITS


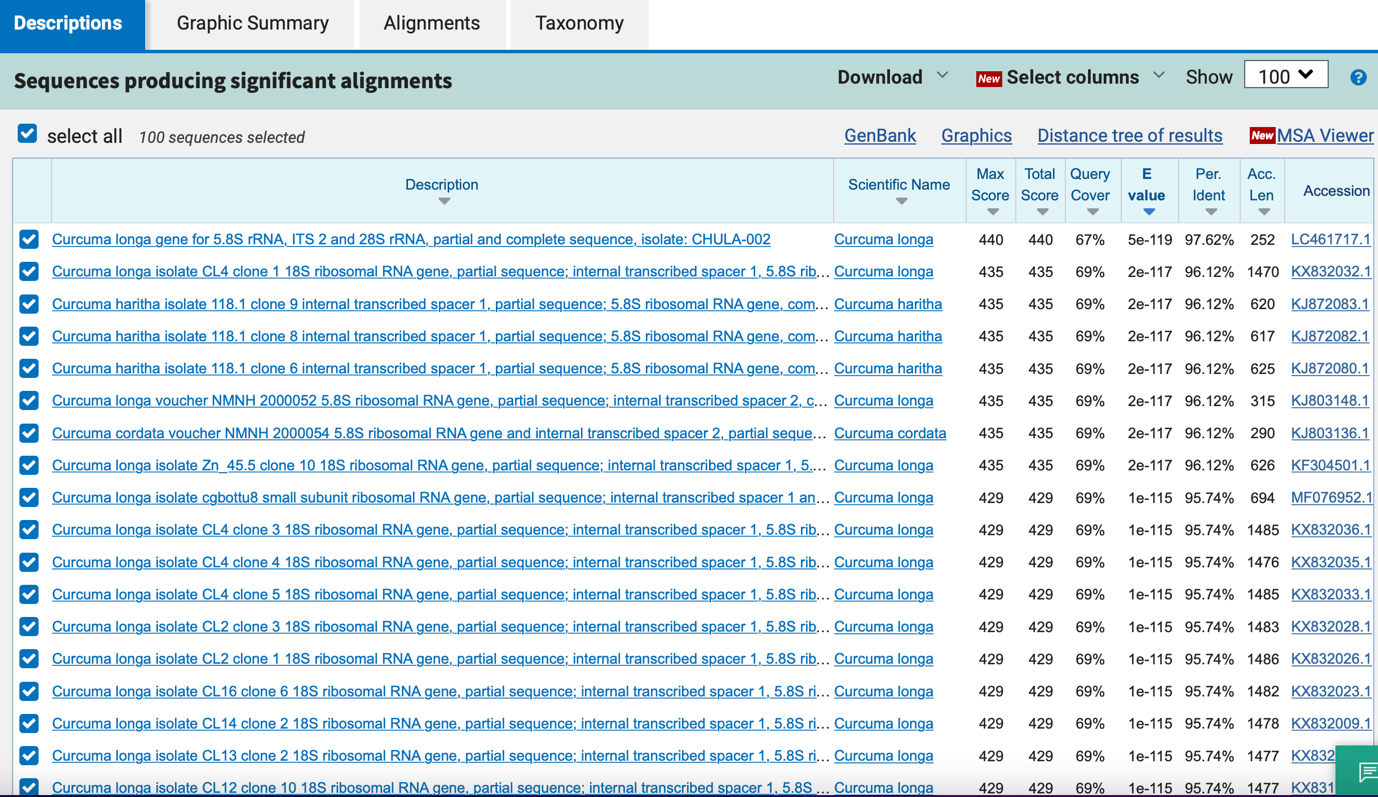


*mat*K

*
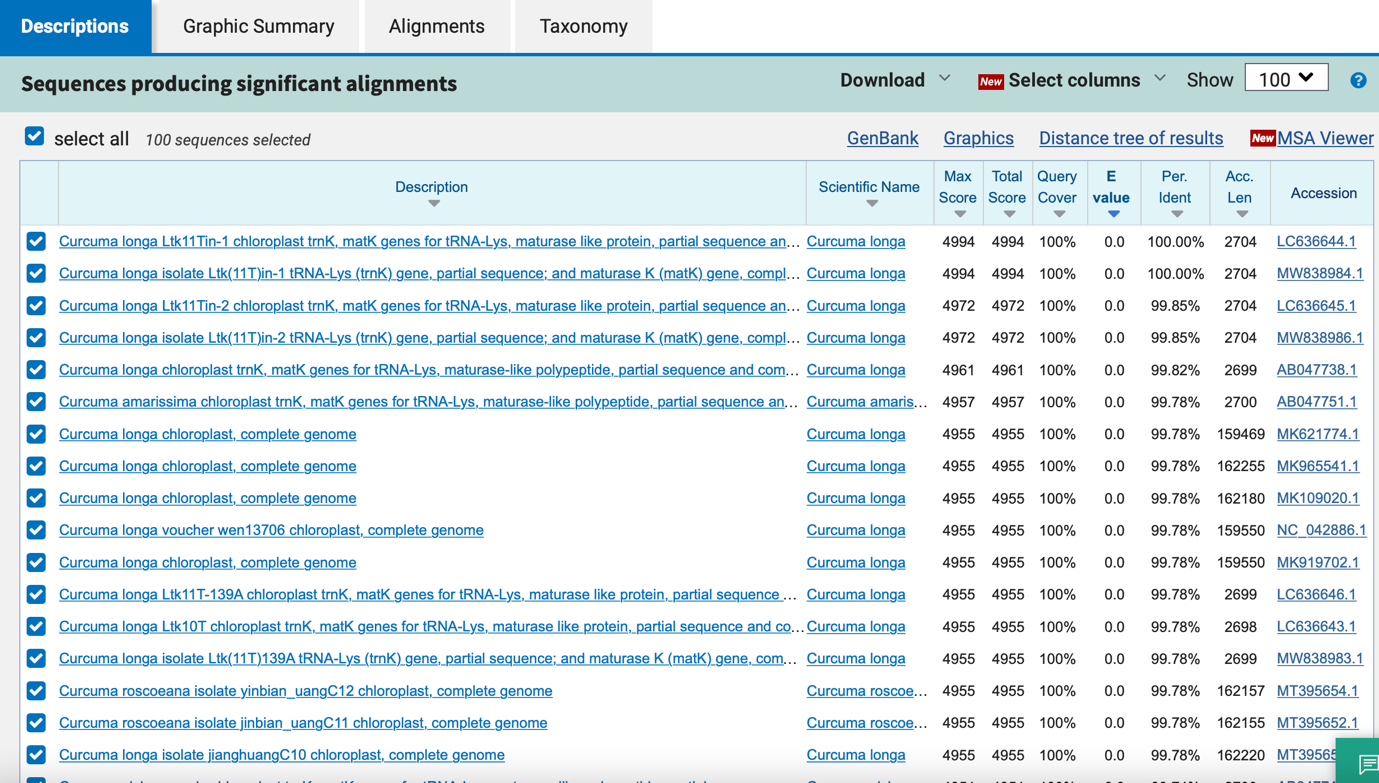
*

*psb*A_*trn*H


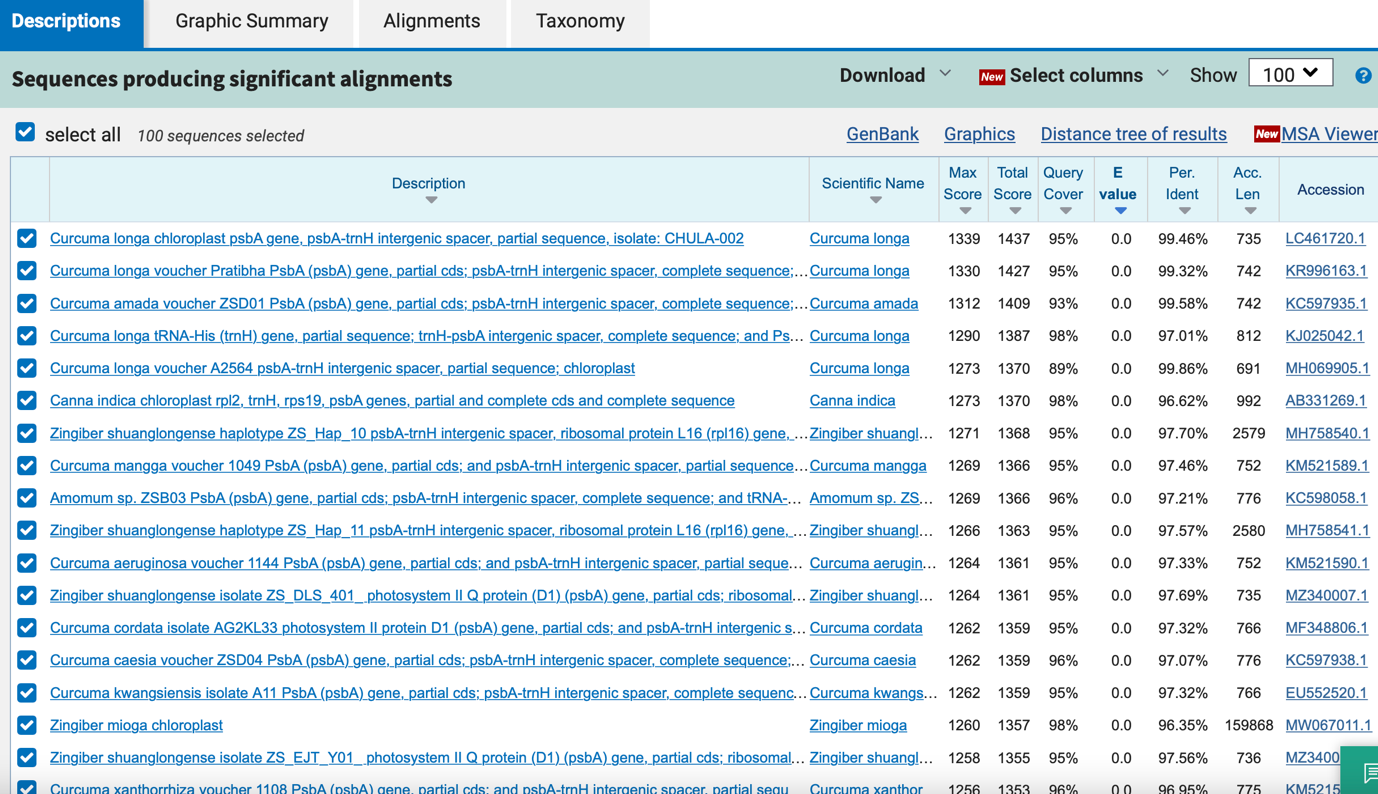


*rbc*L


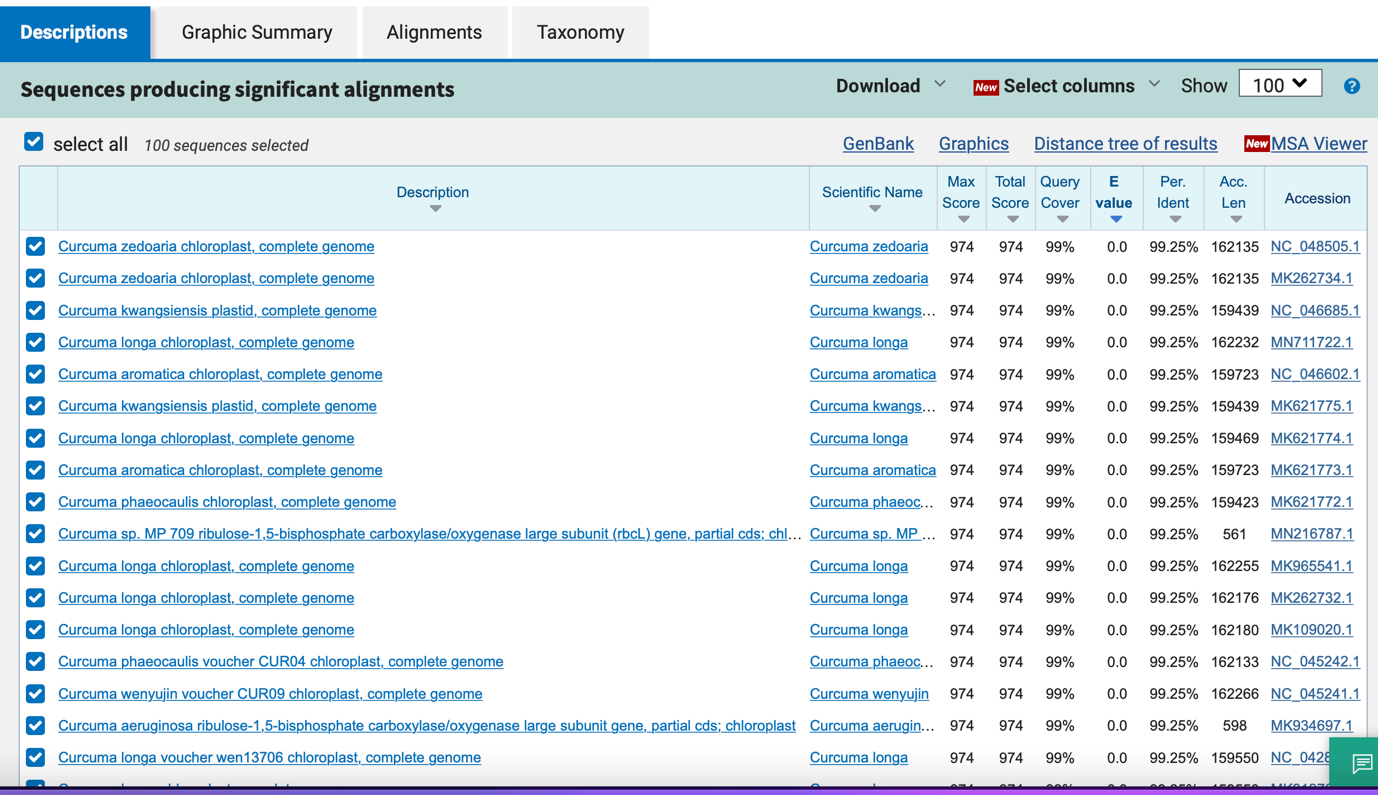


**20_** ***Zingiber montanum***

ITS


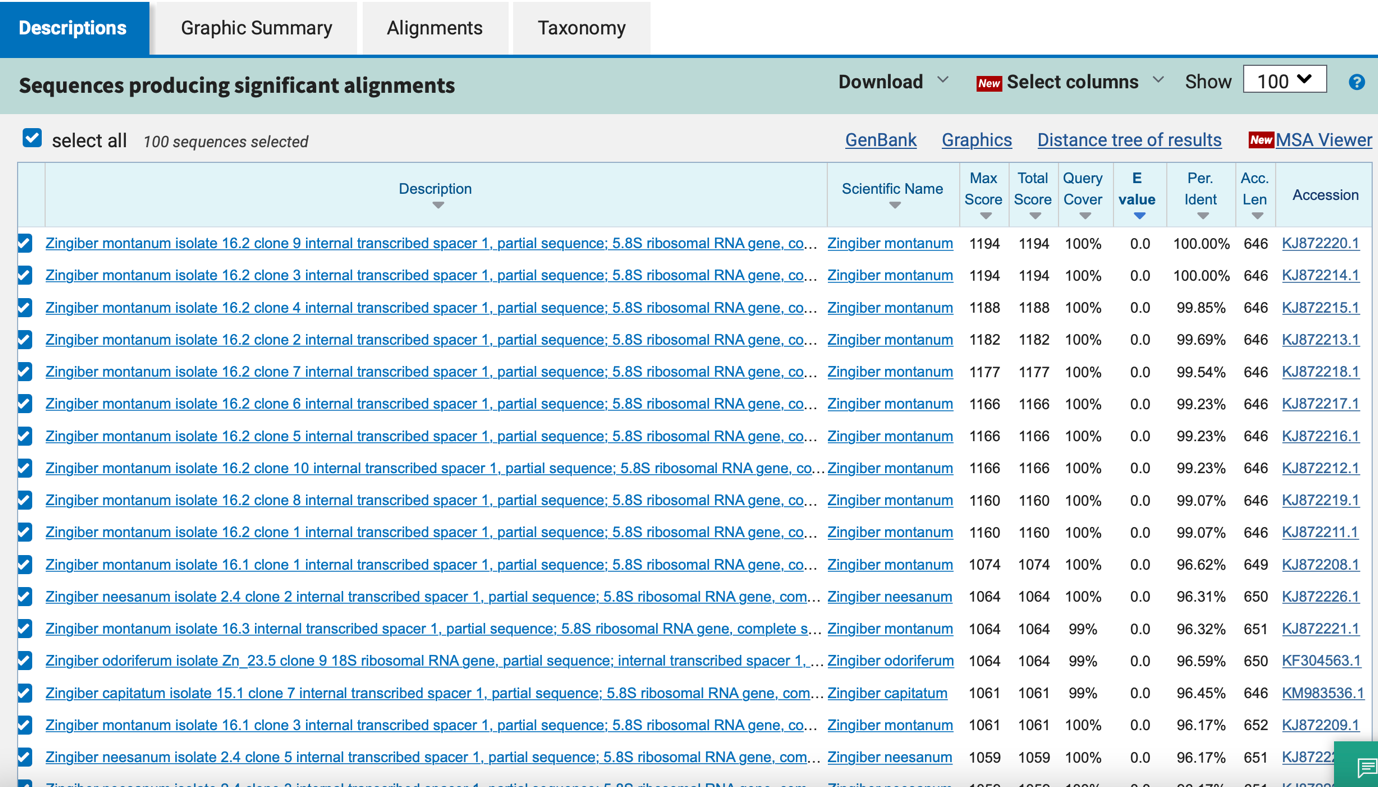


*mat*K


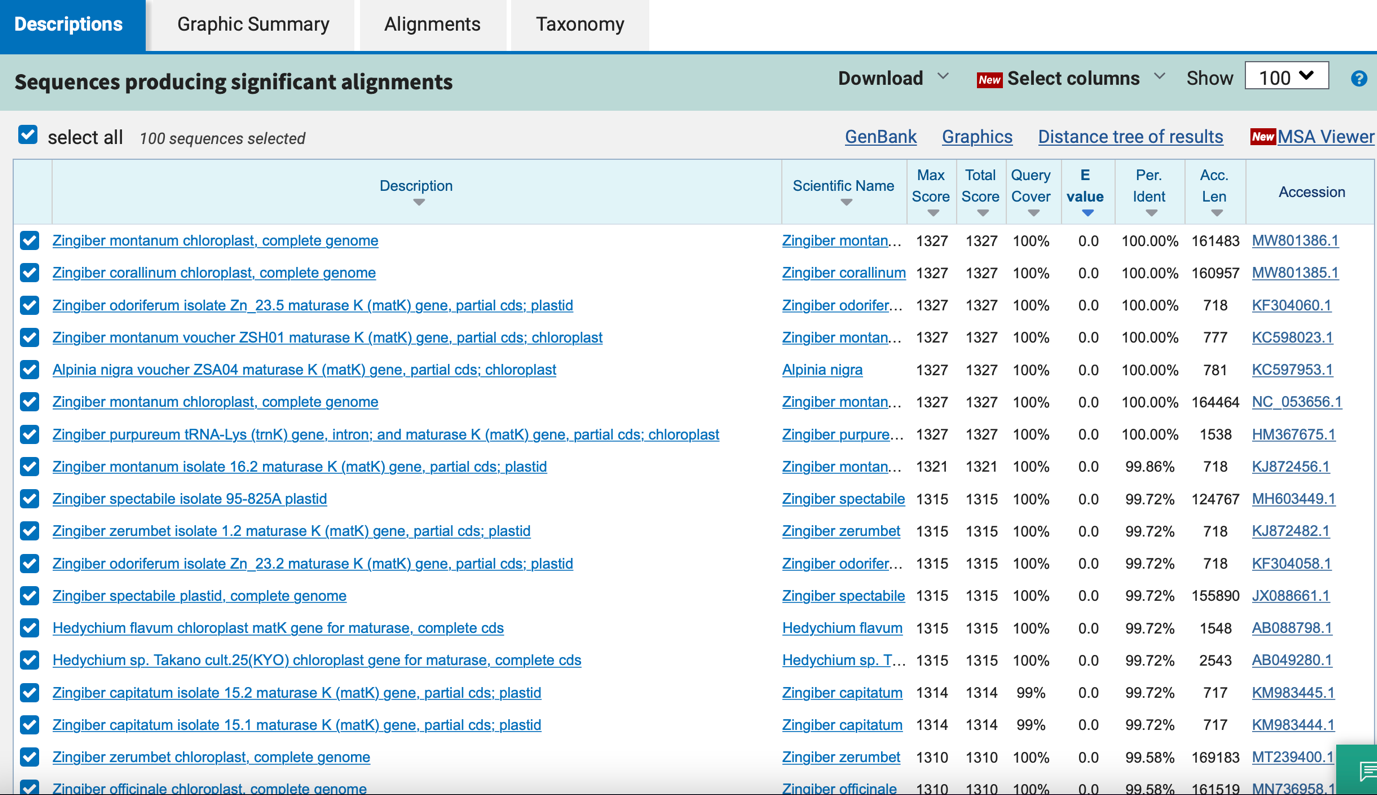


*psb*A_*trn*H


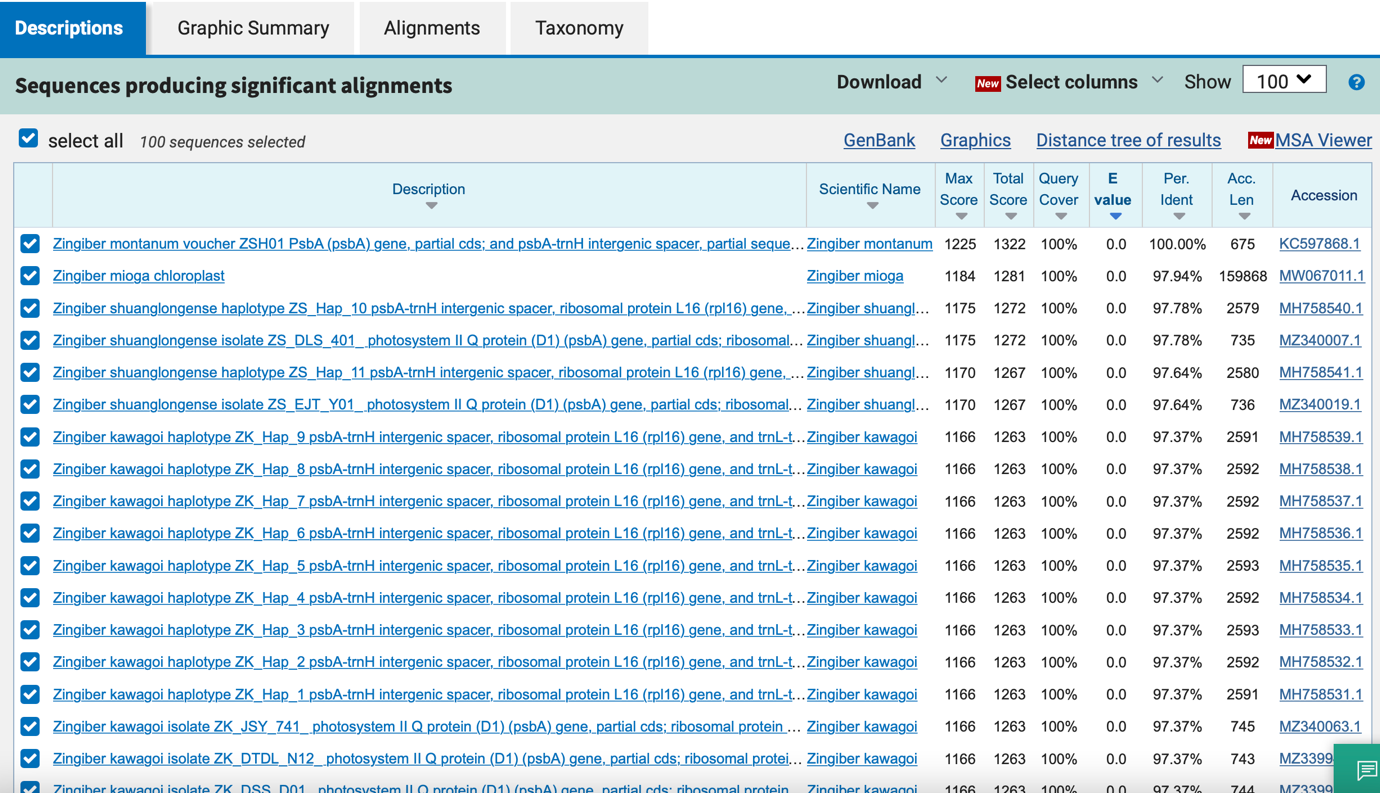


*rbc*L


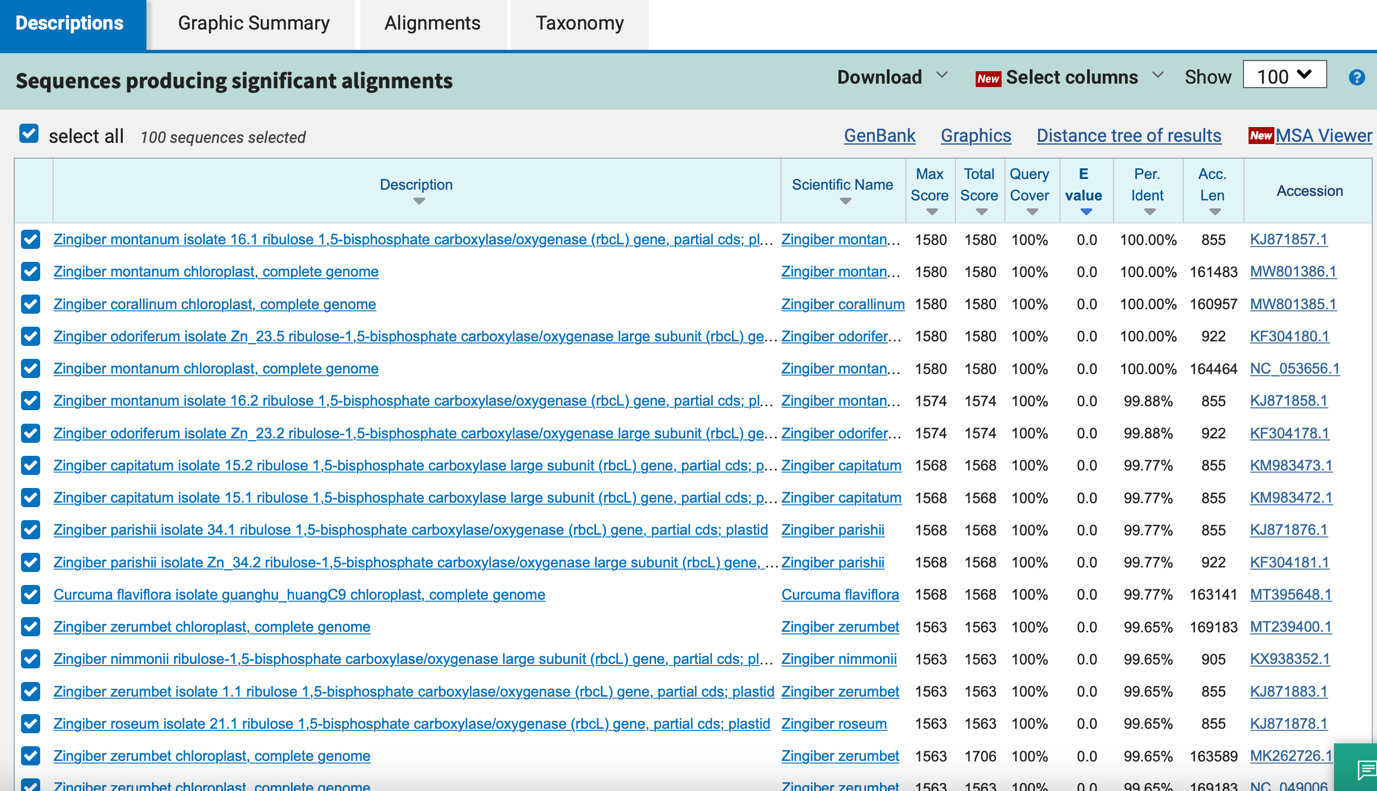

Supplement: Supplementary file 4 — Supplementary Table 3. [file 41598_2022_13287_MOESM4_ESM.docx]
